# Supplementary material for: Chemical Composition, Antioxidant Activities, Antidepressant Effect, and Lipid Peroxidation of Peruvian Blueberry: Molecular Docking Studies on Targets Involved in Oxidative Stress and Depression
Source: Plants (Basel). 2024 Jun 14;13(12):1643. doi: 10.3390/plants13121643 (PMC11207408; doi:10.3390/plants13121643)

Chemical Composition, Antioxidant Activities, Antidepressant Effect, and Lipid Peroxidation of Peruvian Blueberry: Molecular Docking Studies on Targets Involved in Oxidative Stress and Depression

Iván M. Quispe-Díaz [1], Roberto O. Ybañez-Julca [1*], Ricardo Pino-Ríos [2,3], José D. Quispe-Rodríguez [1], Daniel Asunción-Alvarez [1], Elena Mantilla-Rodríguez [1], Roger A. Rengifo-Penadillos [1], Edison Vásquez-Corales [4], Ricardo D.D.G. de Albuquerque [1], Wilfredo O. Gutiérrez-Alvarado [5], Julio Benites [2,3*]

[1] Facultad de Farmacia y Bioquímica, Universidad Nacional de Trujillo, Trujillo 13011, Perú; iquispe@unitru.edu.pe (I.M.Q.-D.); rybanez@unitru.edu.pe (R.O.Y.-J.); hasuncion@unitru.edu.pe (D.A.-A.); amantilla@unitru.edu.pe (E.M.-R); rrengifo@unitru.edu.pe (R.A.R.-P); rgalhardod@unitru.edu.pe (R.D.D.G.A)

[2] Laboratorio de Química Medicinal, Química y Farmacia, Facultad de Ciencias de la Salud, Universidad Arturo Prat, Casilla 121, Iquique 1100000, Chile; juliob@unap.cl (J.B.)

[3] Instituto de Química Medicinal, Universidad Arturo Prat, Casilla 121, Iquique 1100000, Chile; rpinorios@unap.cl (R.P.-R.)

[4] Escuela de Farmacia y Bioquímica, Universidad Católica Los Ángeles de Chimbote, Chimbote 02801, Perú; evasquezc@uladech.edu.pe (E.V.-C.)

[5] Facultad de Farmacia y Bioquímica, Universidad Nacional de la Amazonía Peruana, Iquitos 16001, Perú; Wilfredo.gutierrez@unapiquitos.edu.pe (W.O.G.-A)

* Correspondence: rybanez@unitru.edu.pe (R.O.Y.-J.); juliob@unap.cl (J.B.); Tel.: +51-0449-7634-5993 (R.O.Y.-J.); Tel.: +56-57-2252-6275 (J.B.)

Figure S1. Compounds spectra **1–11** identified in *Vaccinium corymbosum* L.

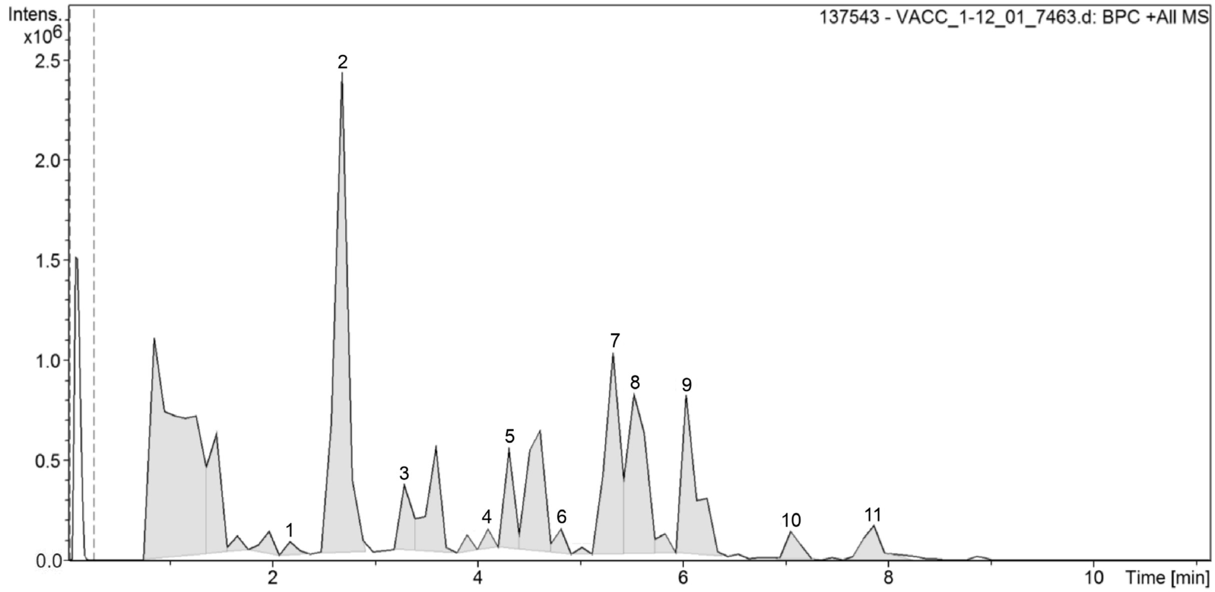

Compound 1

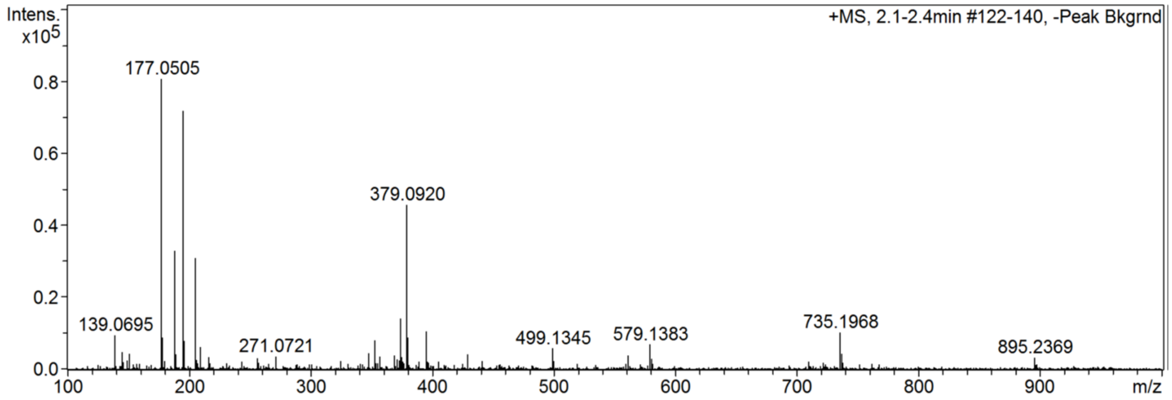

Compound 2

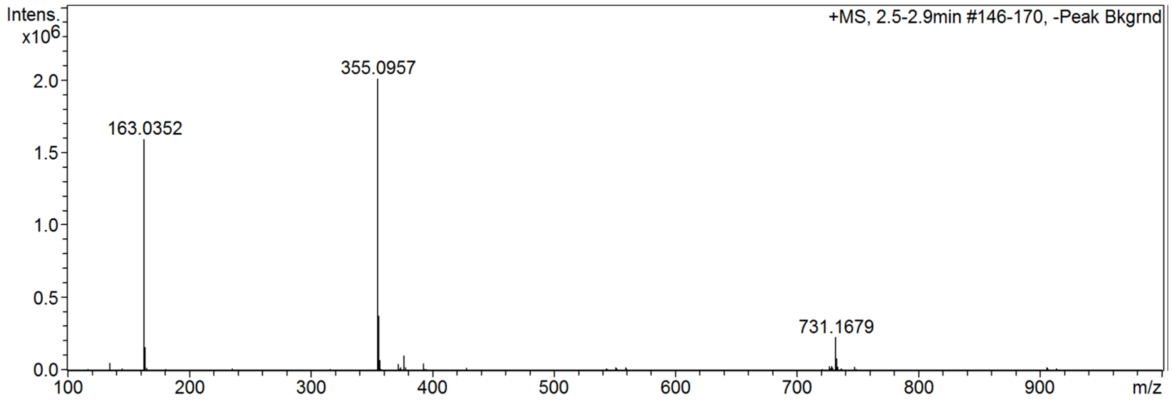

Compound 3

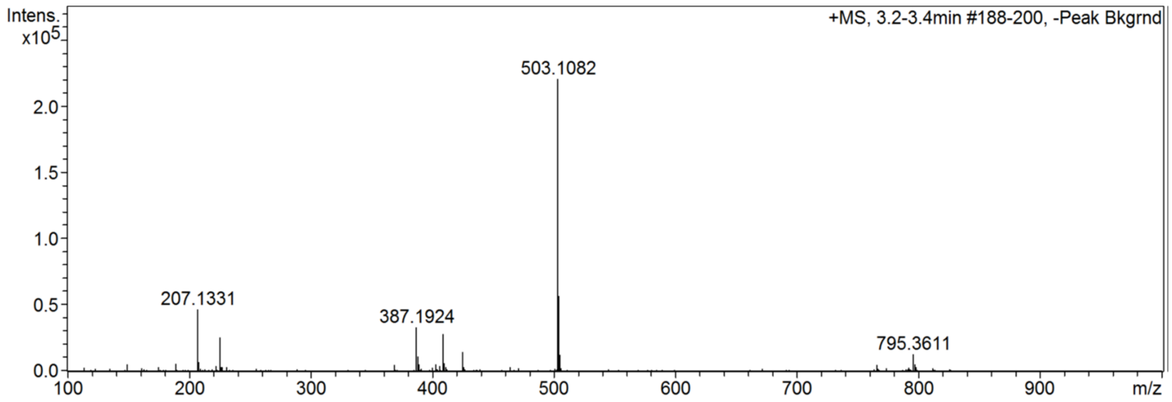

Compound 4

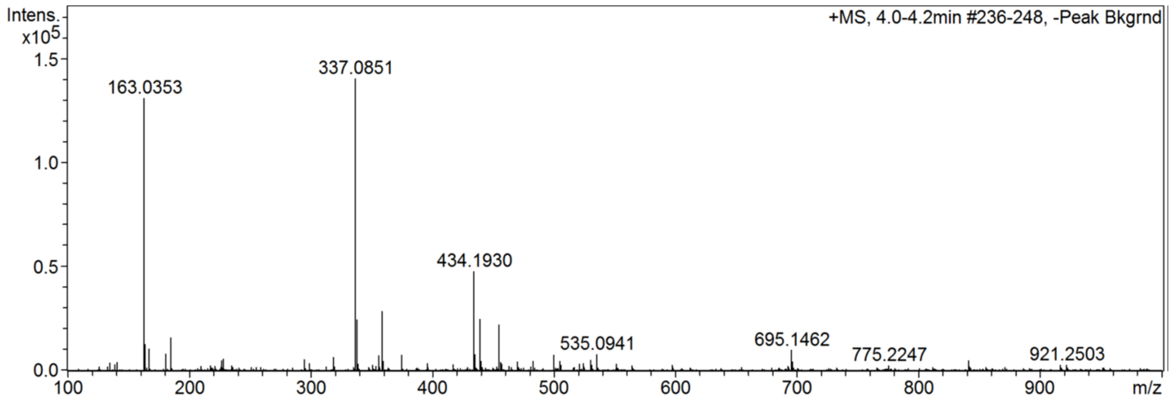

Compound 5

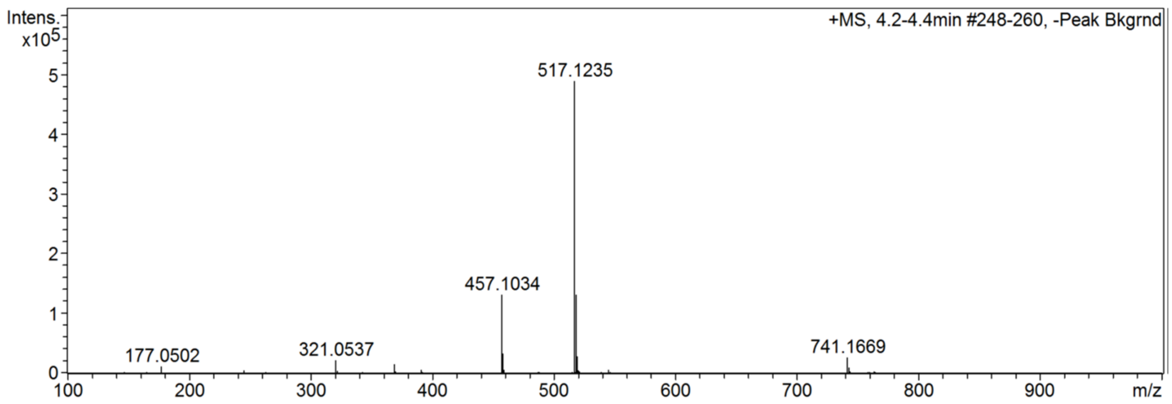

Compound 6

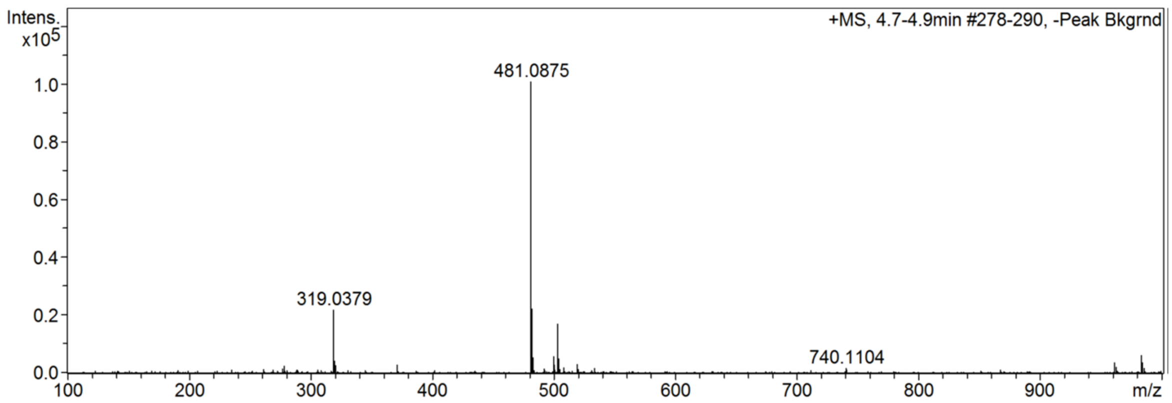

Compound 7

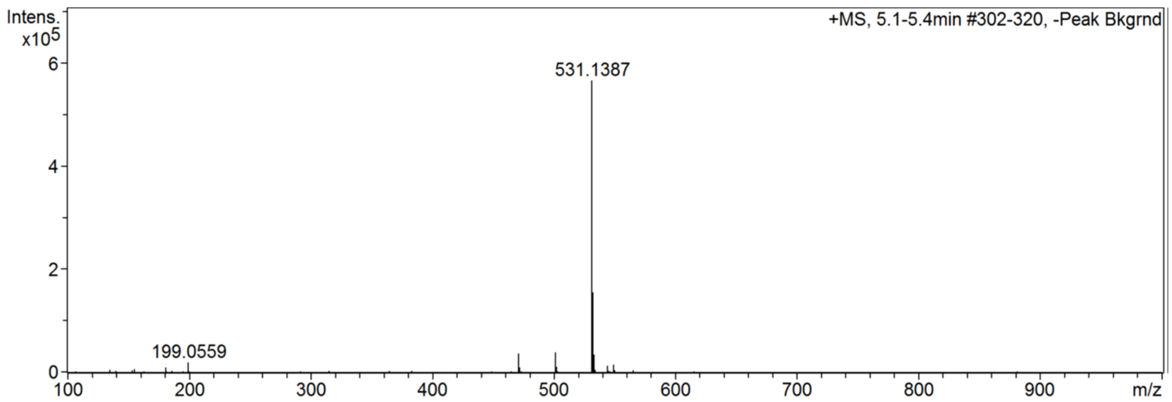

Compound 8

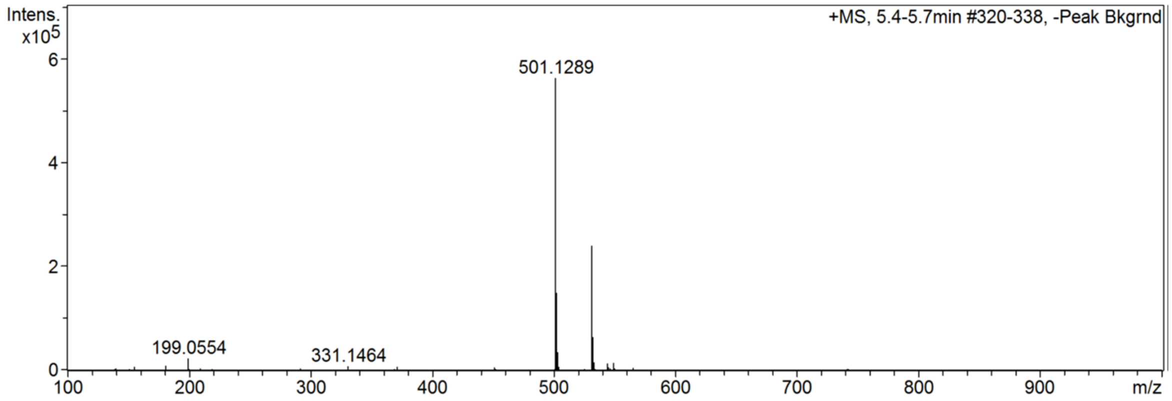

Compound 9

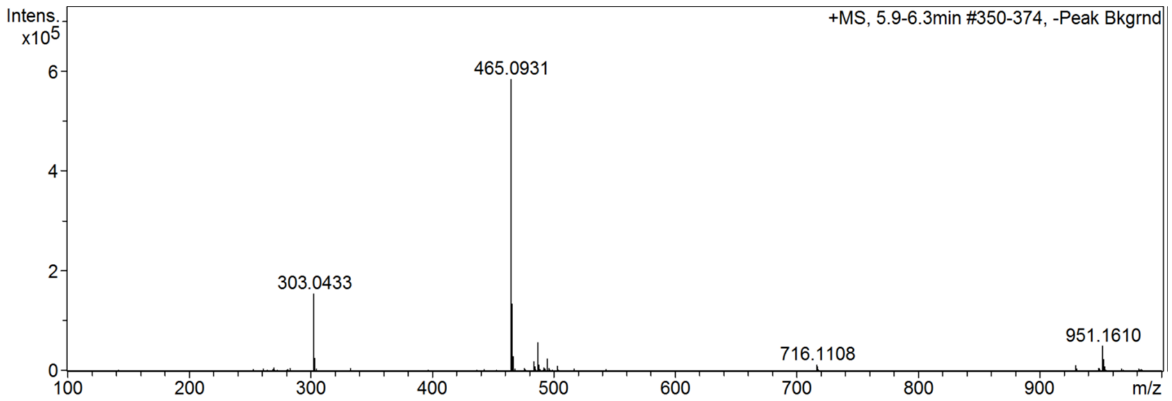

Compound 10

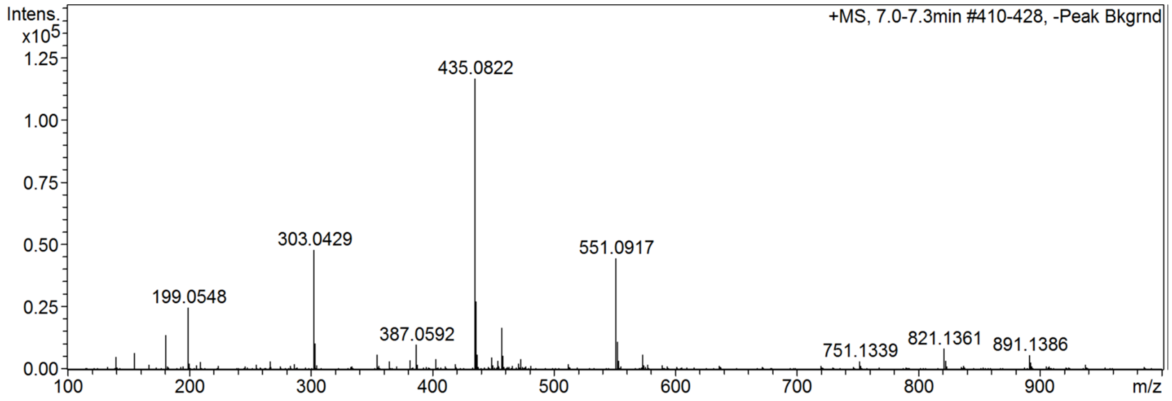

Compound 11

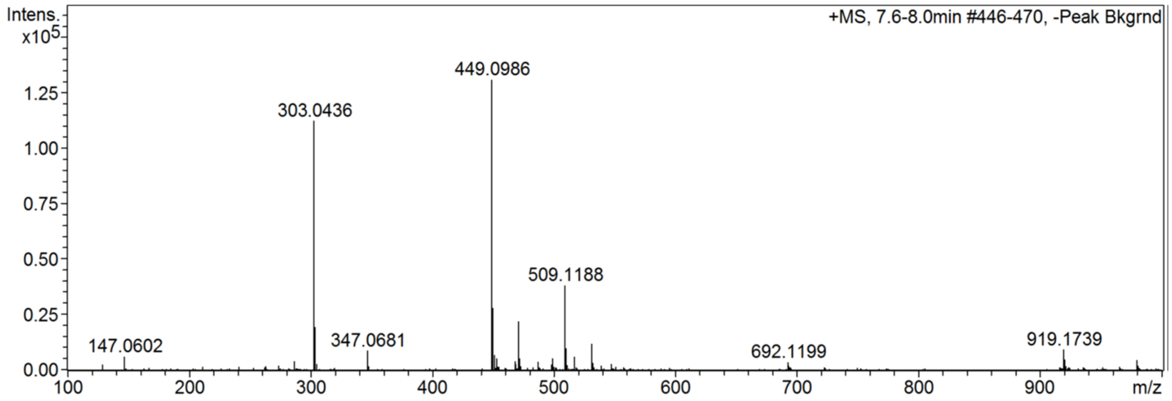

Table S1. Canonical SMILES of 11 molecules identified in *Vaccinium corymbosum* L. fruits extract

N°	Canonical SMILES
1	OCc1cc2ccccc2oc1=O
2	O[C@]1(C(=O)O)C[C@@H](OC(=O)/C=C/c2cc(O)c(O)cc2)[C@H](O)[C@H](O)C1
3	O=C(C(C(O)=O)(O)C(O)(C(O)=O)C(/C=C/C1=CC=C(O)C=C1)=O)/C=C/C2=CC(OC)=C(O)C(OC)=C2
4	O[C@@H]1C=C(C(=O)O)C[C@@H](OC(=O)/C=C/c2ccc(O)c(O)c2)[C@@H]1O
5	O[C@]1(C(=O)O)C[C@@H](OC(=O)/C=C/c2cc(O)c(O)cc2)[C@H](O)[C@H](OC(=O)/C=C/c2ccc(O)c(O)c2)C1
6	O=c1c2c(O)cc(O)cc2oc(c2cc(O)c(O)c(O)c2)c1O[C@@H]1C[C@H](CO)[C@@H](O)[C@H](O)[C@H]1O
7	O[C@]1(C(=O)OC)C[C@@H](OC(=O)/C=C/c2cc(O)c(O)cc2)[C@H](O)[C@H](OC(=O)/C=C/c2ccc(O)c(O)c2)C1
8	O[C@@H]1[C@H](OC(=O)/C=C/c2ccc(O)cc2)C[C@@](O)(C(=O)O)C[C@H]1OC(=O)/C=C/c1cc(O)c(O)cc1

9	O=c1c2c(O)cc(O)cc2oc(c2ccc(O)c(O)c2)c1O[C@@H]1O[C@H](CO)[C@@H](O)[C@H](O)[C@H]1O
10	O=c1c2c(O)cc(O)cc2oc(c2cc(O)c(O)cc2)c1O[C@@H]1OC[C@H](O)[C@H](O)[C@H]1O
11	O=c1c2c(O)cc(O)cc2oc(c2cc(O)c(O)cc2)c1O[C@@H]1O[C@@H](C)[C@H](O)[C@@H](O)[C@H]1O

Figure S2. 2D representation of the interactions between Compound **1** – NADPH oxidase (PDB ID: 2CDU) complex. Hydrogen atoms have been omitted in some cases for clarity.

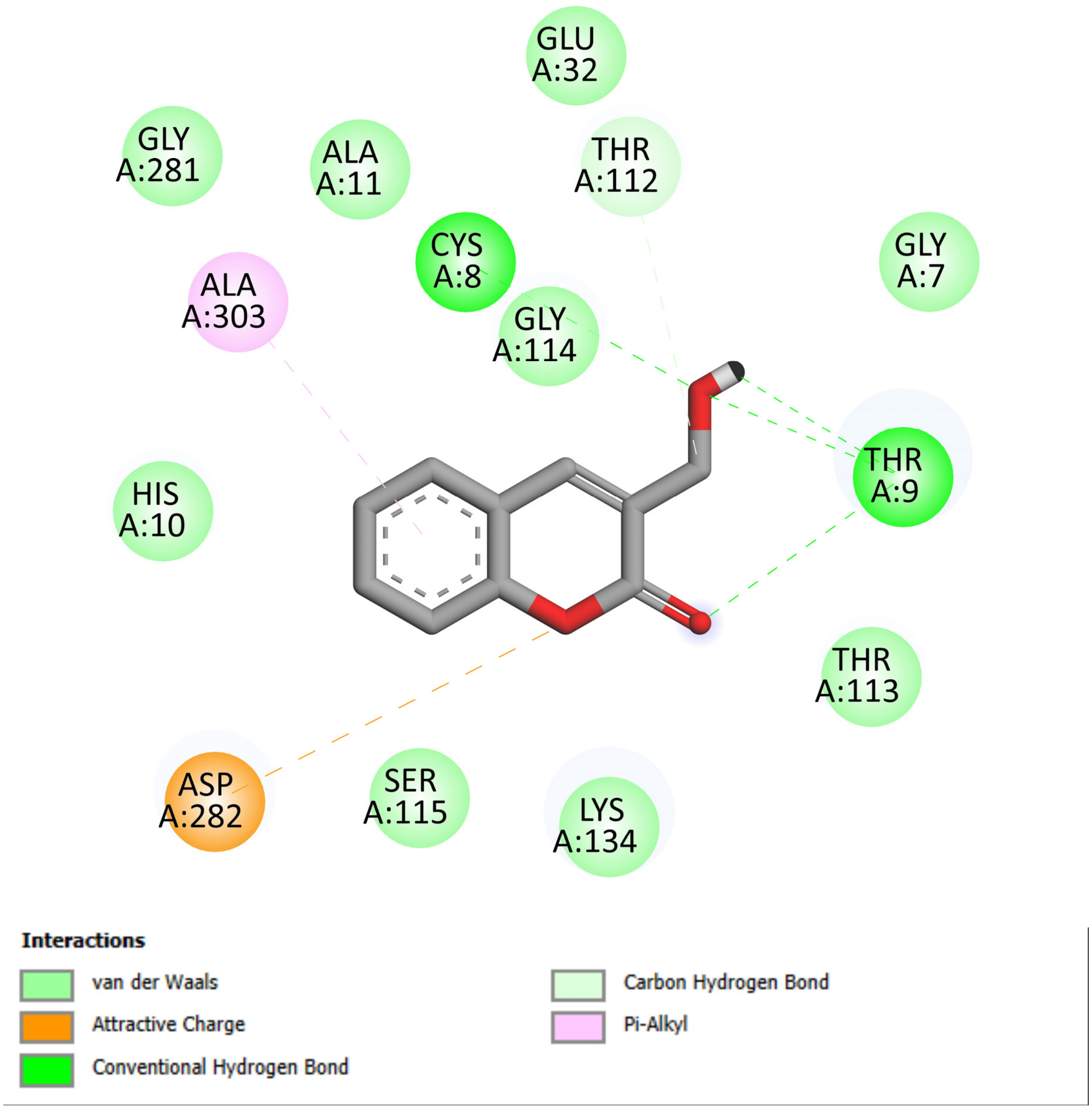

Figure S3. 2D representation of the interactions between Compound **2** – NADPH (PDB ID: 2CDU) oxidase complex. Hydrogen atoms have been omitted in some cases for clarity.

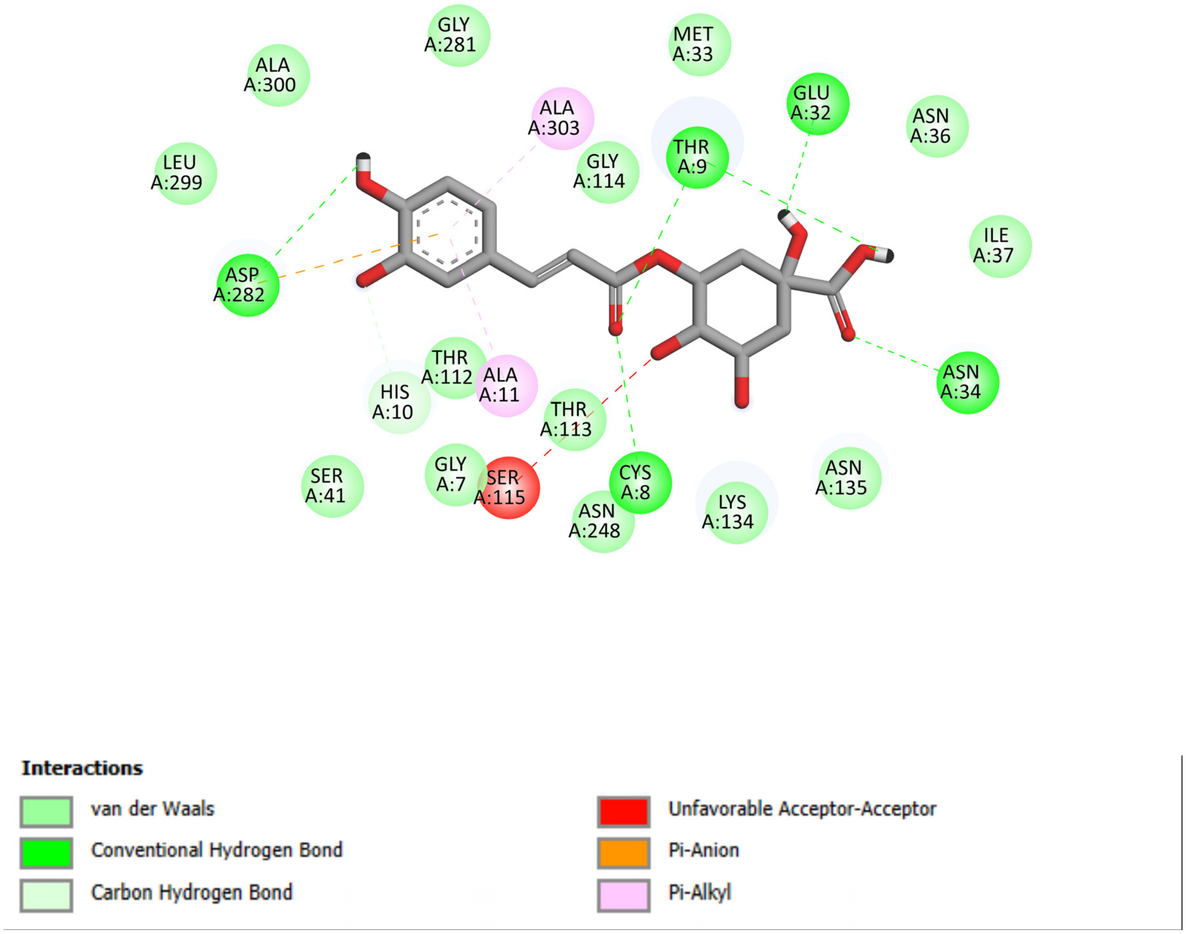

Figure S4. 2D representation of the interactions between Compound **3** – NADPH (PDB ID: 2CDU) oxidase complex. Hydrogen atoms have been omitted in some cases for clarity.

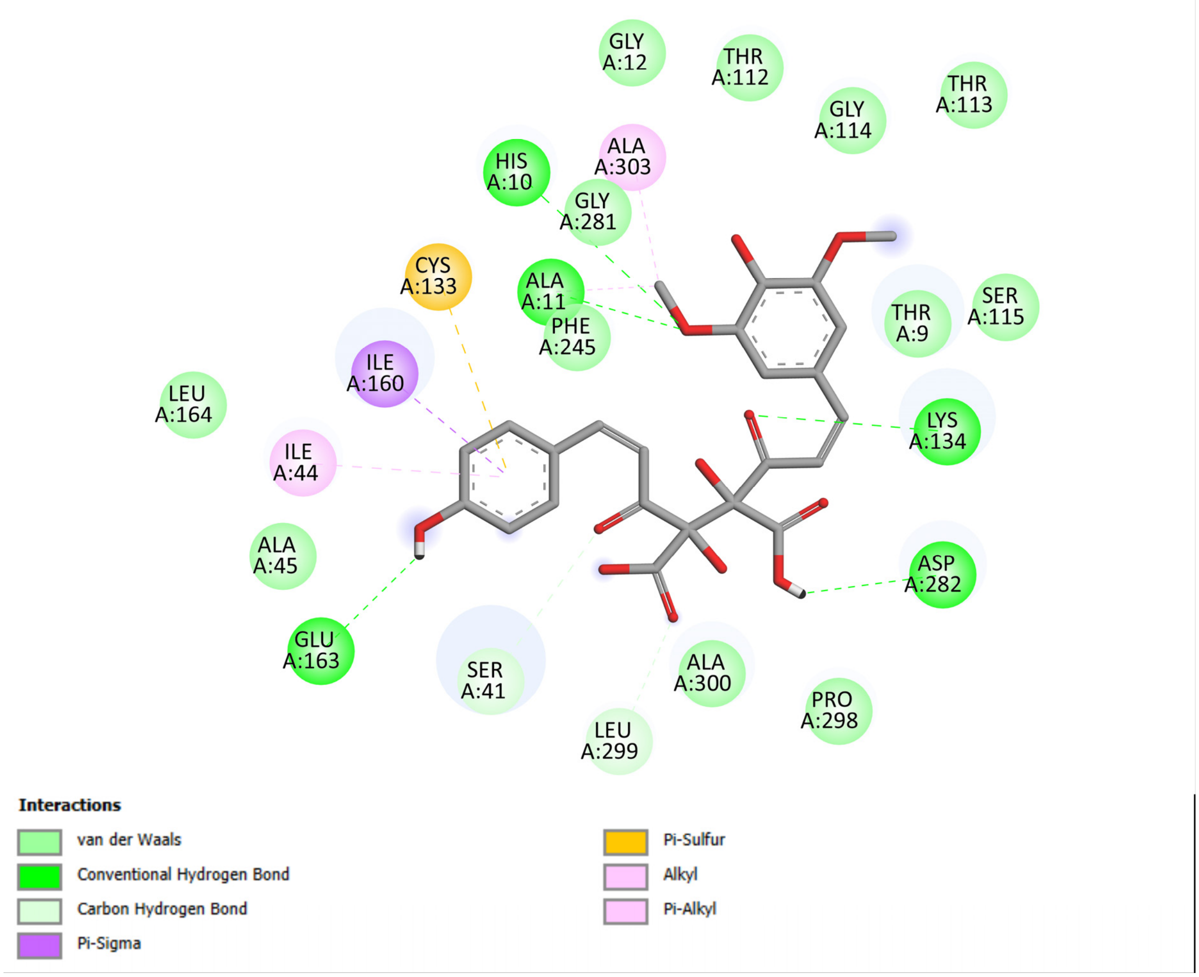

Figure S5. 2D representation of the interactions between Compound **4** – NADPH (PDB ID: 2CDU) oxidase complex. Hydrogen atoms have been omitted in some cases for clarity.

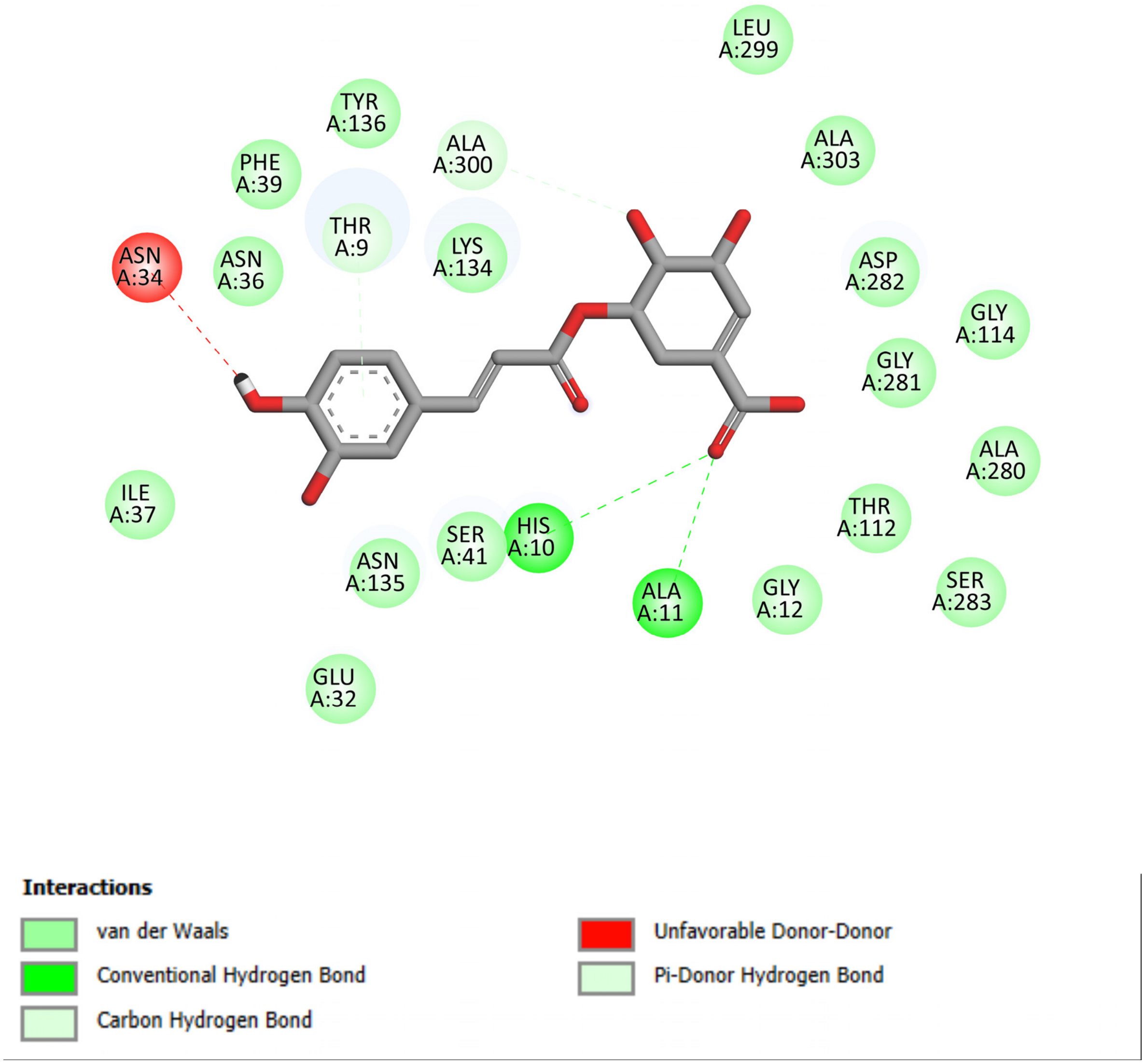

Figure S6. 2D representation of the interactions between Compound **5** – NADPH (PDB ID: 2CDU) oxidase complex. Hydrogen atoms have been omitted in some cases for clarity.

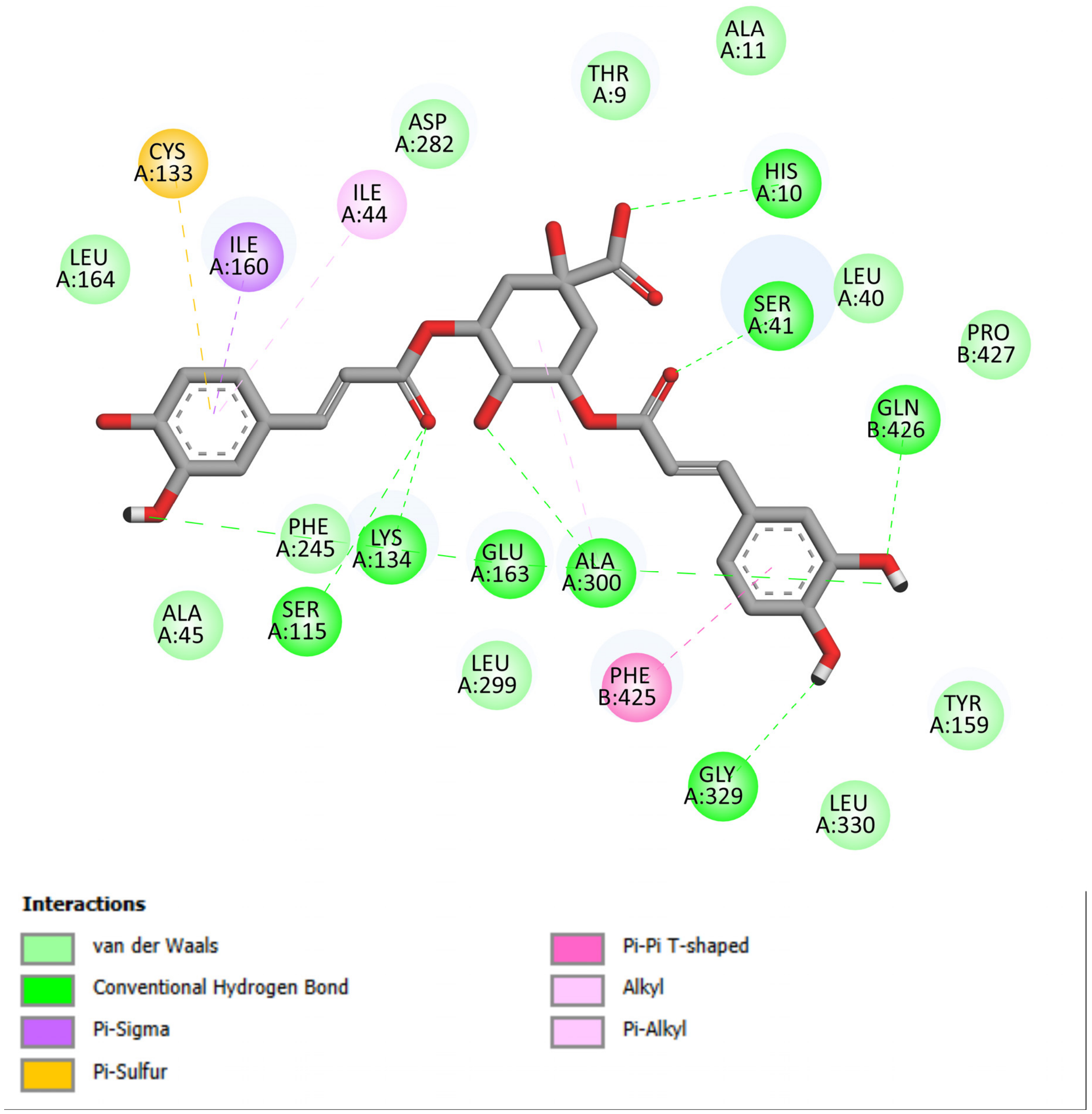

Figure S7. 2D representation of the interactions between Compound **6** – NADPH (PDB ID: 2CDU) oxidase complex. Hydrogen atoms have been omitted in some cases for clarity.

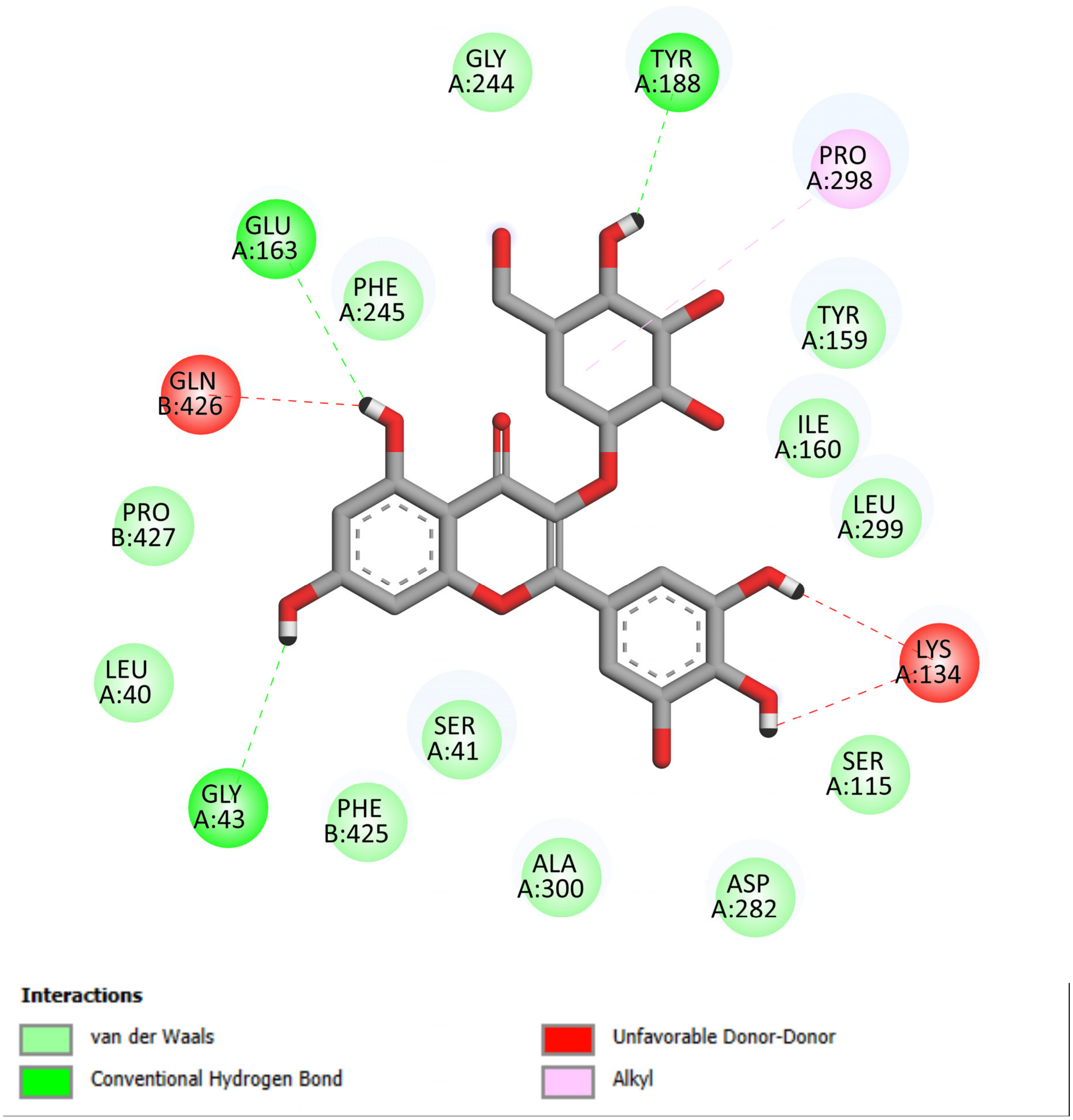

Figure S8. 2D representation of the interactions between Compound **7** – NADPH (PDB ID: 2CDU) oxidase complex. Hydrogen atoms have been omitted in some cases for clarity.

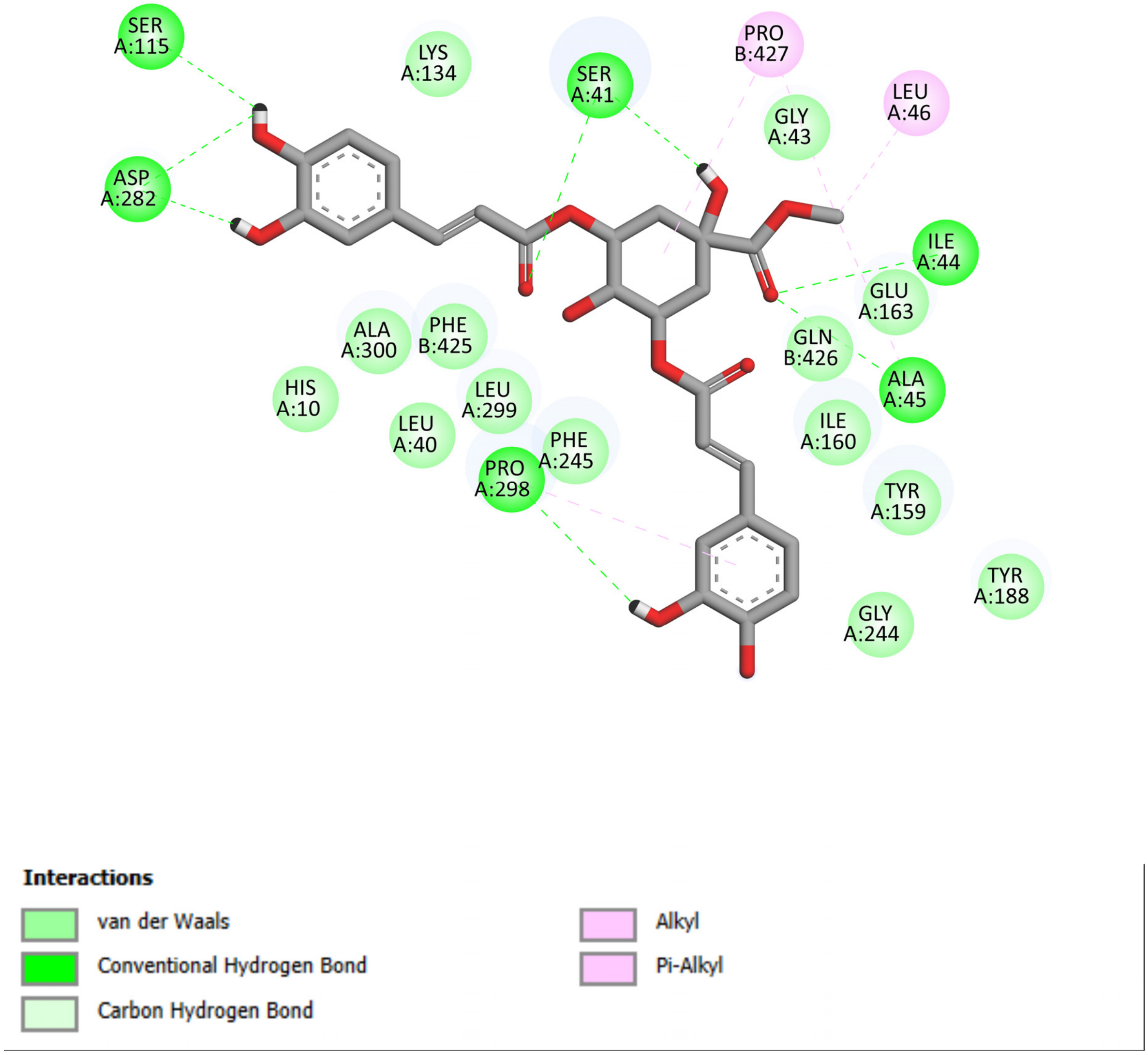

Figure S9. 2D representation of the interactions between Compound **8** – NADPH (PDB ID: 2CDU) oxidase complex. Hydrogen atoms have been omitted in some cases for clarity.

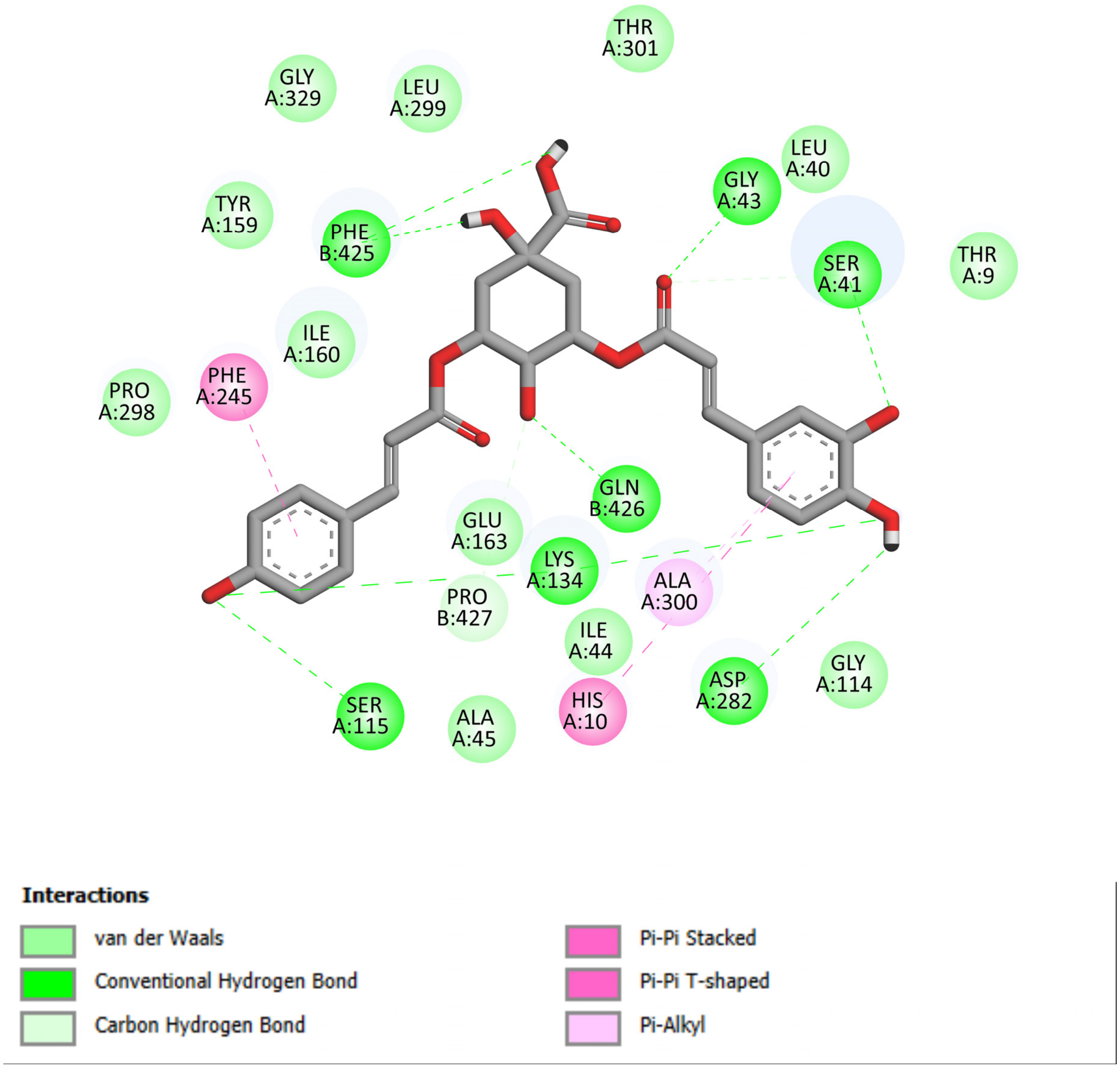

Figure S10. 2D representation of the interactions between Compound **9** – NADPH (PDB ID: 2CDU) oxidase complex. Hydrogen atoms have been omitted in some cases for clarity.

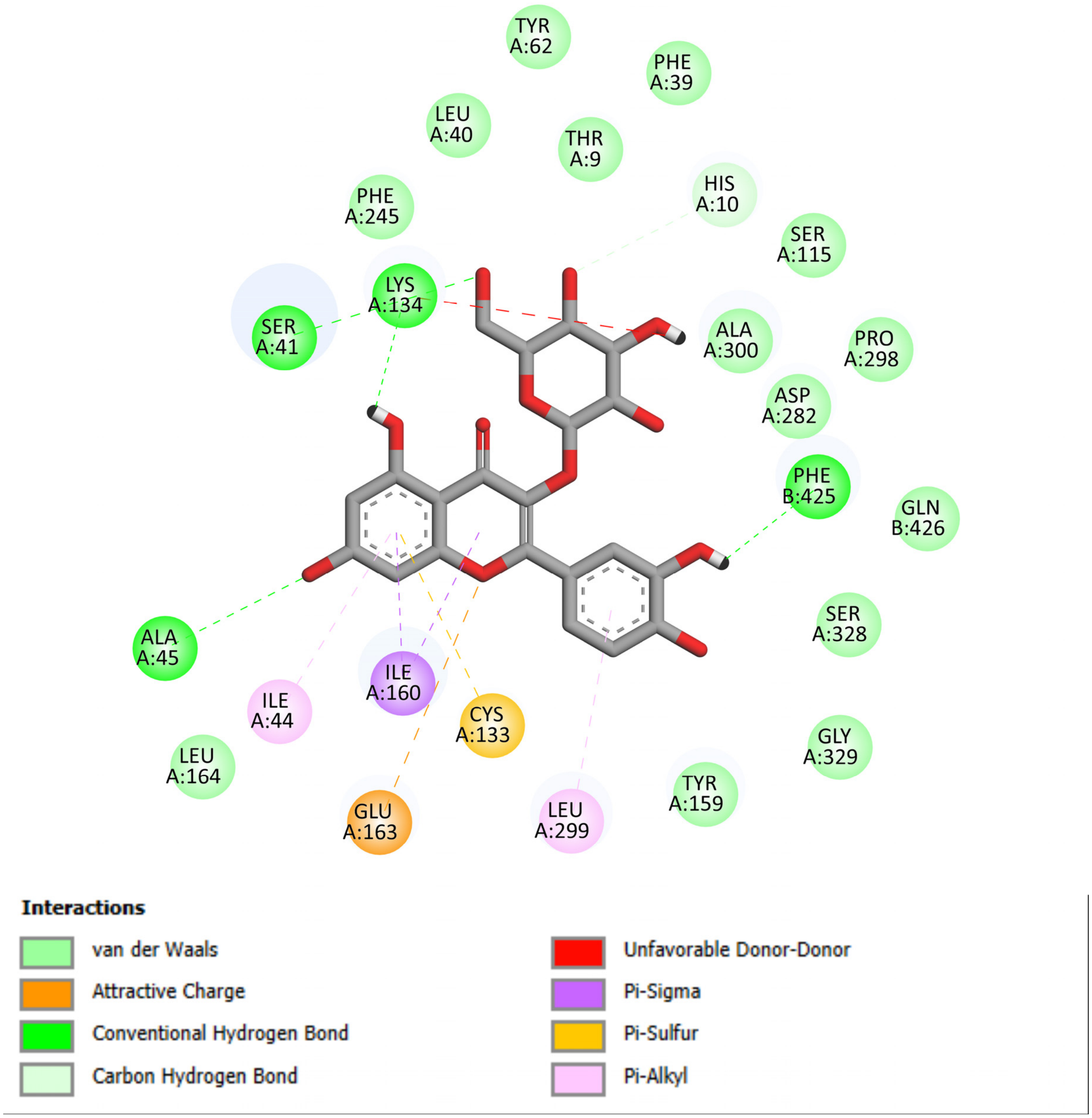

Figure S11. 2D representation of the interactions between compound **10** – NADPH (PDB ID: 2CDU) oxidase complex. Hydrogen atoms have been omitted in some cases for clarity.

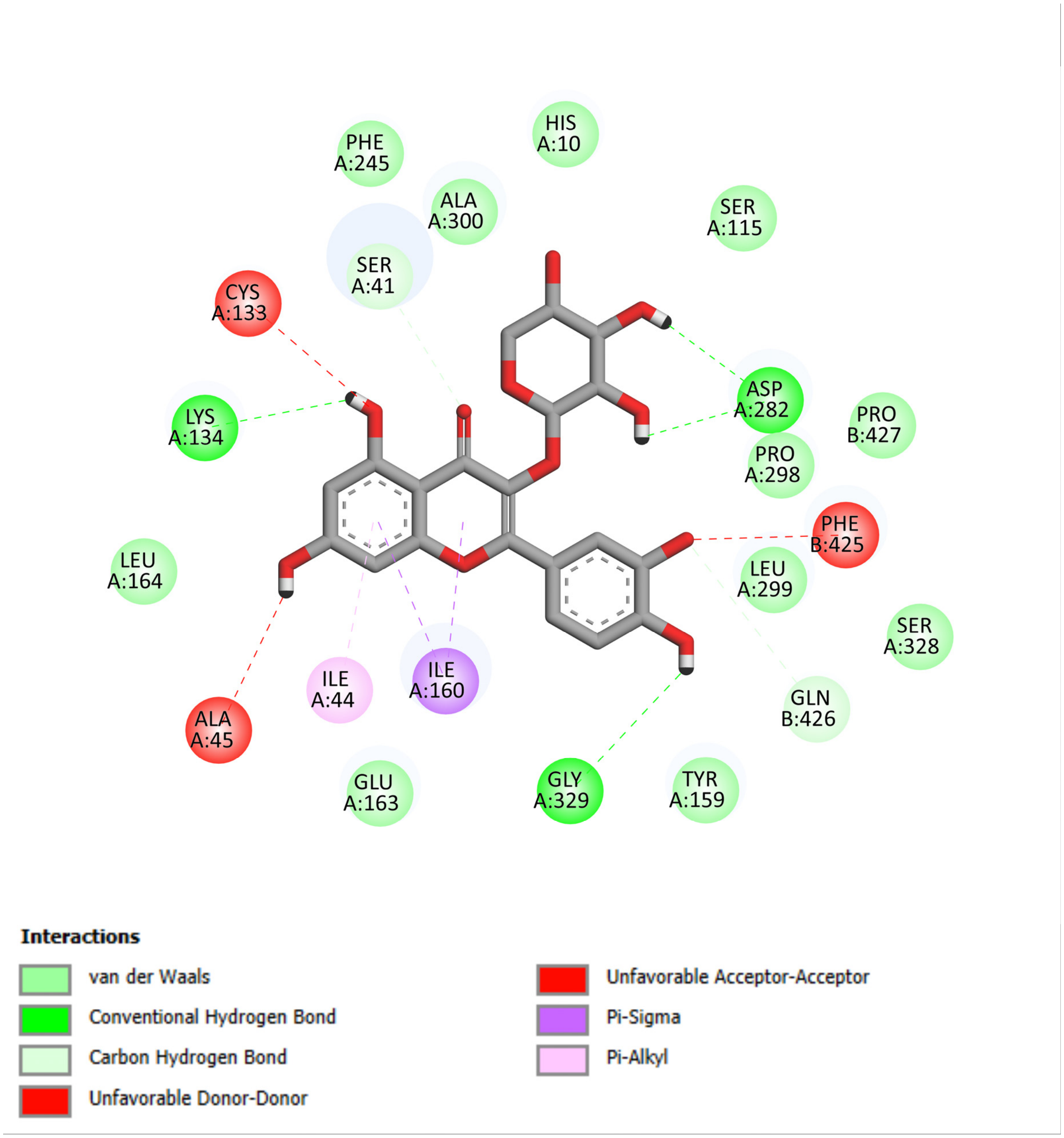

Figure S12. 2D representation of the interactions between compound **1** – Xanthine oxidase (PDB ID: 3NRZ) complex. Hydrogen atoms have been omitted in some cases for clarity.

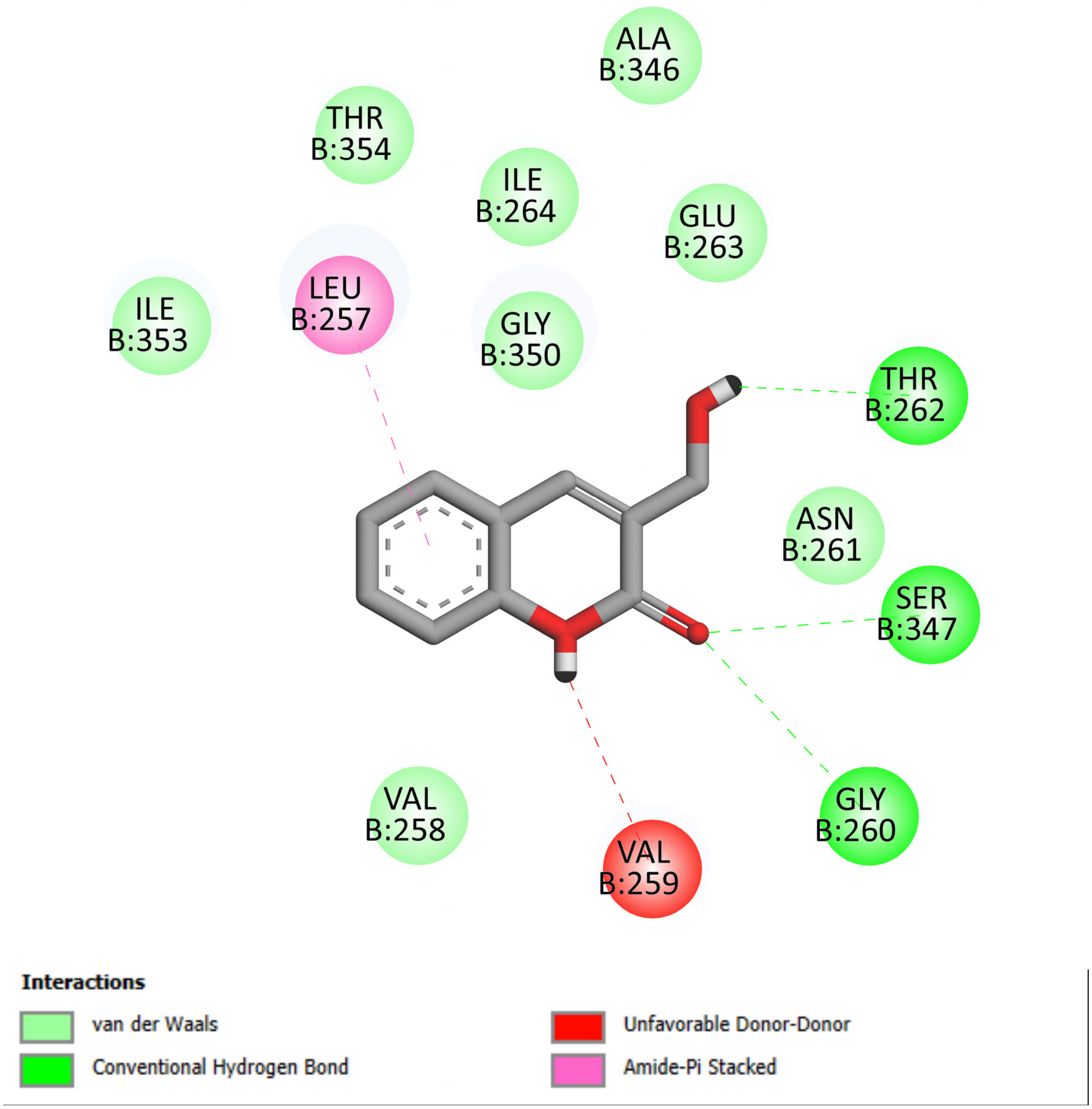

Figure S13. 2D representation of the interactions between compound **2** – Xanthine oxidase (PDB ID: 3NRZ) complex. Hydrogen atoms have been omitted in some cases for clarity.

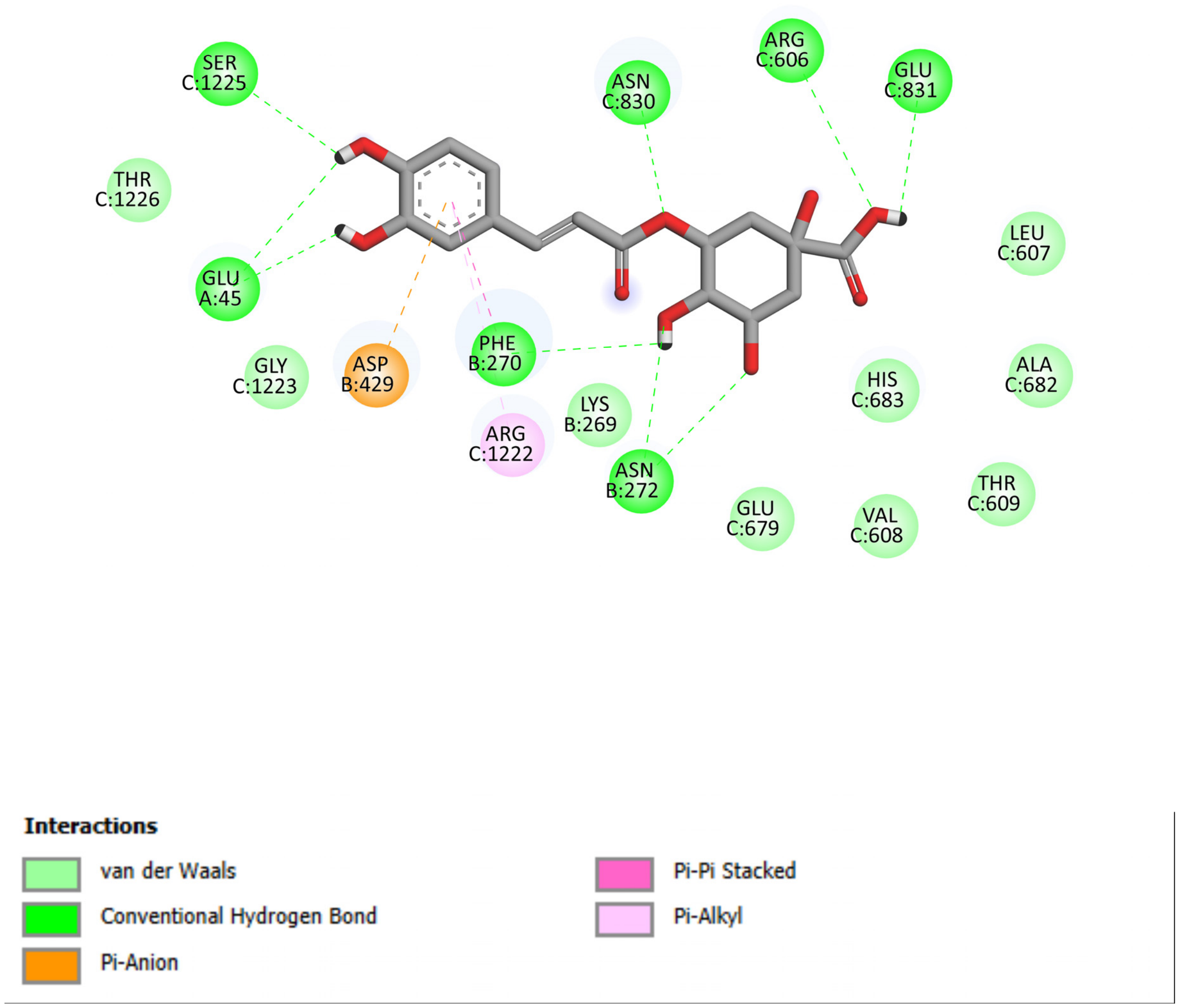

|**Figure S14.** 2D representation of the interactions between compound **3** – Xanthine oxidase (PDB ID: 3NRZ) complex. Hydrogen atoms have been omitted in some cases for clarity.

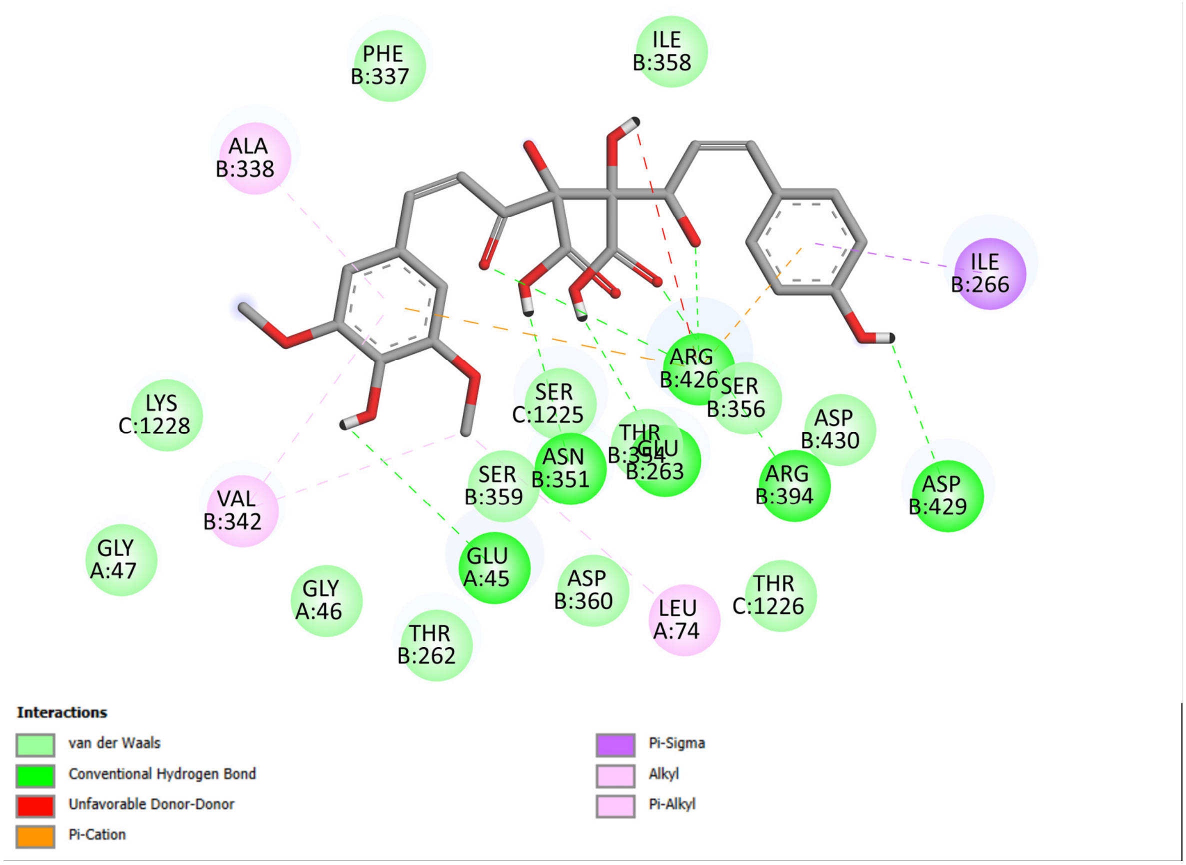

Figure S15. 2D representation of the interactions between compound **4** – Xanthine oxidase (PDB ID: 3NRZ) complex. Hydrogen atoms have been omitted in some cases for clarity.

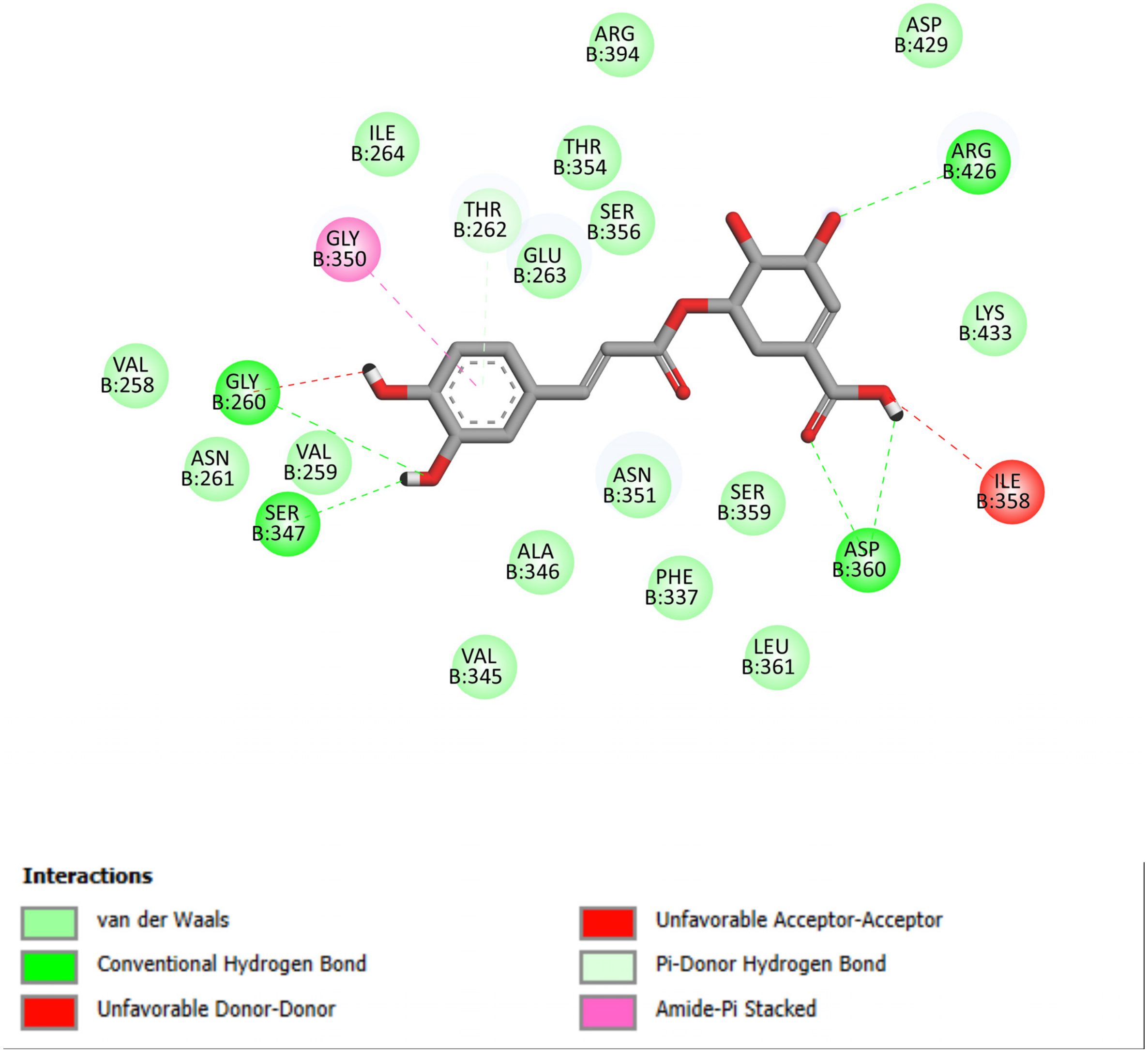

Figure S16. 2D representation of the interactions between compound **5** – Xanthine oxidase (PDB ID: 3NRZ) complex. Hydrogen atoms have been omitted in some cases for clarity.

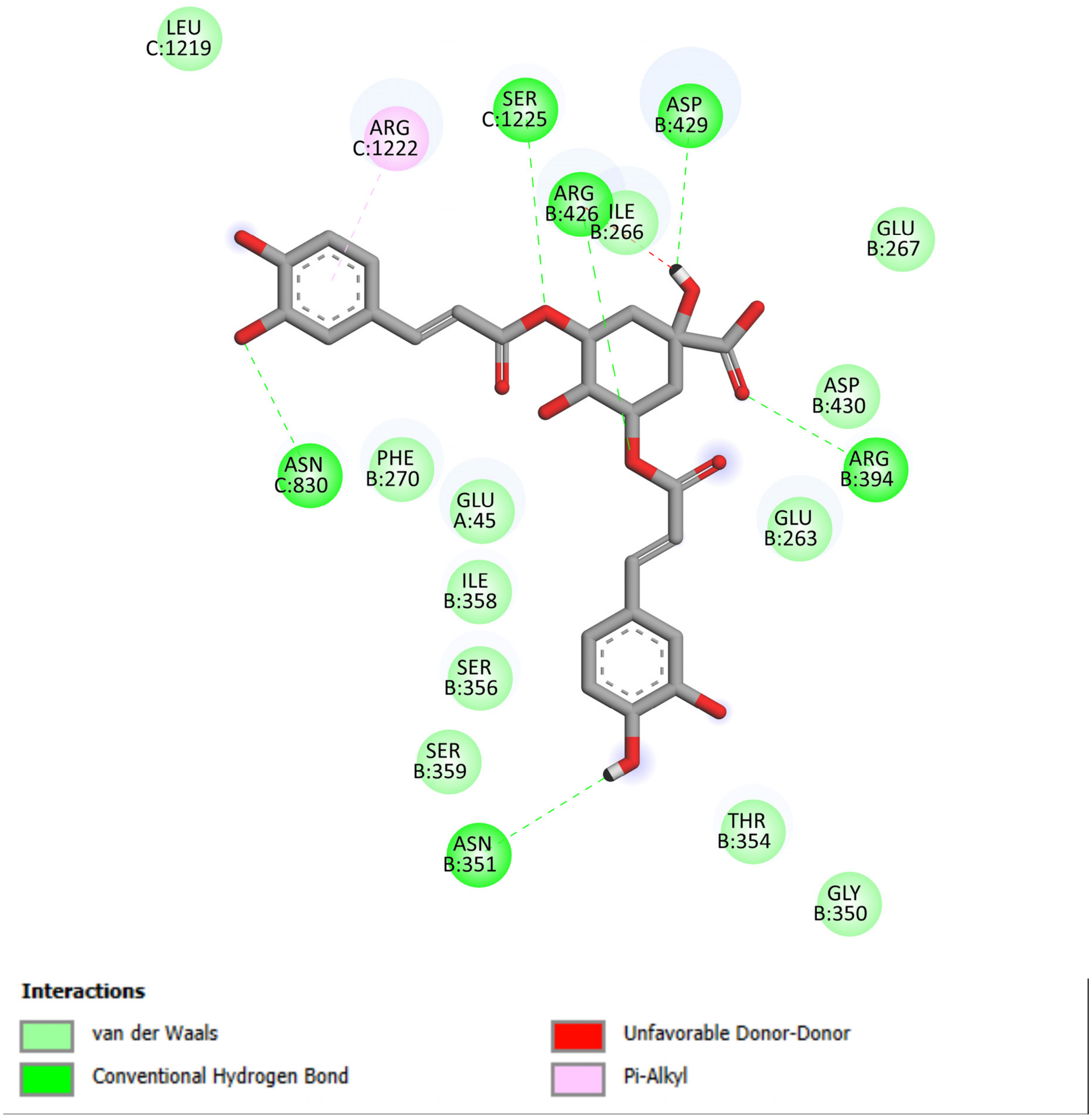

Figure S17. 2D representation of the interactions between compound **6** – Xanthine oxidase (PDB ID: 3NRZ) complex. Hydrogen atoms have been omitted in some cases for clarity.

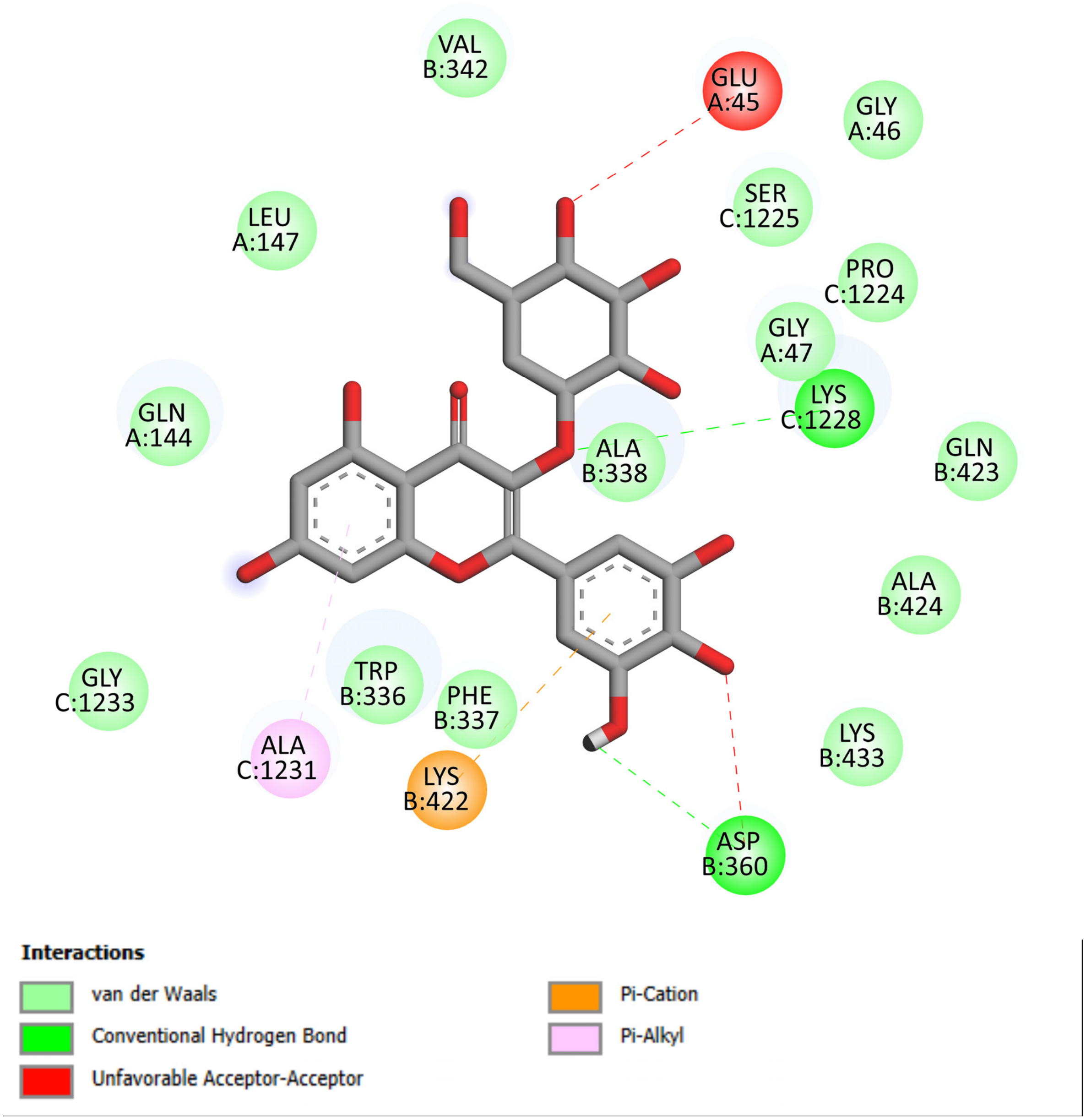

Figure S18. 2D representation of the interactions between compound **7** – Xanthine oxidase (PDB ID: 3NRZ) complex. Hydrogen atoms have been omitted in some cases for clarity.

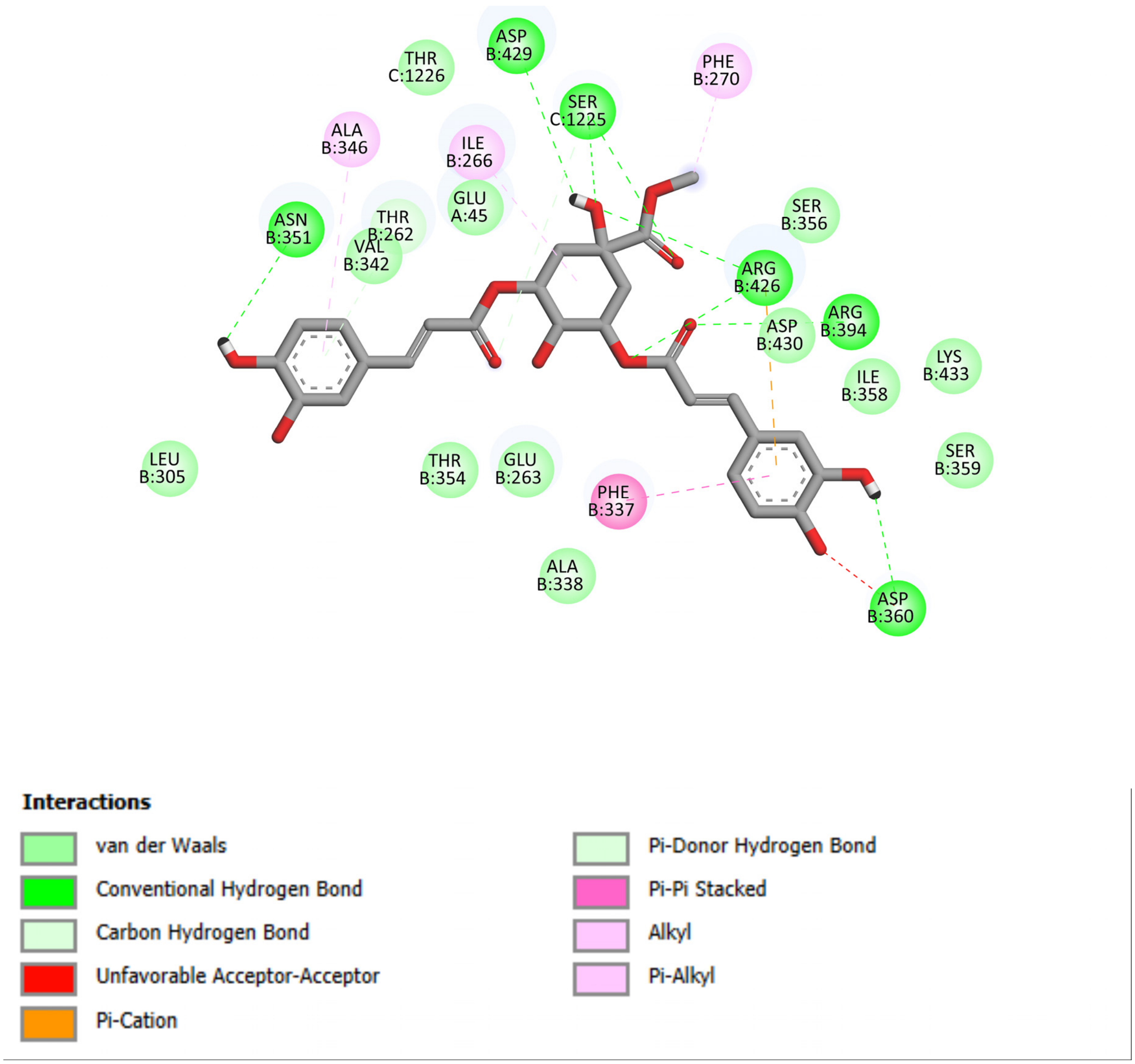

Figure S19. 2D representation of the interactions between compound **8** – Xanthine oxidase (PDB ID: 3NRZ) complex. Hydrogen atoms have been omitted in some cases for clarity.

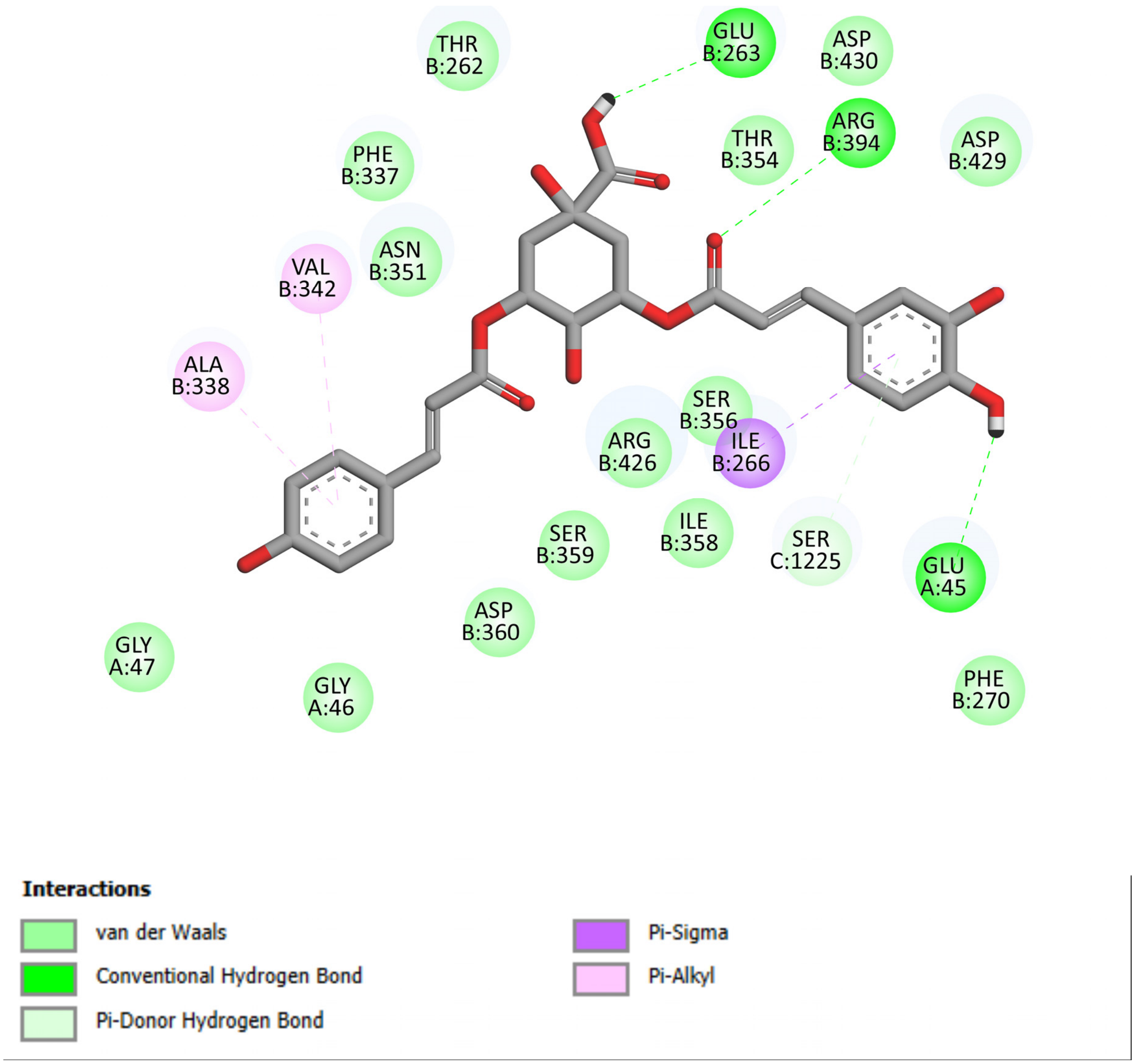

Figure S20. 2D representation of the interactions between compound **9** – Xanthine oxidase (PDB ID: 3NRZ) complex. Hydrogen atoms have been omitted in some cases for clarity.

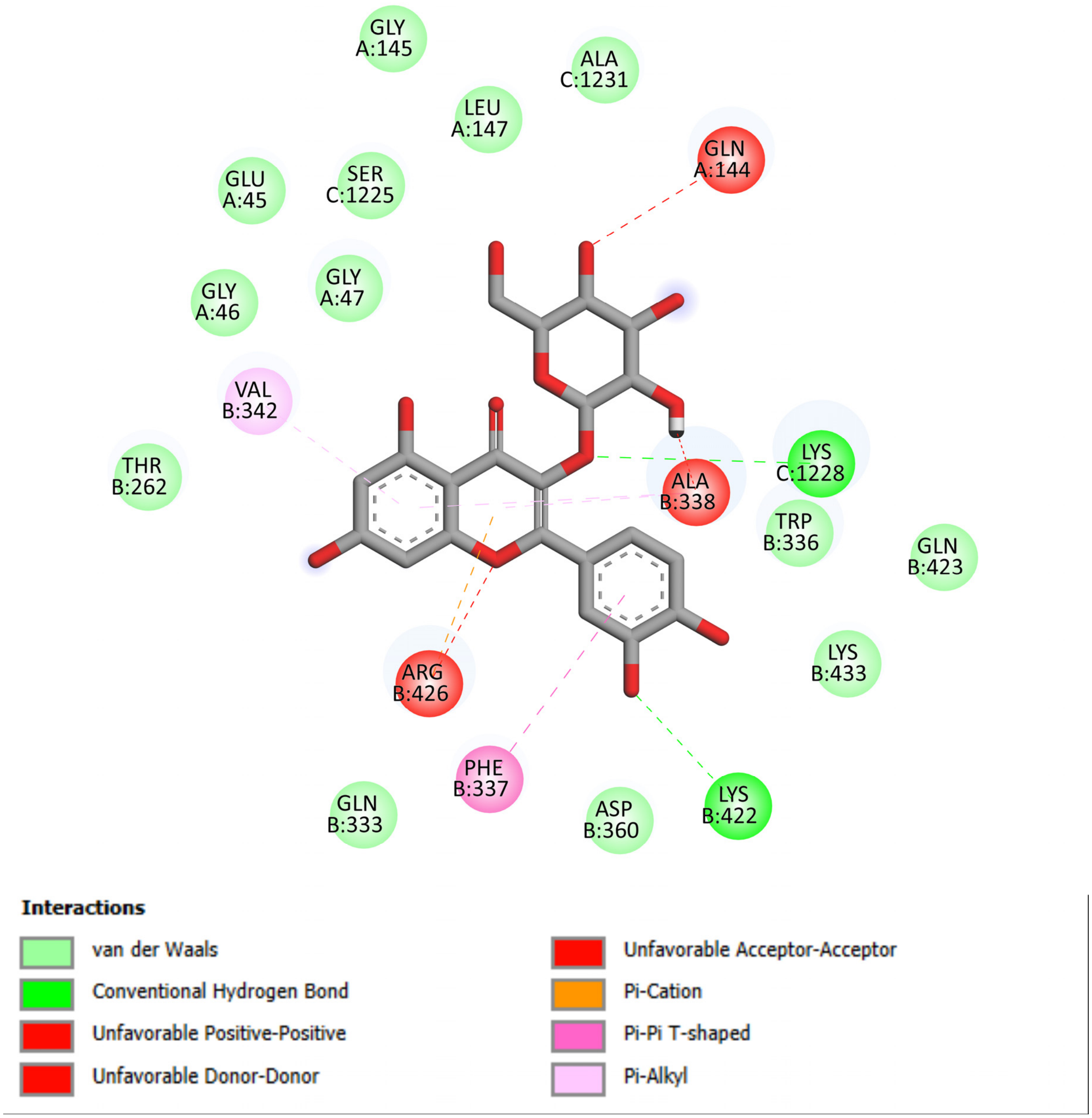

Figure S21. 2D representation of the interactions between compound **10** – Xanthine oxidase (PDB ID: 3NRZ) complex. Hydrogen atoms have been omitted in some cases for clarity.

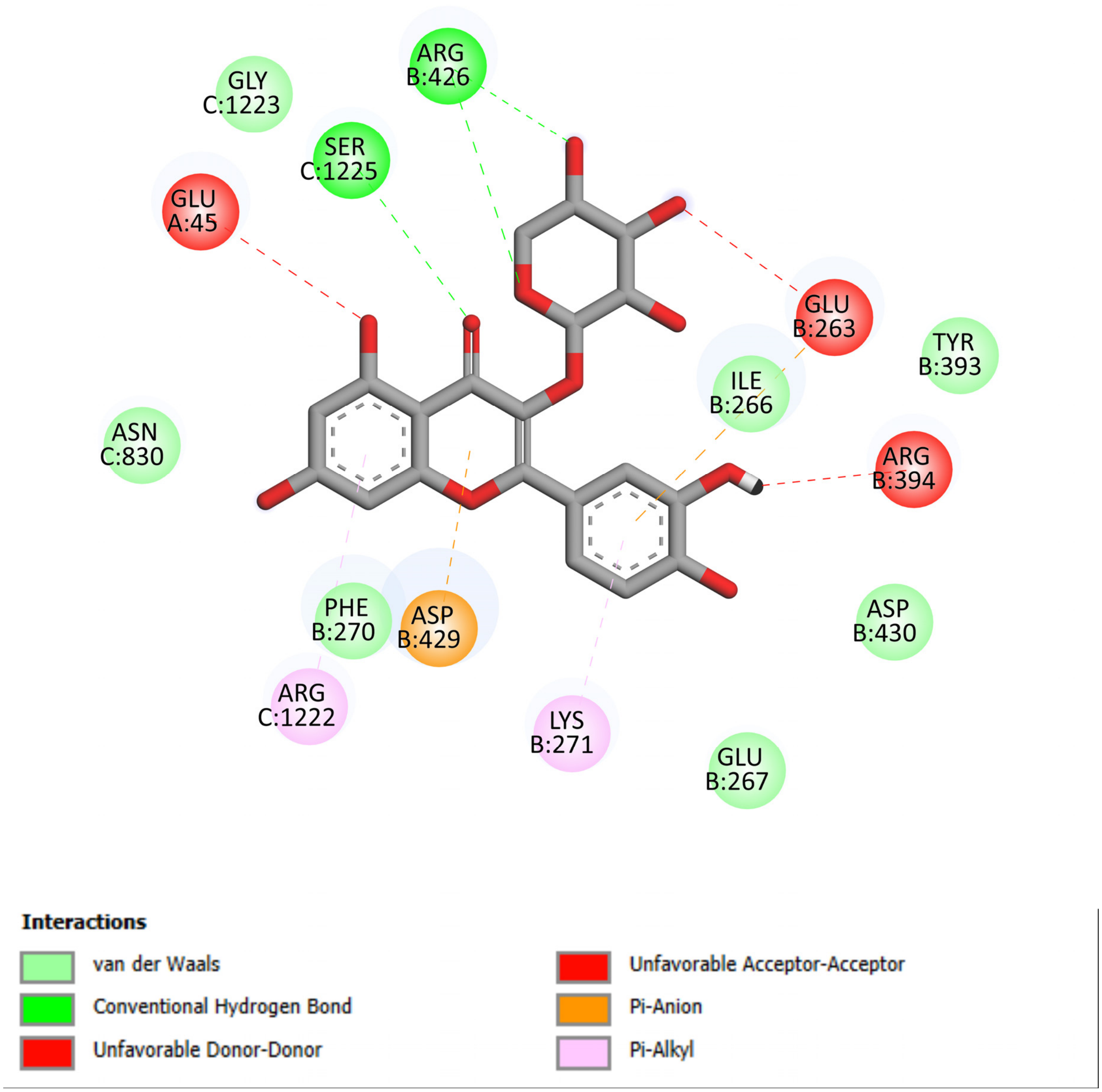

Figure S22. 2D representation of the interactions between compound **11** – Xanthine oxidase (PDB ID: 3NRZ) complex. Hydrogen atoms have been omitted in some cases for clarity.

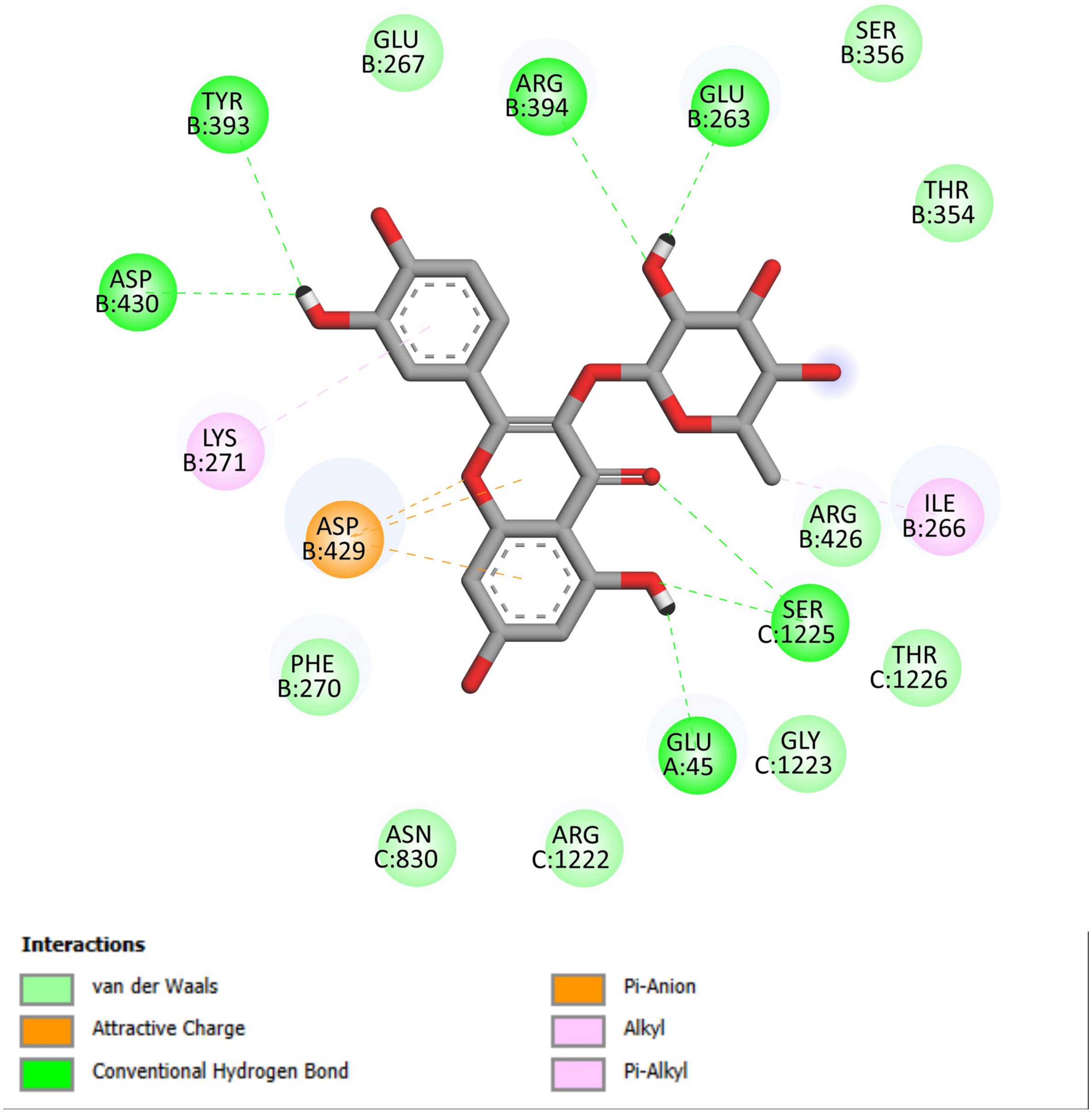

Figure S23. 2D representation of the interactions between compound **1** – Superoxide dismutase (PDB ID: 4MCM) complex. Hydrogen atoms have been omitted in some cases for clarity.

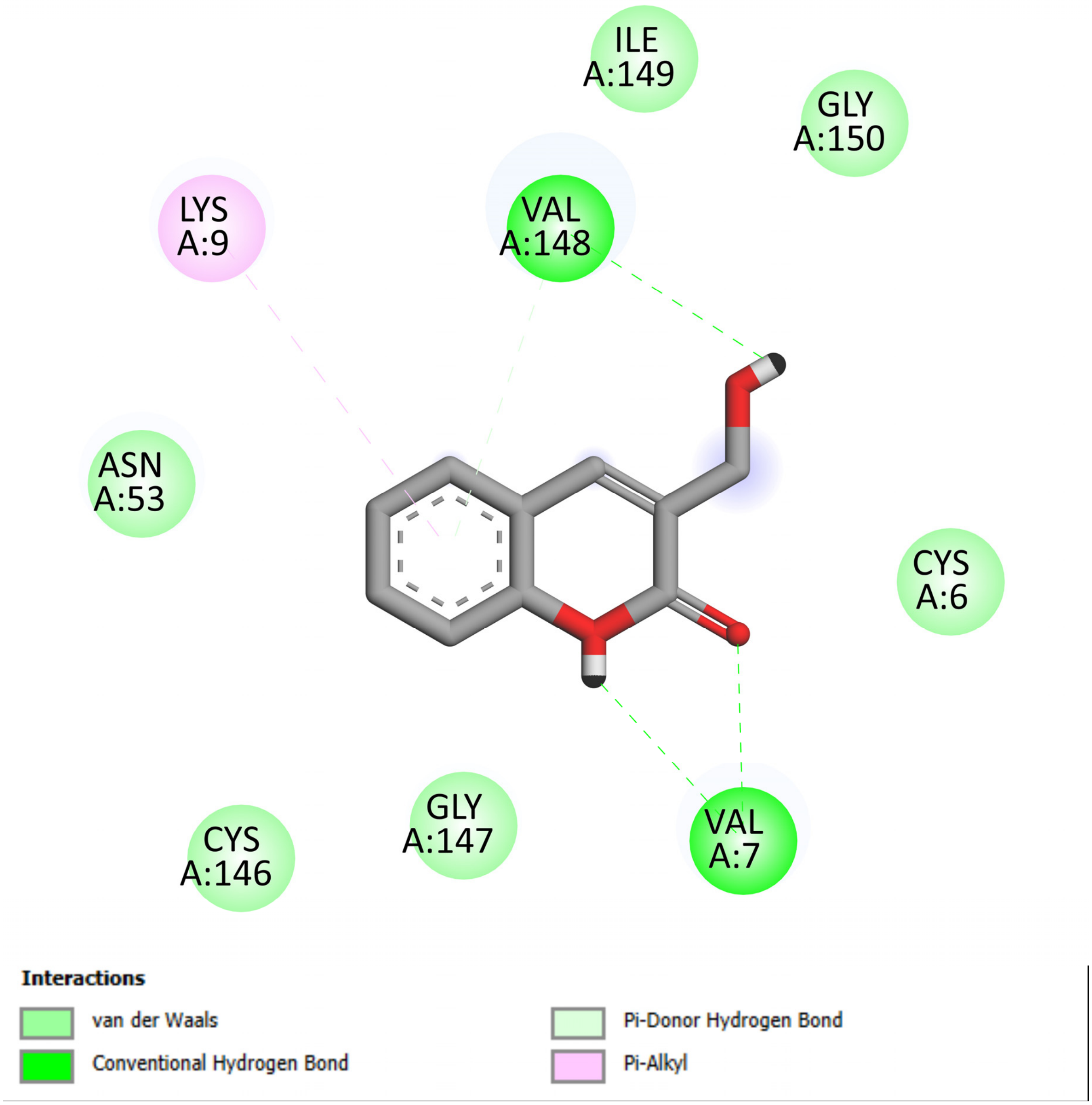

Figure S24. 2D representation of the interactions between compound **2** – Superoxide dismutase (PDB ID: 4MCM) complex. Hydrogen atoms have been omitted in some cases for clarity.

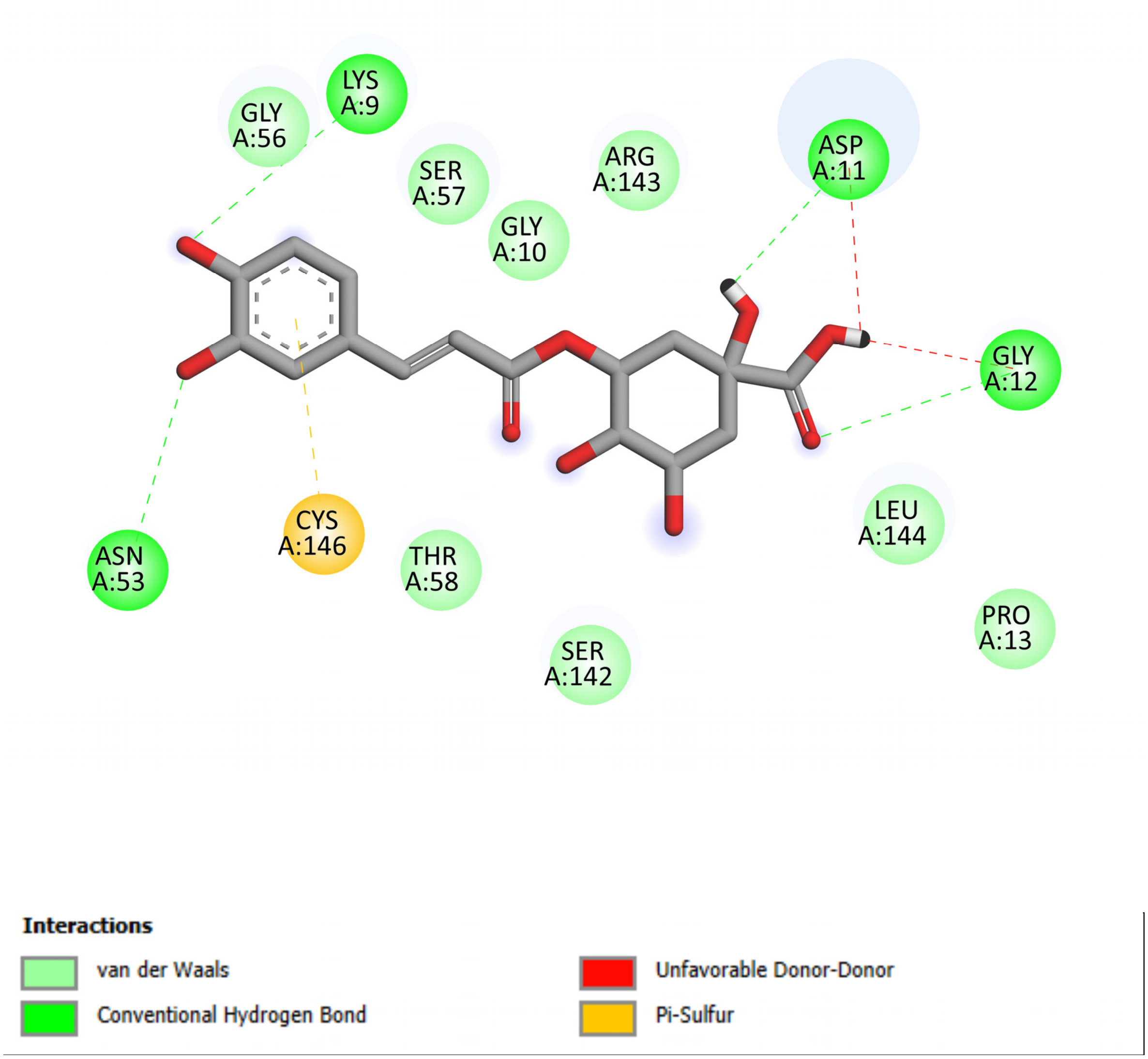

Figure S25. 2D representation of the interactions between compound **3** – Superoxide dismutase (PDB ID: 4MCM) complex. Hydrogen atoms have been omitted in some cases for clarity.

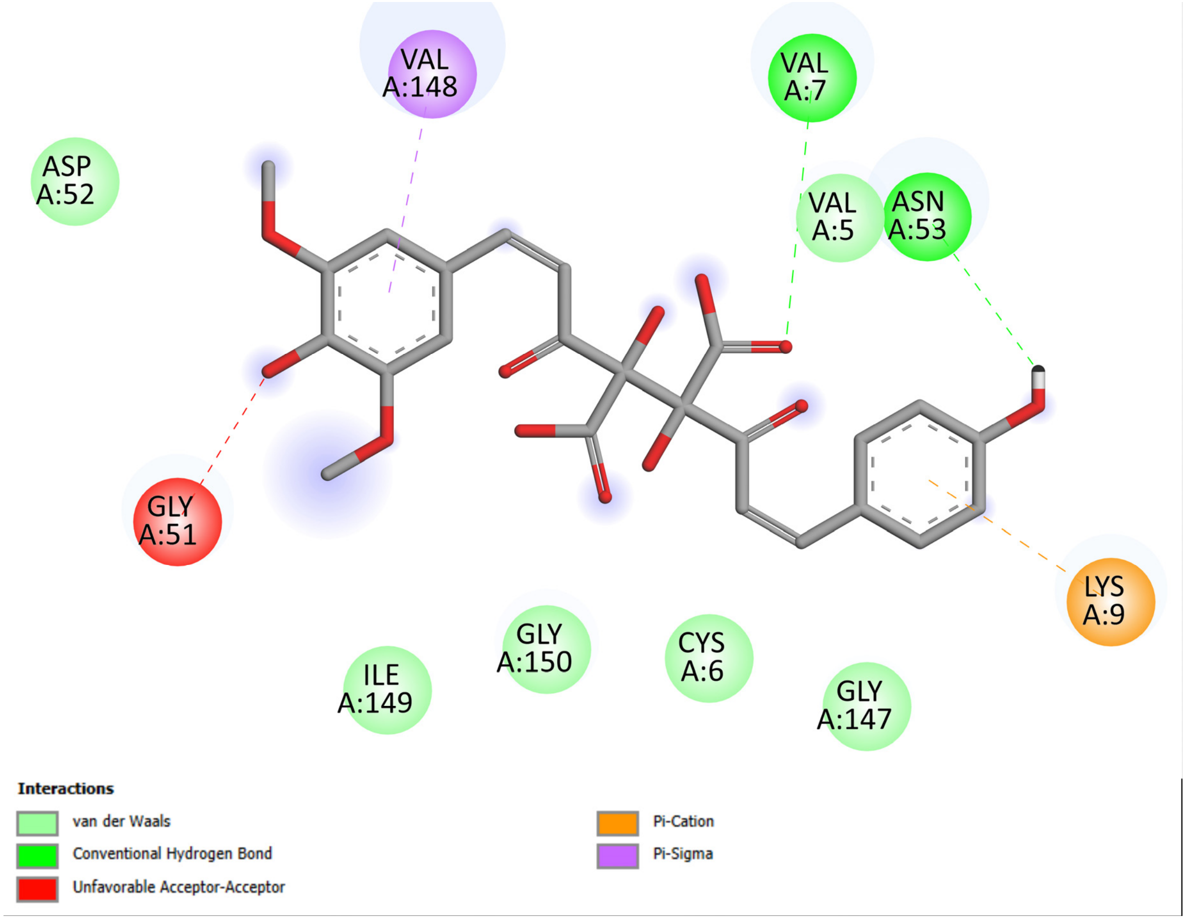

Figure S26. 2D representation of the interactions between compound **4** – Superoxide dismutase (PDB ID: 4MCM) complex. Hydrogen atoms have been omitted in some cases for clarity.

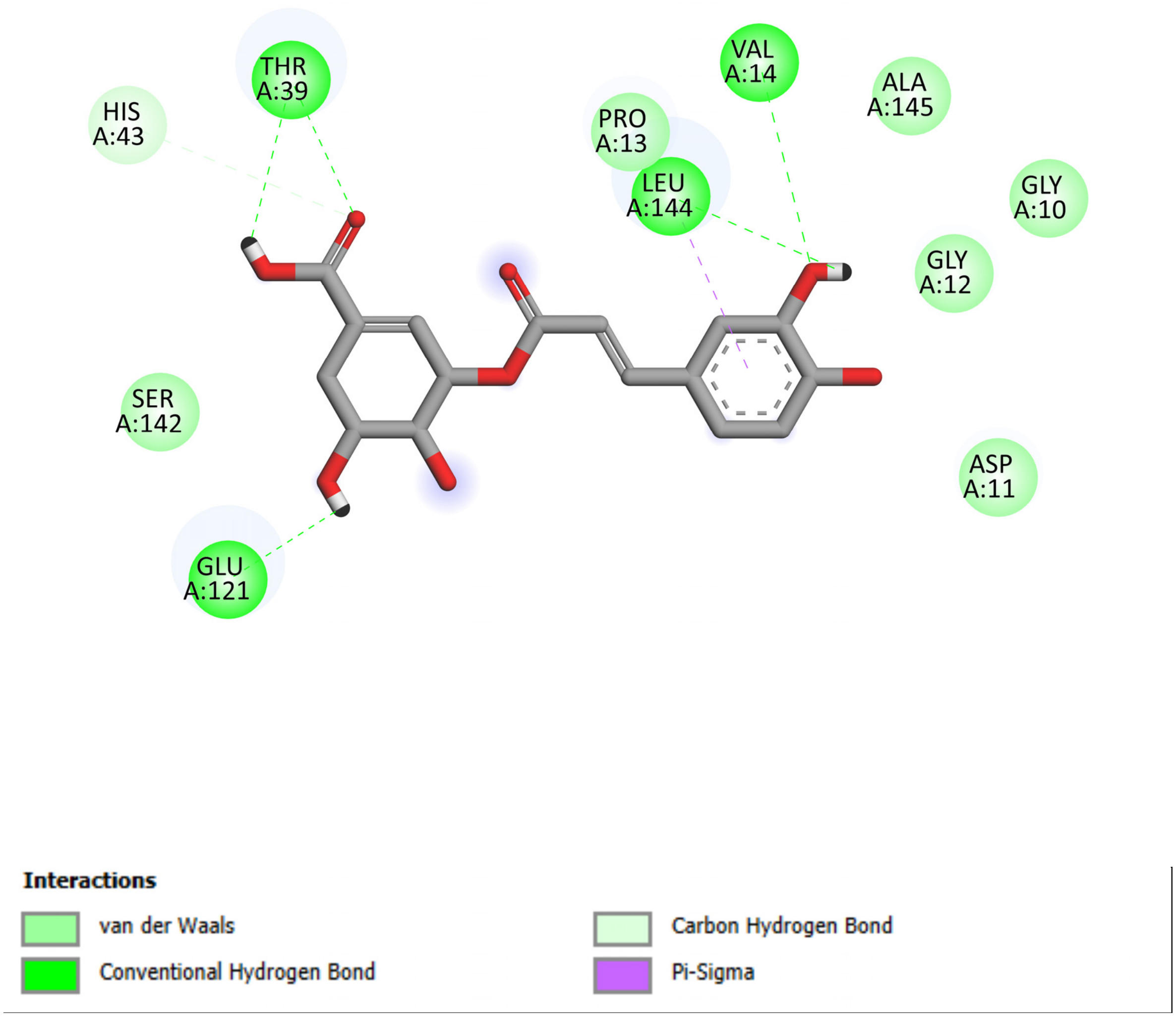

Figure S27. 2D representation of the interactions between compound **5** – Superoxide dismutase (PDB ID: 4MCM) complex. Hydrogen atoms have been omitted in some cases for clarity.

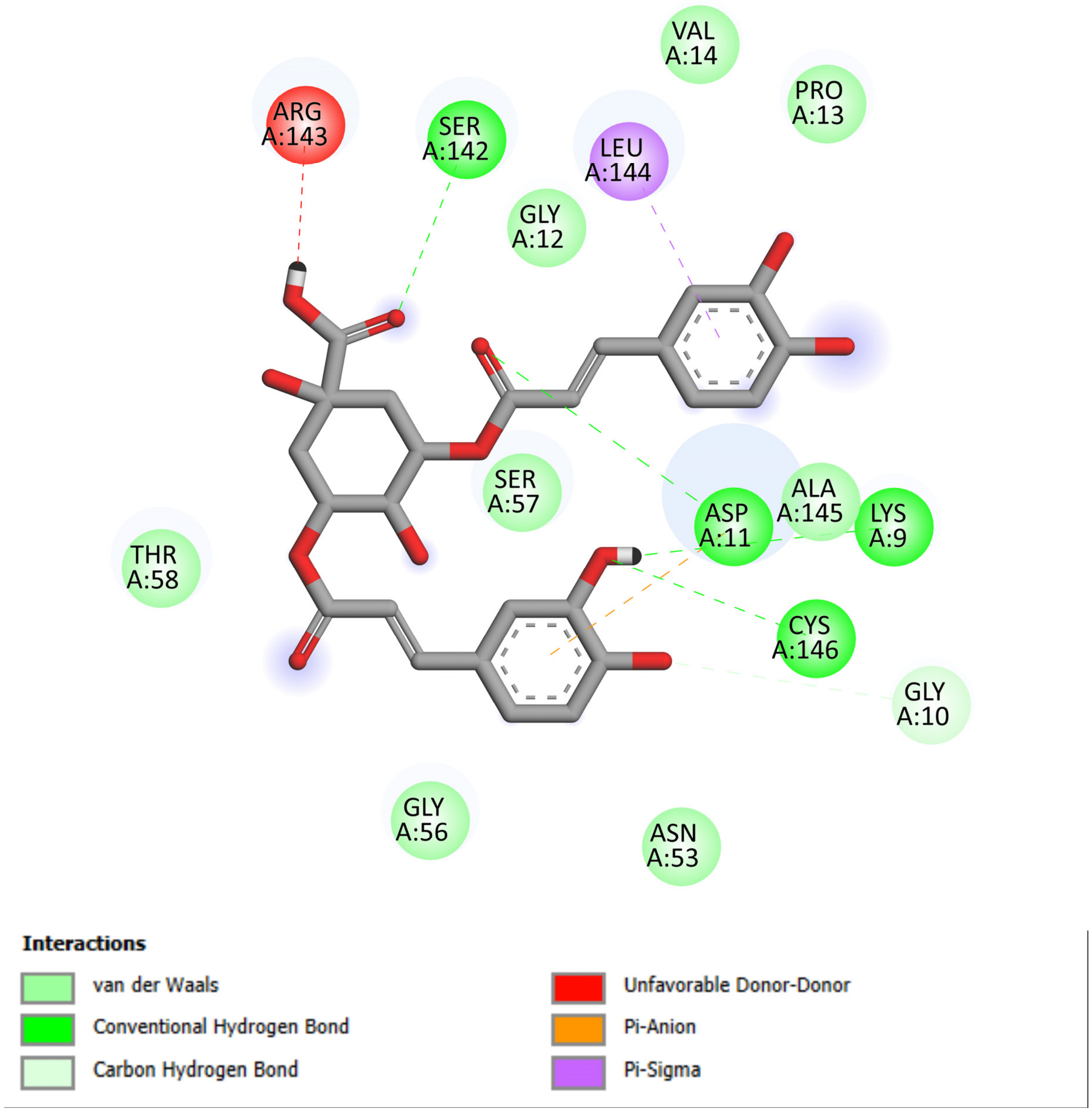

Figure S28. 2D representation of the interactions between compound **6** – Superoxide dismutase (PDB ID: 4MCM) complex. Hydrogen atoms have been omitted in some cases for clarity.

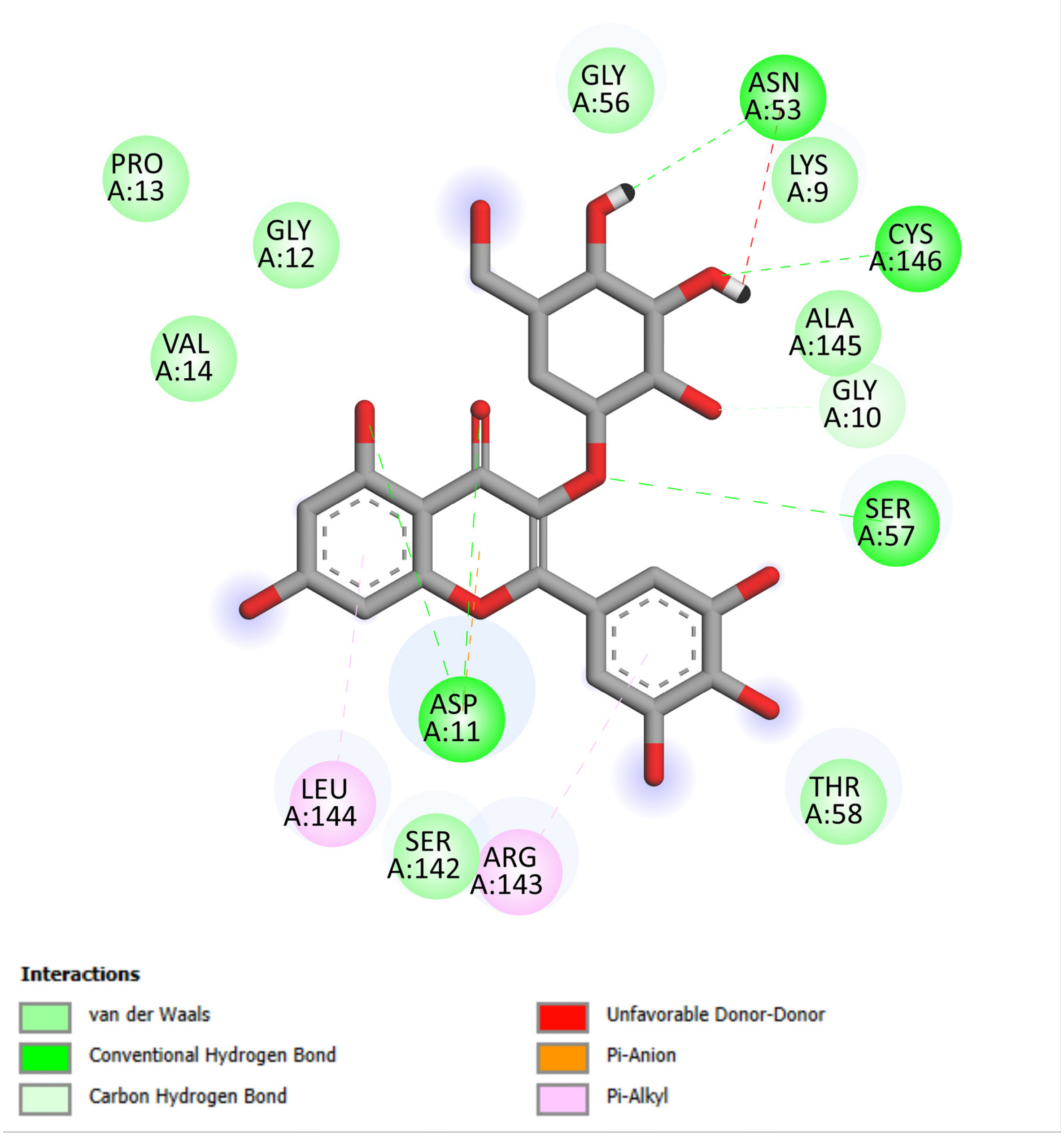

Figure S29. 2D representation of the interactions between compound **7** – Superoxide dismutase (PDB ID: 4MCM) complex. Hydrogen atoms have been omitted in some cases for clarity.

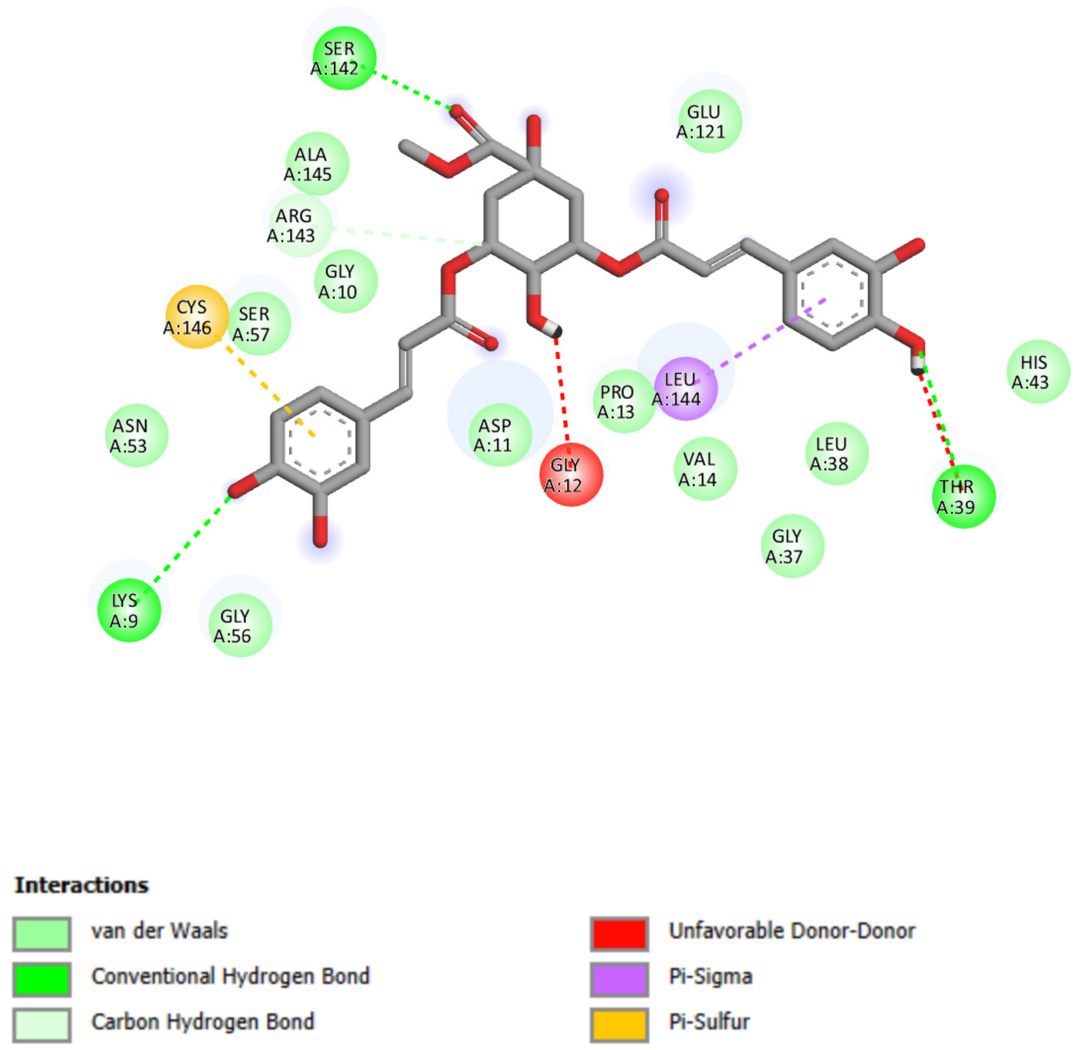

Figure S30. 2D representation of the interactions between compound **8** – Superoxide dismutase (PDB ID: 4MCM) complex. Hydrogen atoms have been omitted in some cases for clarity.

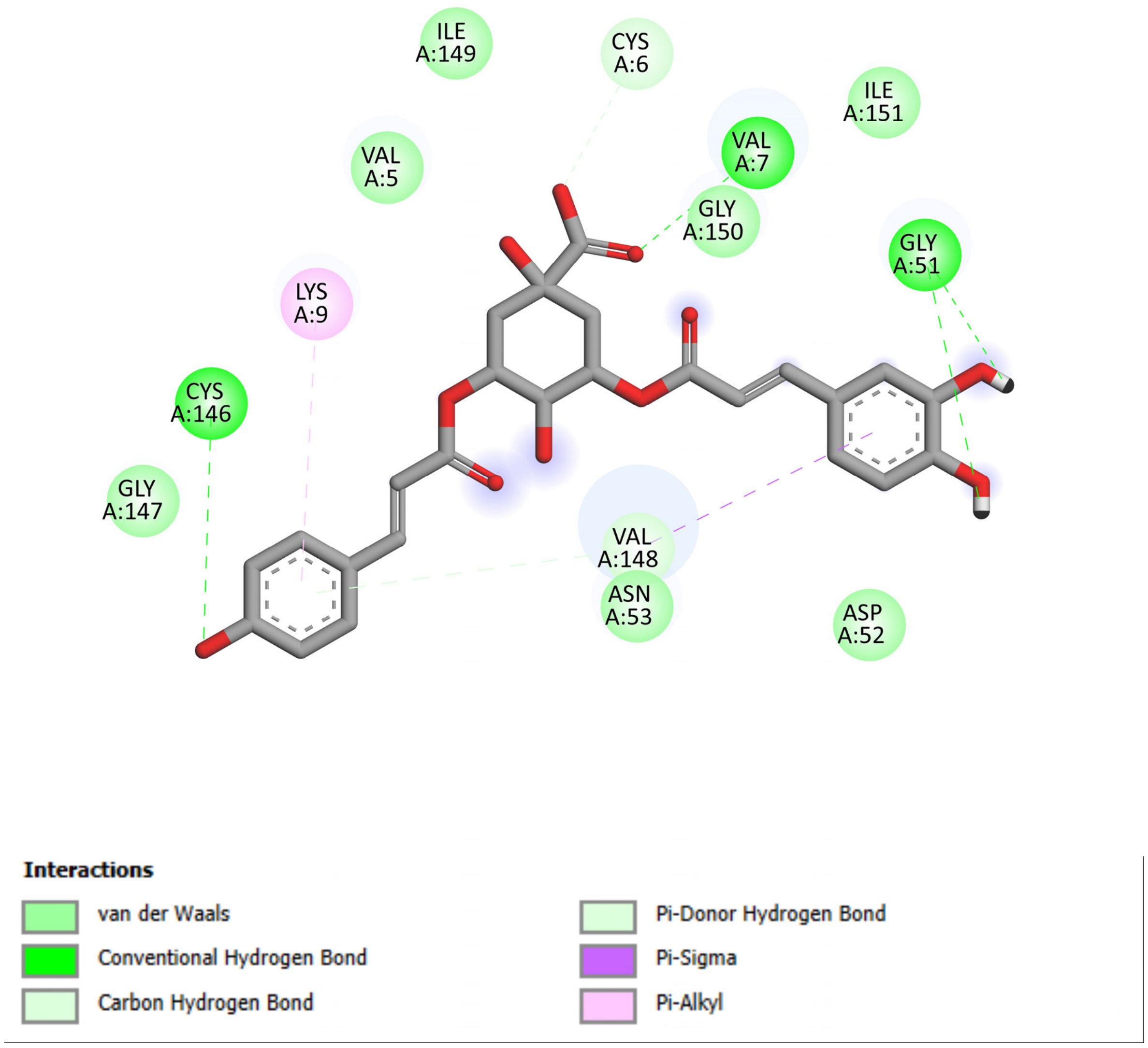

Figure S31. 2D representation of the interactions between compound **9** – Superoxide dismutase (PDB ID: 4MCM) complex. Hydrogen atoms have been omitted in some cases for clarity.

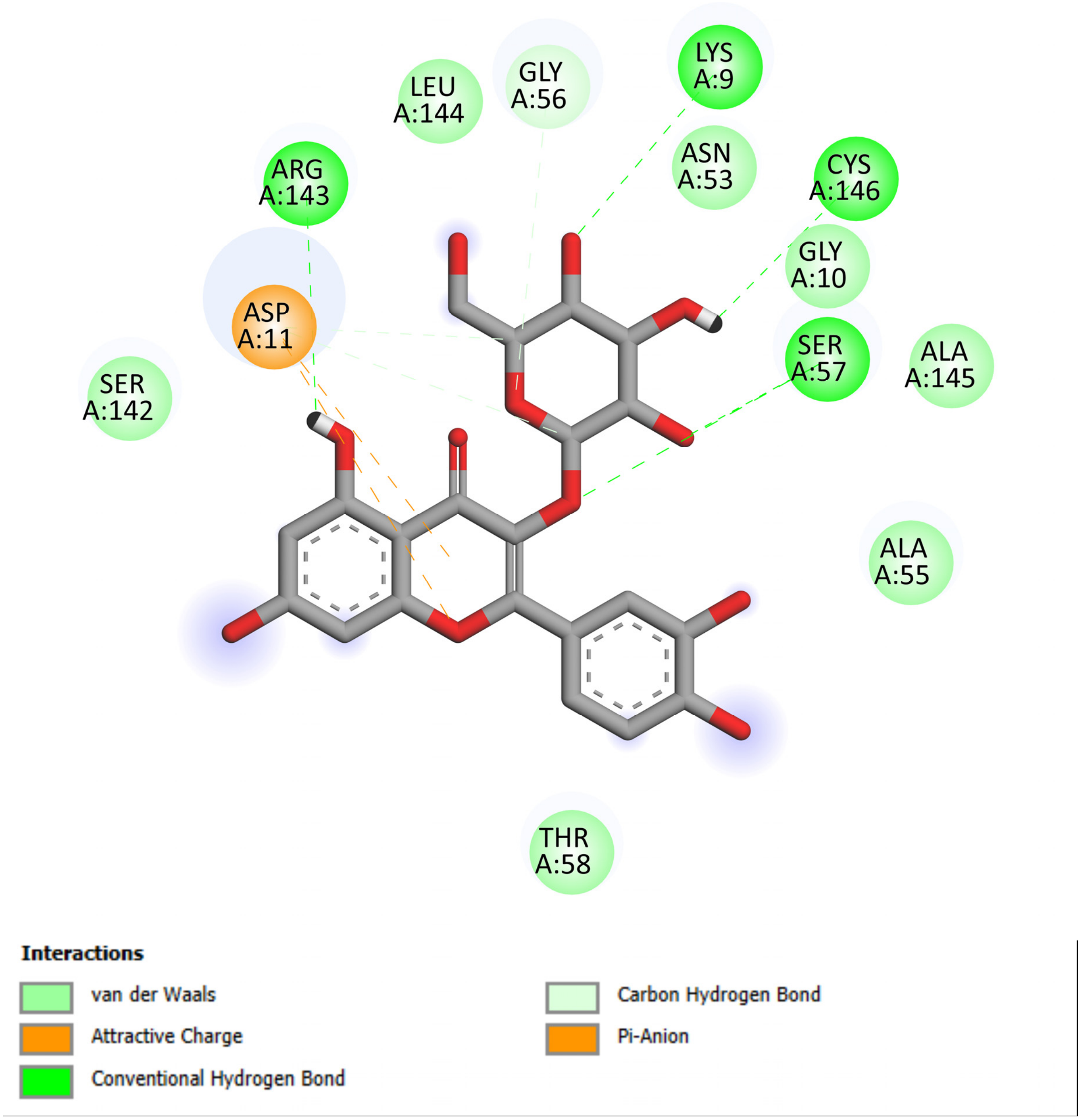

Figure S32. 2D representation of the interactions between compound **10** – Superoxide dismutase (PDB ID: 4MCM) complex. Hydrogen atoms have been omitted in some cases for clarity.

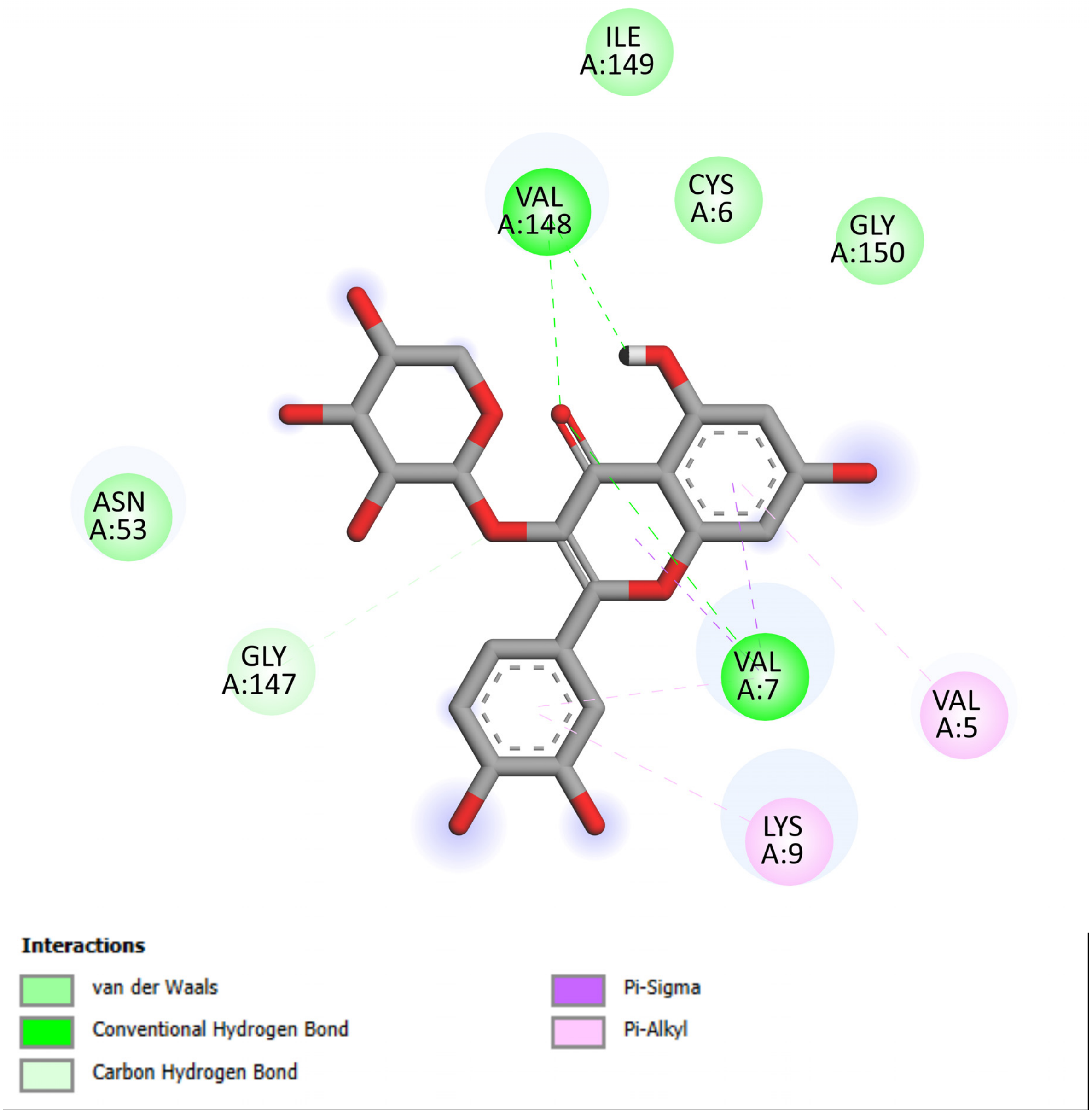

Figure S33. 2D representation of the interactions between compound **10** – Superoxide dismutase (PDB ID: 4MCM) complex. Hydrogen atoms have been omitted in some cases for clarity.

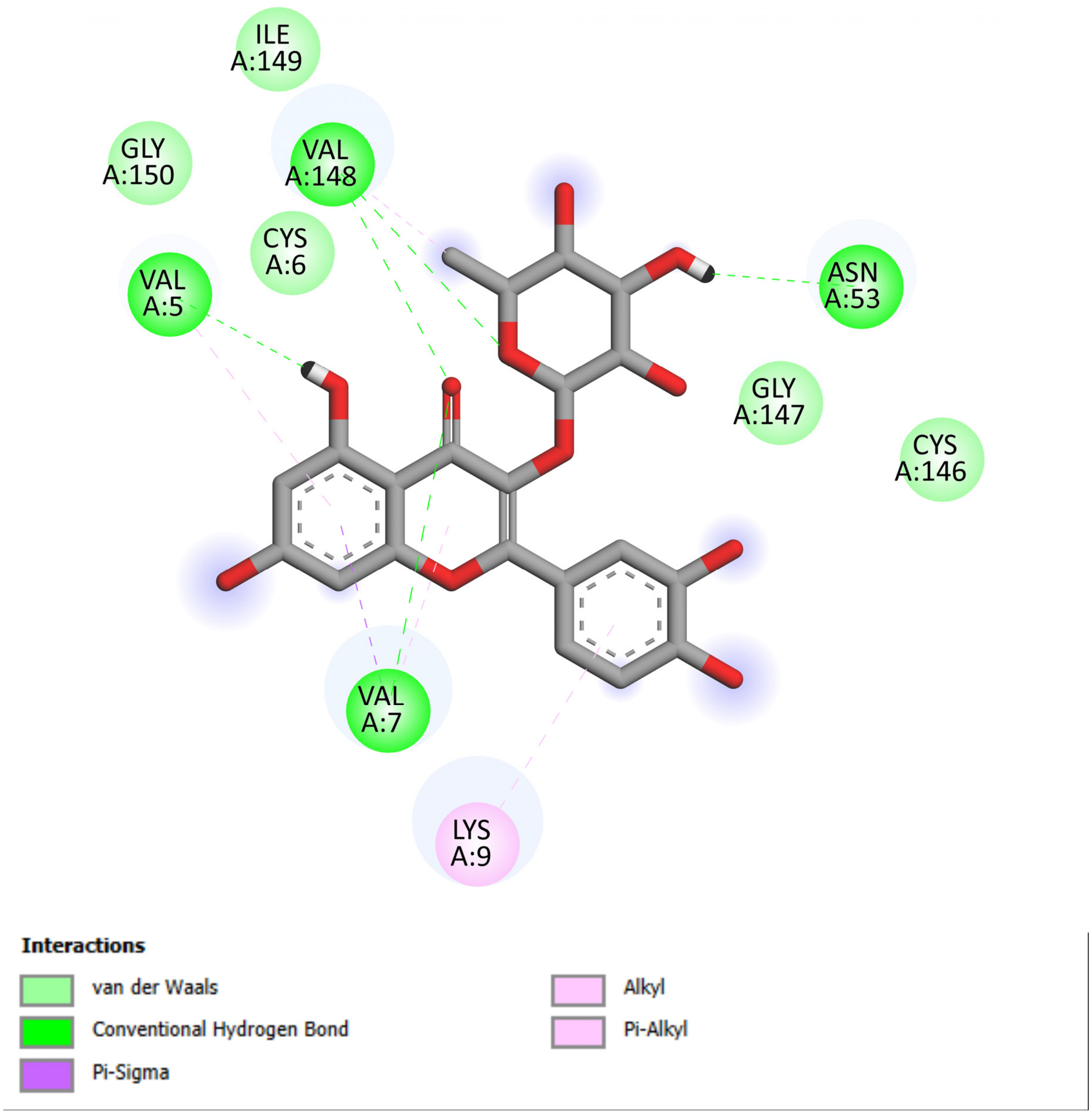

Figure S34. 2D representation of the interactions between compound **1** – ATPase (PDB ID: 1MO7) complex. Hydrogen atoms have been omitted in some cases for clarity.

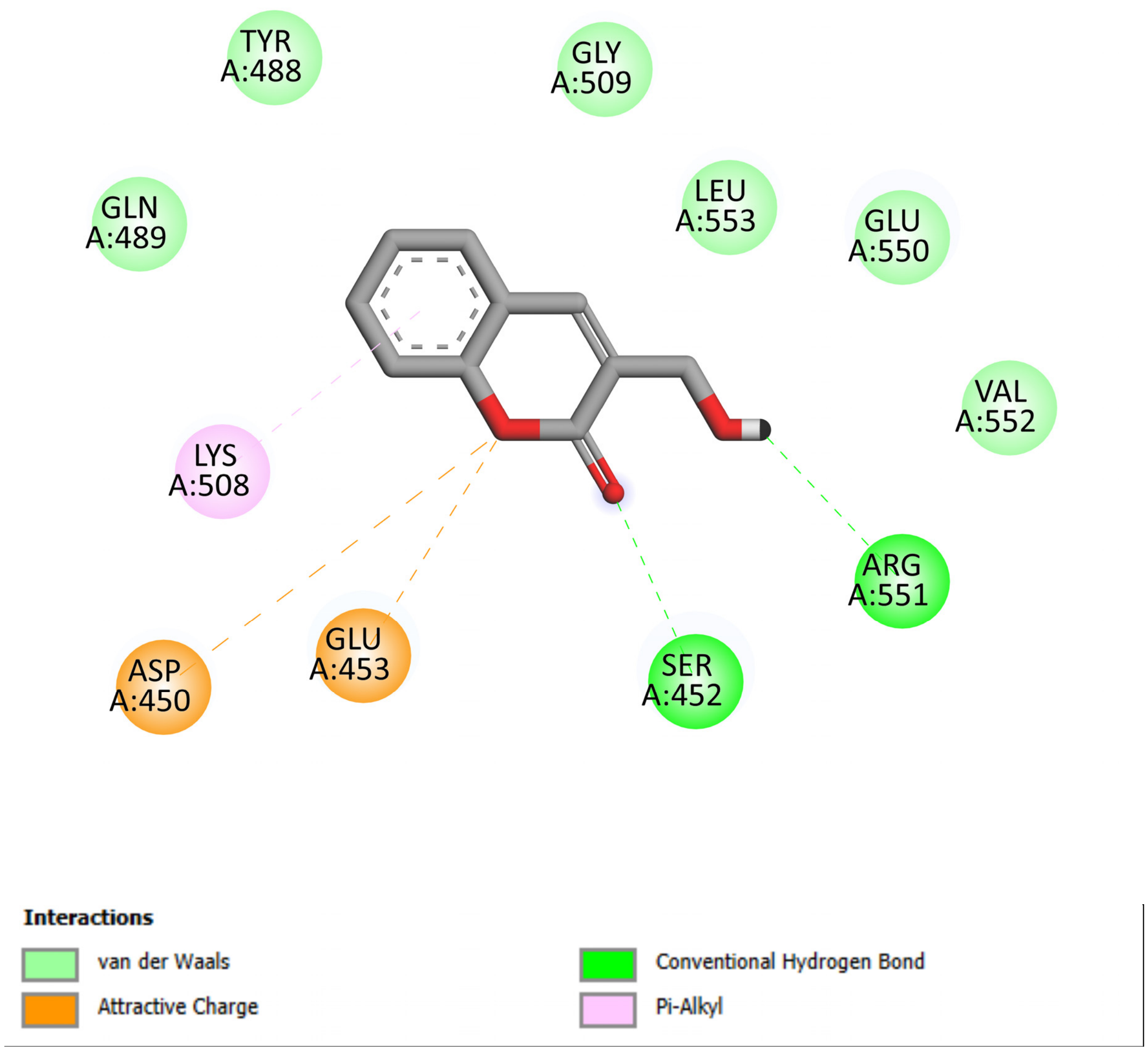

Figure S35. 2D representation of the interactions between compound **2** – ATPase (PDB ID: 1MO7) complex. Hydrogen atoms have been omitted in some cases for clarity.

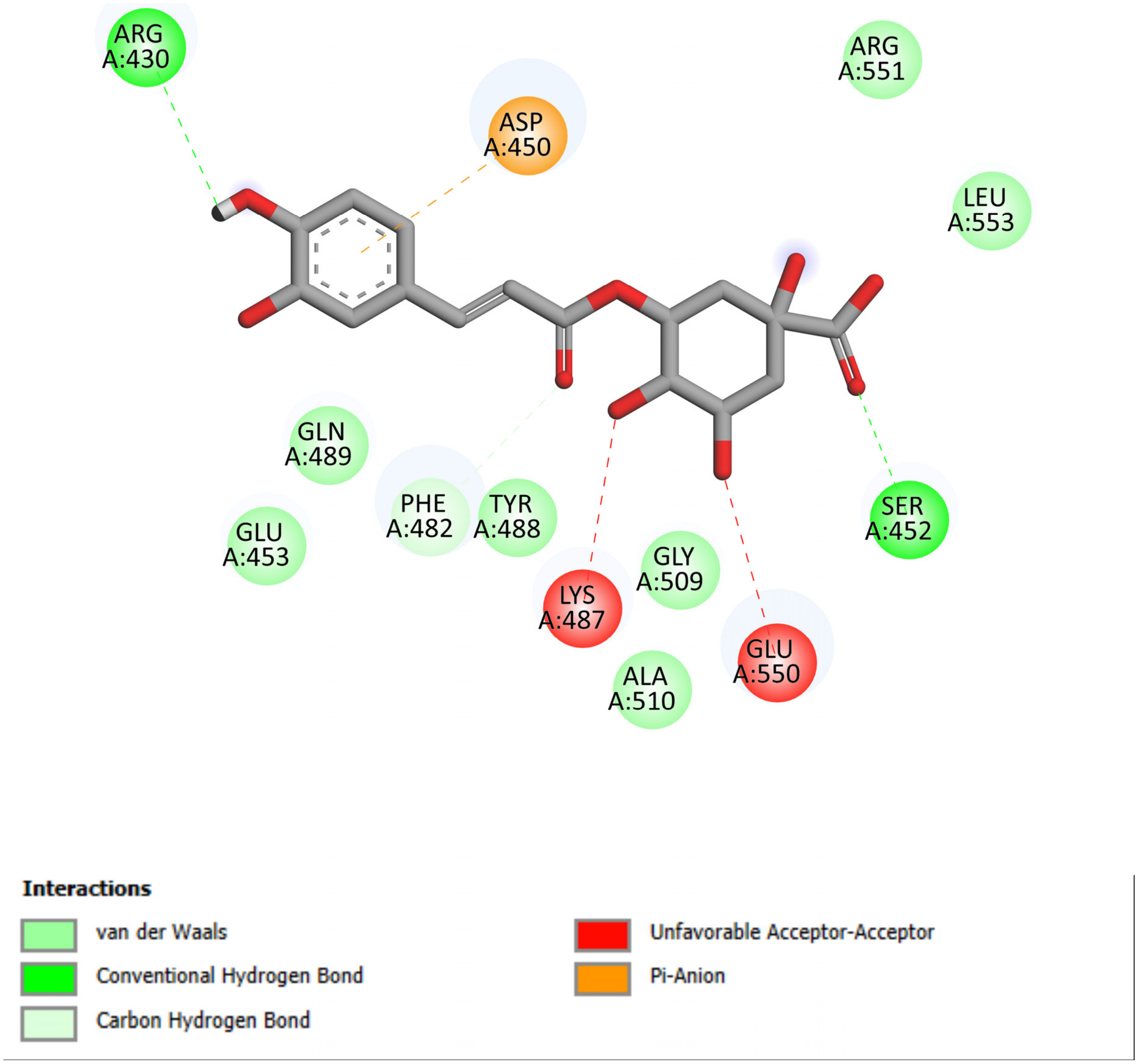

Figure S36. 2D representation of the interactions between compound **3** – ATPase (PDB ID: 1MO7) complex. Hydrogen atoms have been omitted in some cases for clarity.

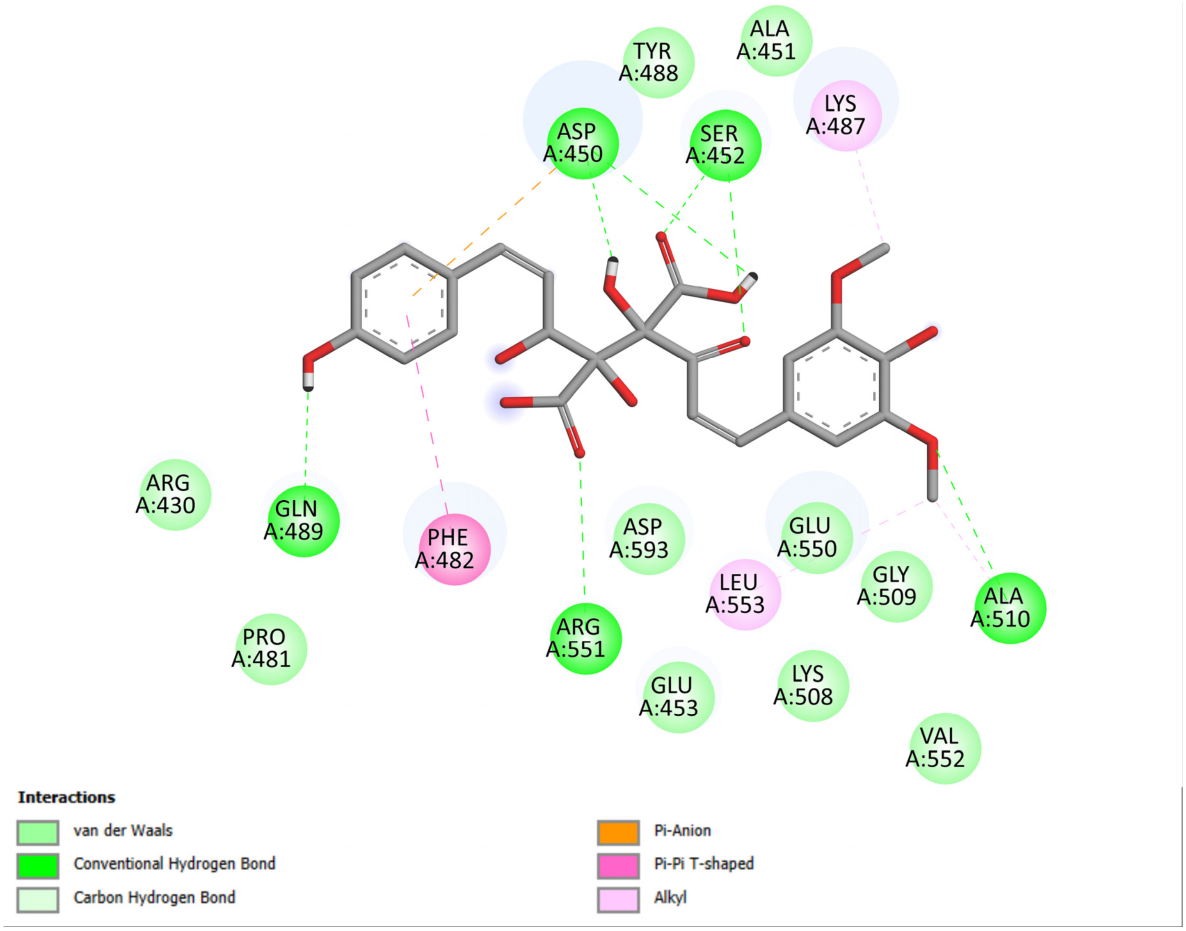

Figure S37. 2D representation of the interactions between compound **4** – ATPase (PDB ID: 1MO7) complex. Hydrogen atoms have been omitted in some cases for clarity.

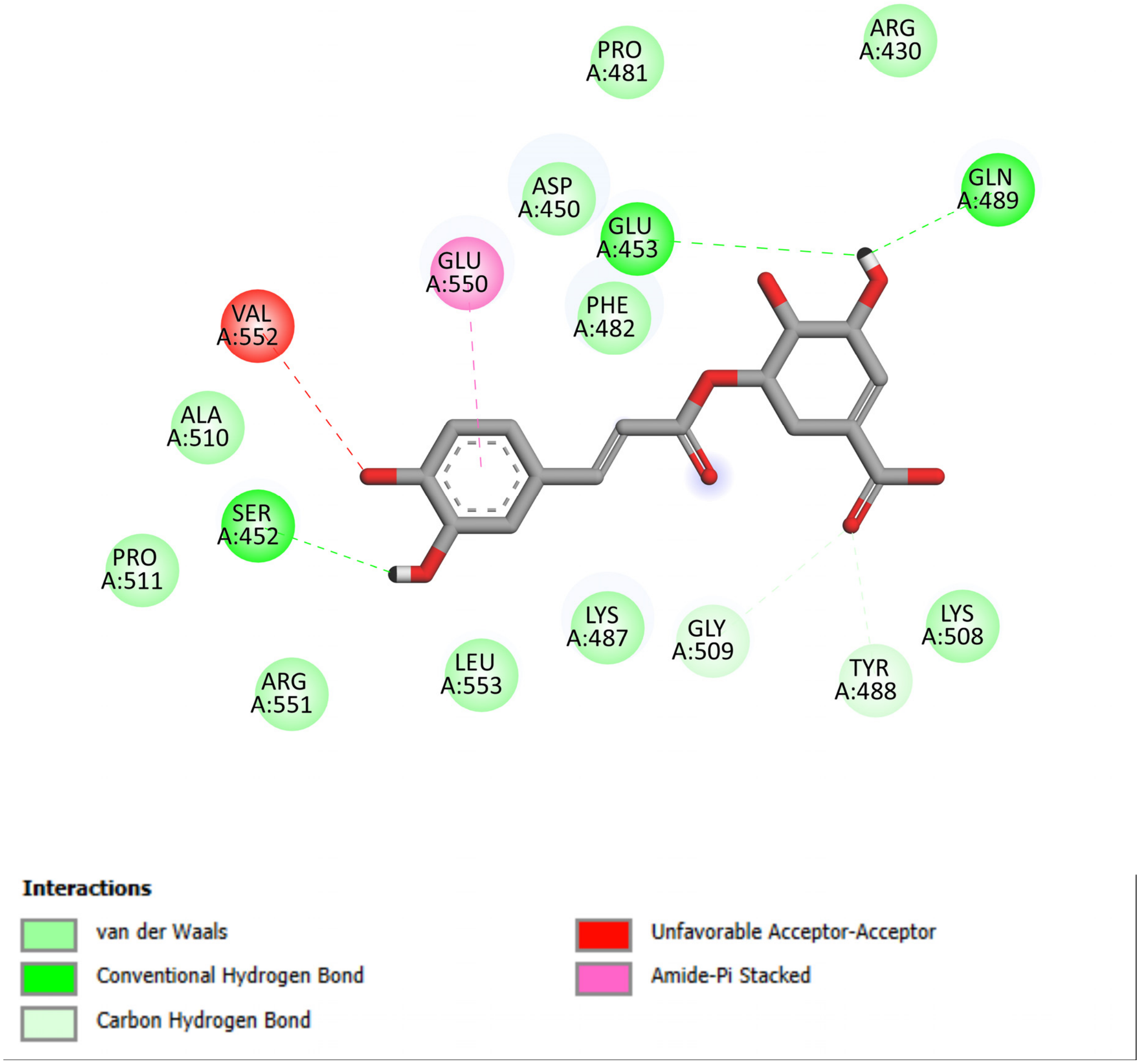

Figure S38. 2D representation of the interactions between compound **5** – ATPase (PDB ID: 1MO7) complex. Hydrogen atoms have been omitted in some cases for clarity.

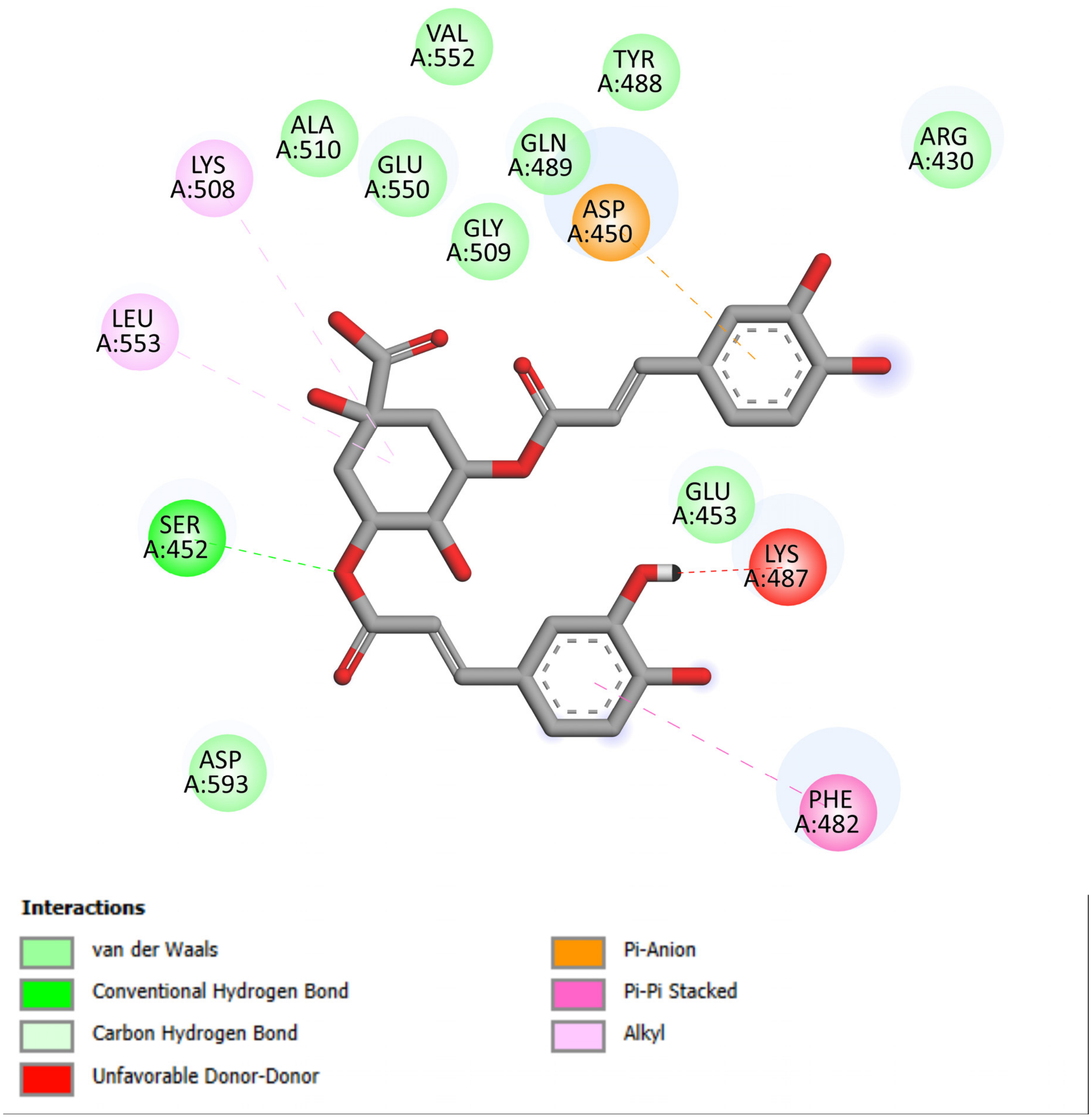

Figure S39. 2D representation of the interactions between compound **6** – ATPase (PDB ID: 1MO7) complex. Hydrogen atoms have been omitted in some cases for clarity.

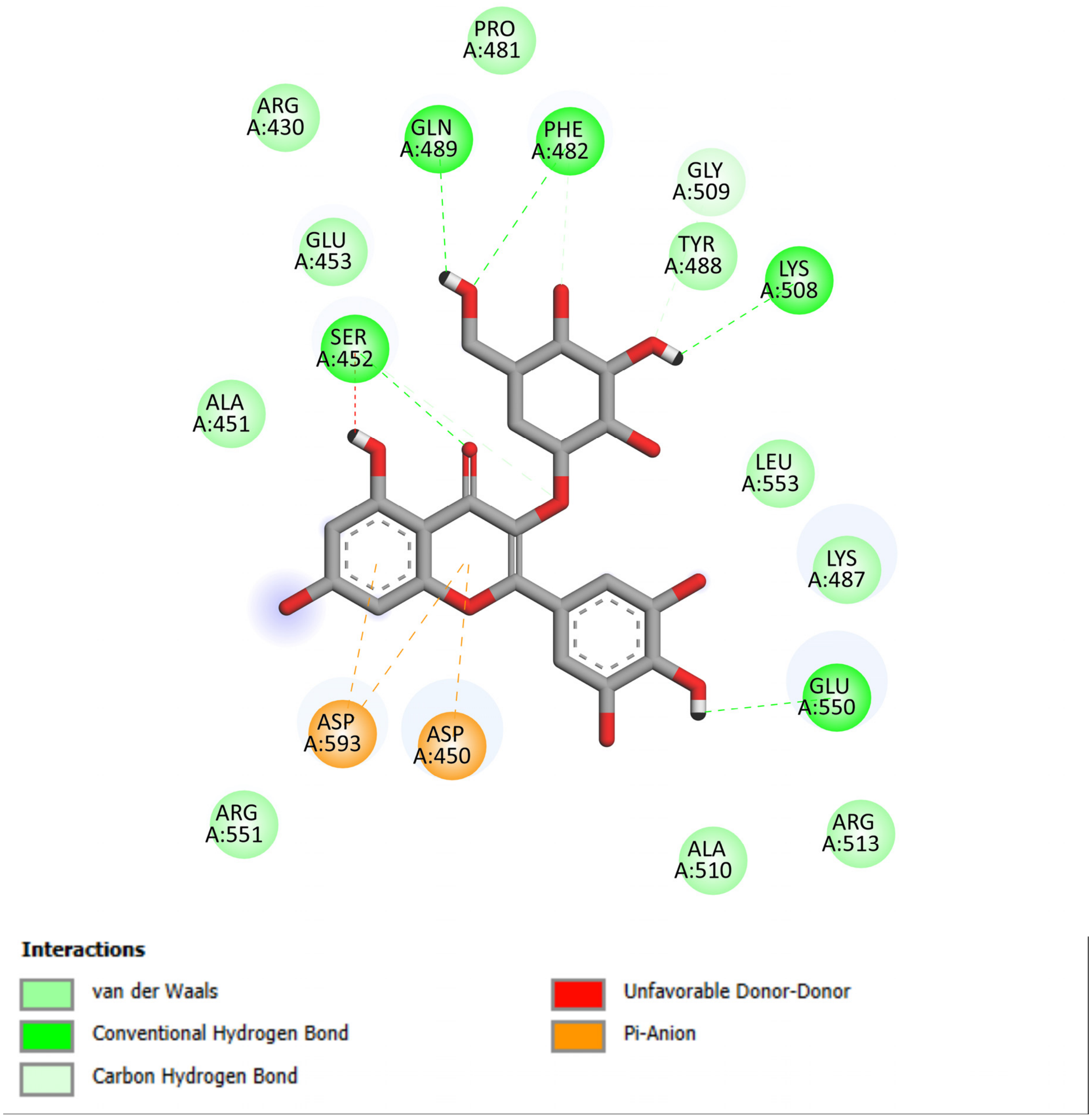

Figure S40. 2D representation of the interactions between compound **7** – ATPase (PDB ID: 1MO7) complex. Hydrogen atoms have been omitted in some cases for clarity.

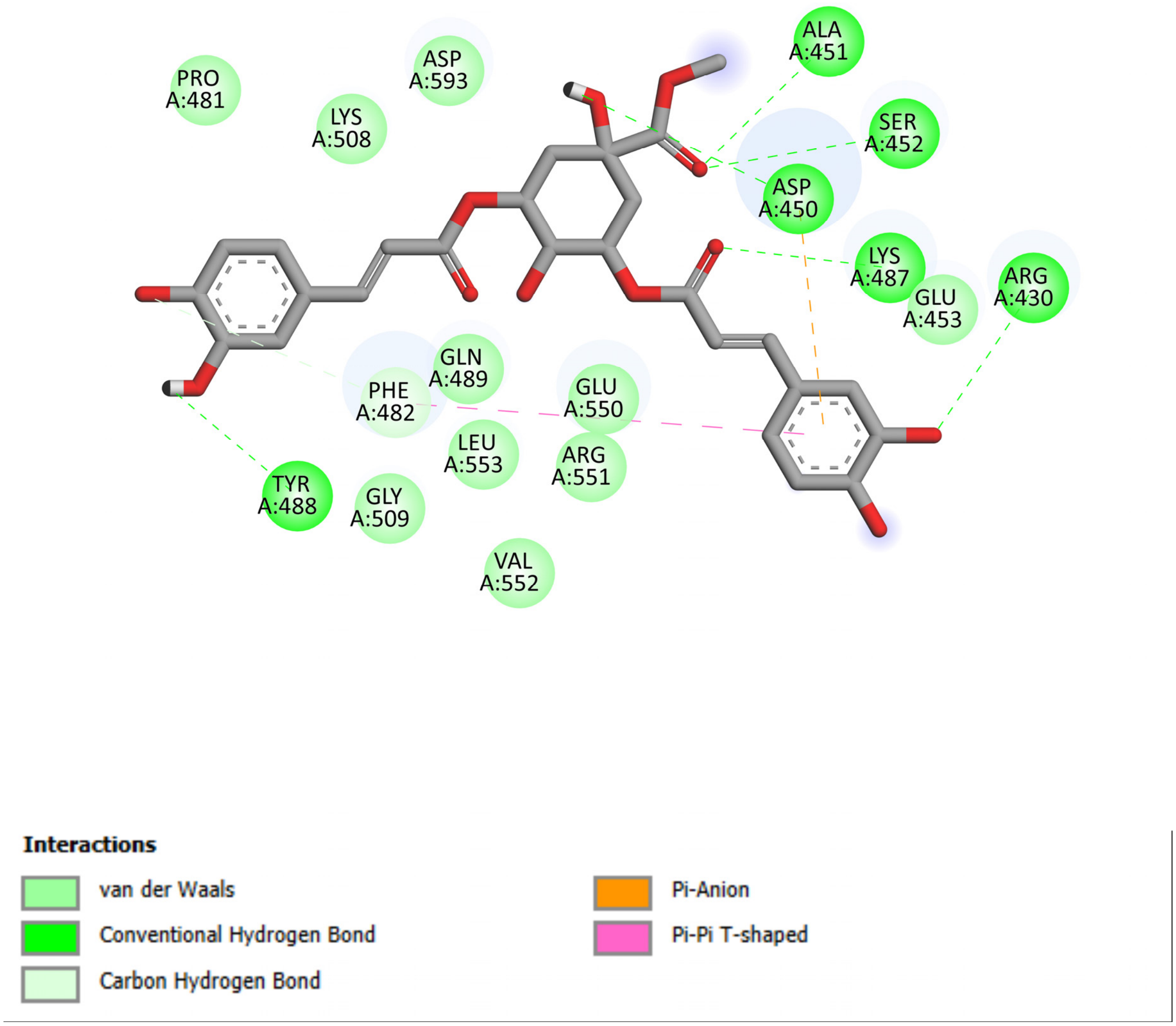

Figure S41. 2D representation of the interactions between compound **8** – ATPase (PDB ID: 1MO7) complex. Hydrogen atoms have been omitted in some cases for clarity.

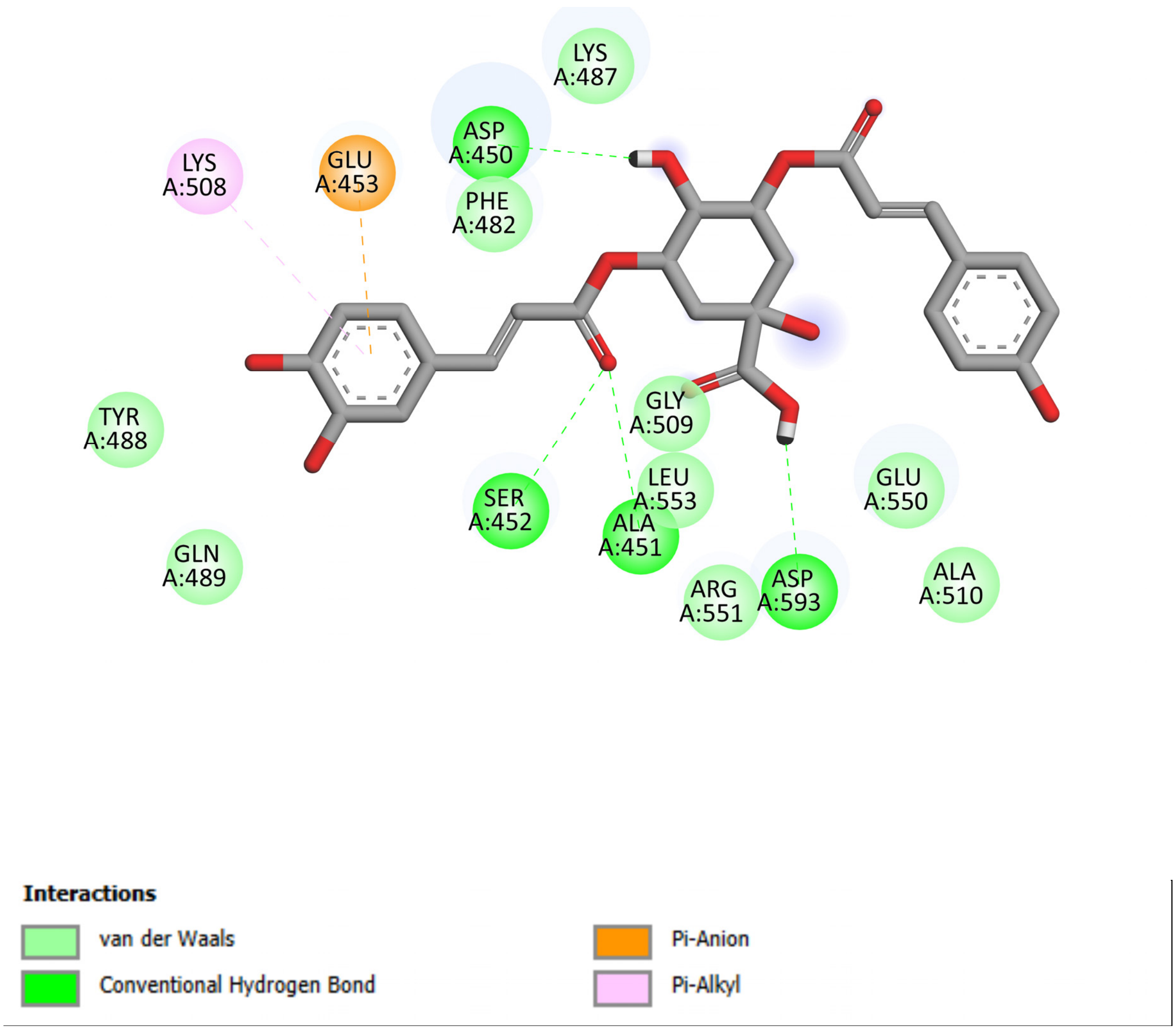

Figure S42. 2D representation of the interactions between compound **9** – ATPase (PDB ID: 1MO7) complex. Hydrogen atoms have been omitted in some cases for clarity.

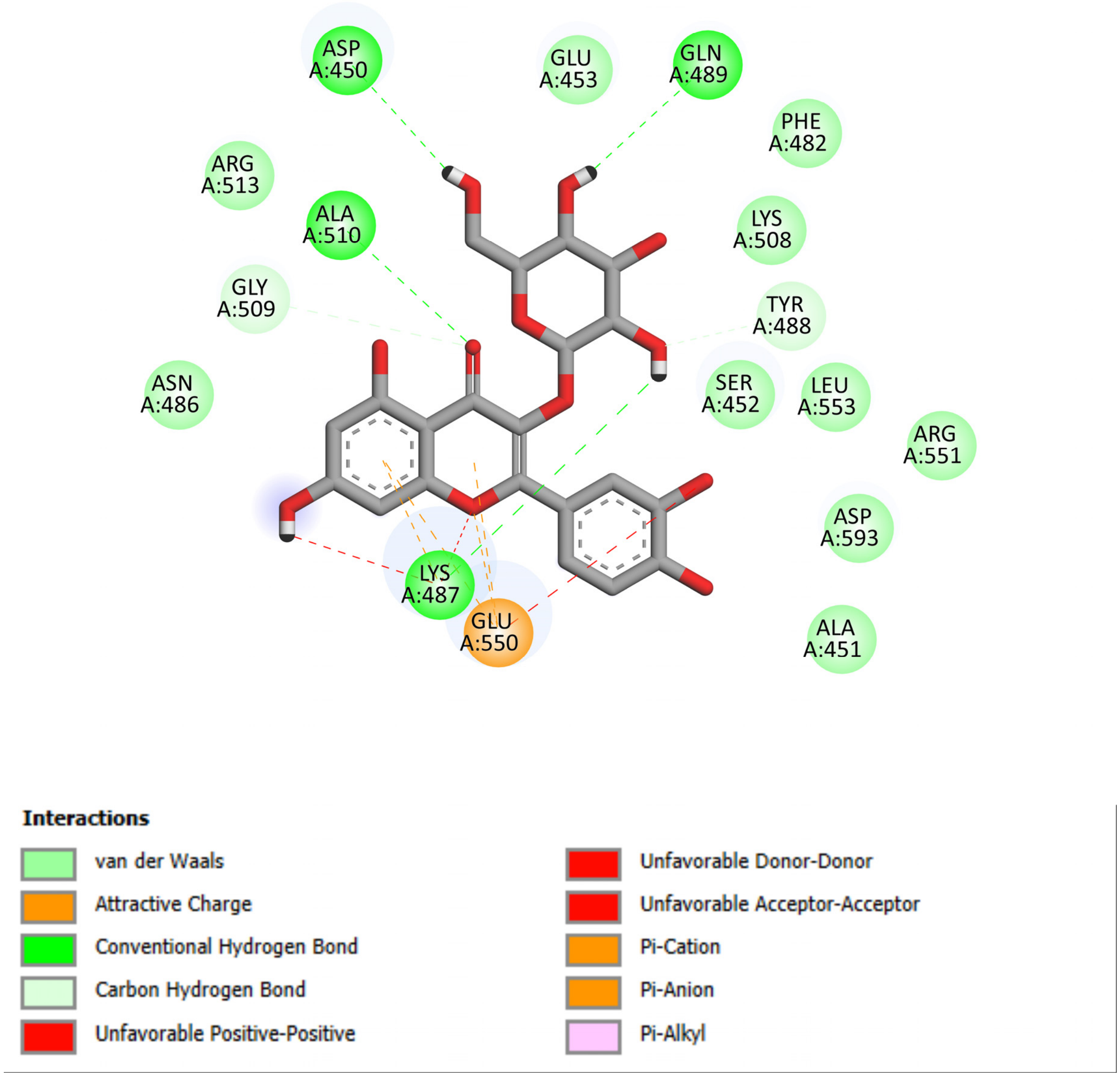

Figure S43. 2D representation of the interactions between compound **10** – ATPase (PDB ID: 1MO7) complex. Hydrogen atoms have been omitted in some cases for clarity.

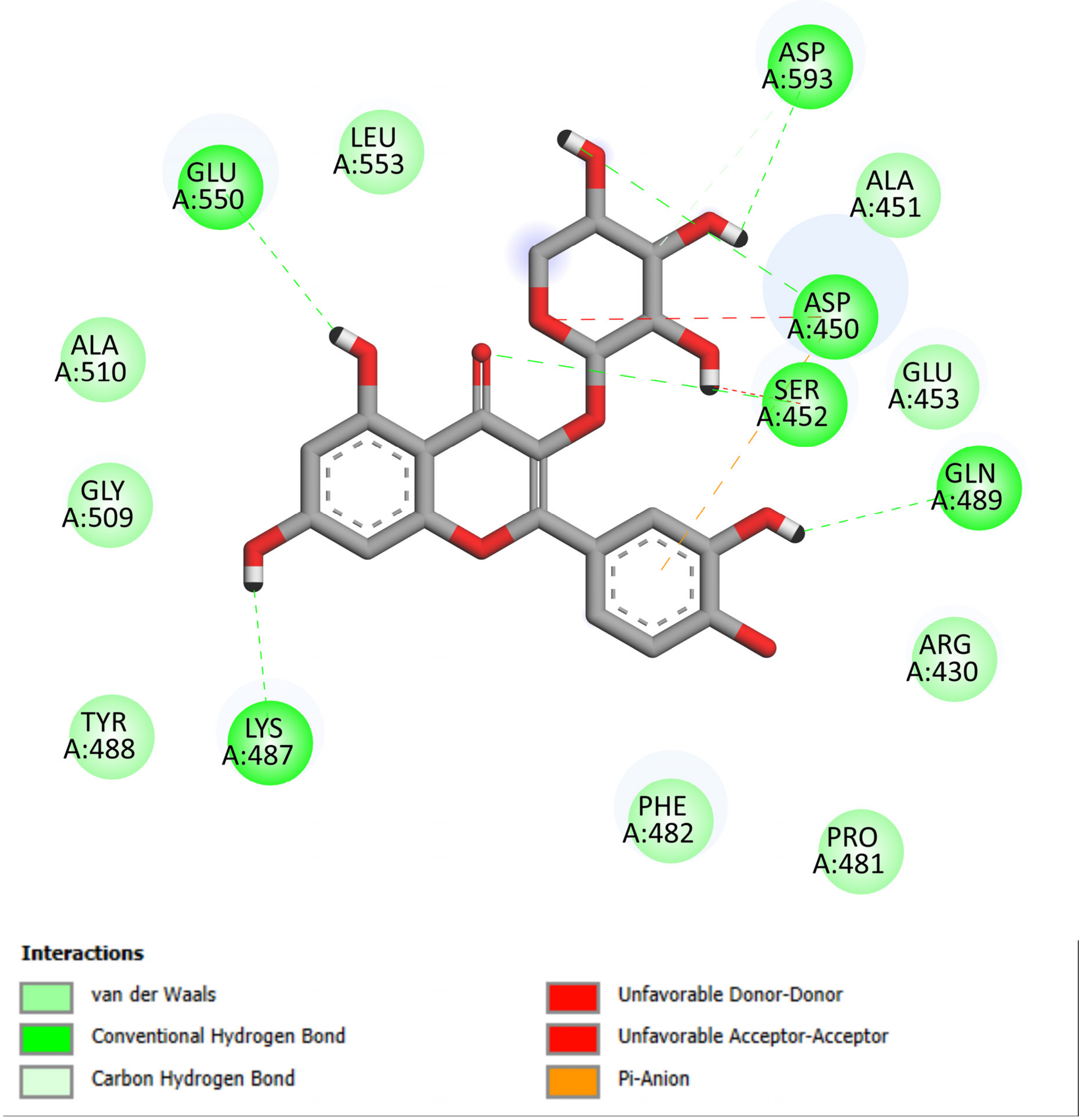

Figure S44. 2D representation of the interactions between compound **11** – ATPase (PDB ID: 1MO7) complex. Hydrogen atoms have been omitted in some cases for clarity.

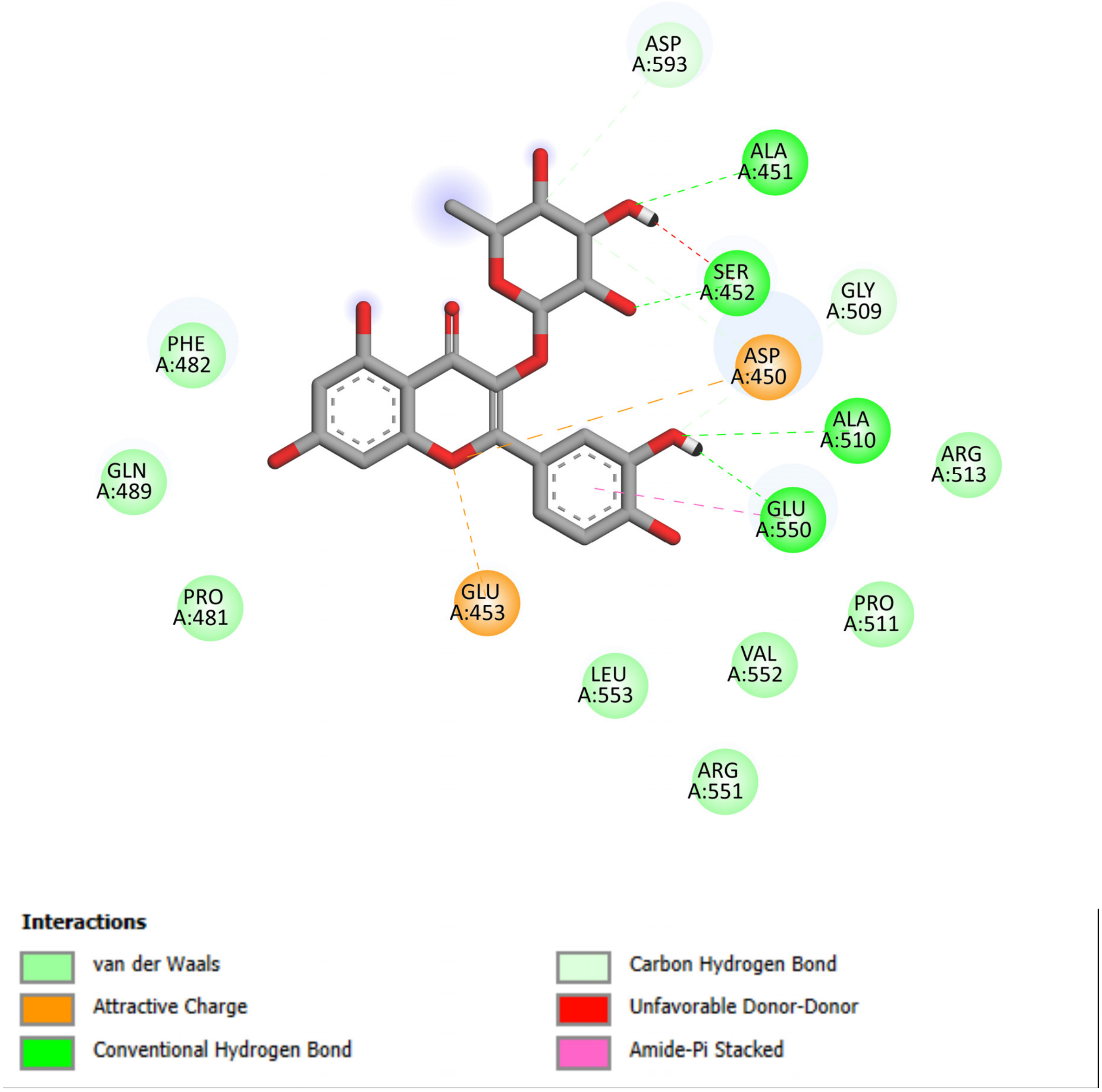

Figure S45. 2D representation of the interactions between compound **1** – Monoamine oxidase (PDB ID: 1O5W) complex. Hydrogen atoms have been omitted in some cases for clarity.

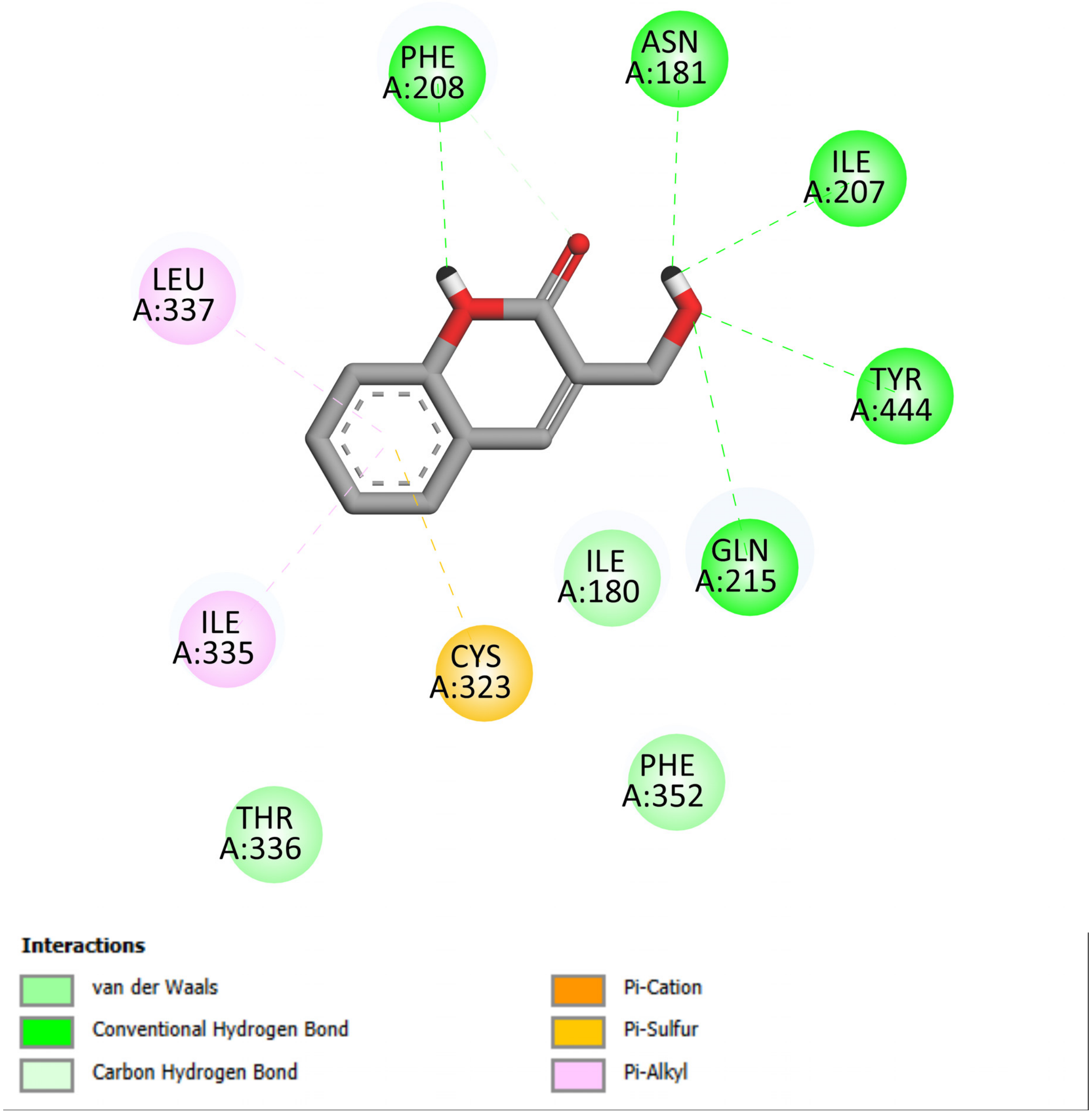

Figure S46. 2D representation of the interactions between compound **2** – Monoamine oxidase (PDB ID: 1O5W) complex. Hydrogen atoms have been omitted in some cases for clarity.

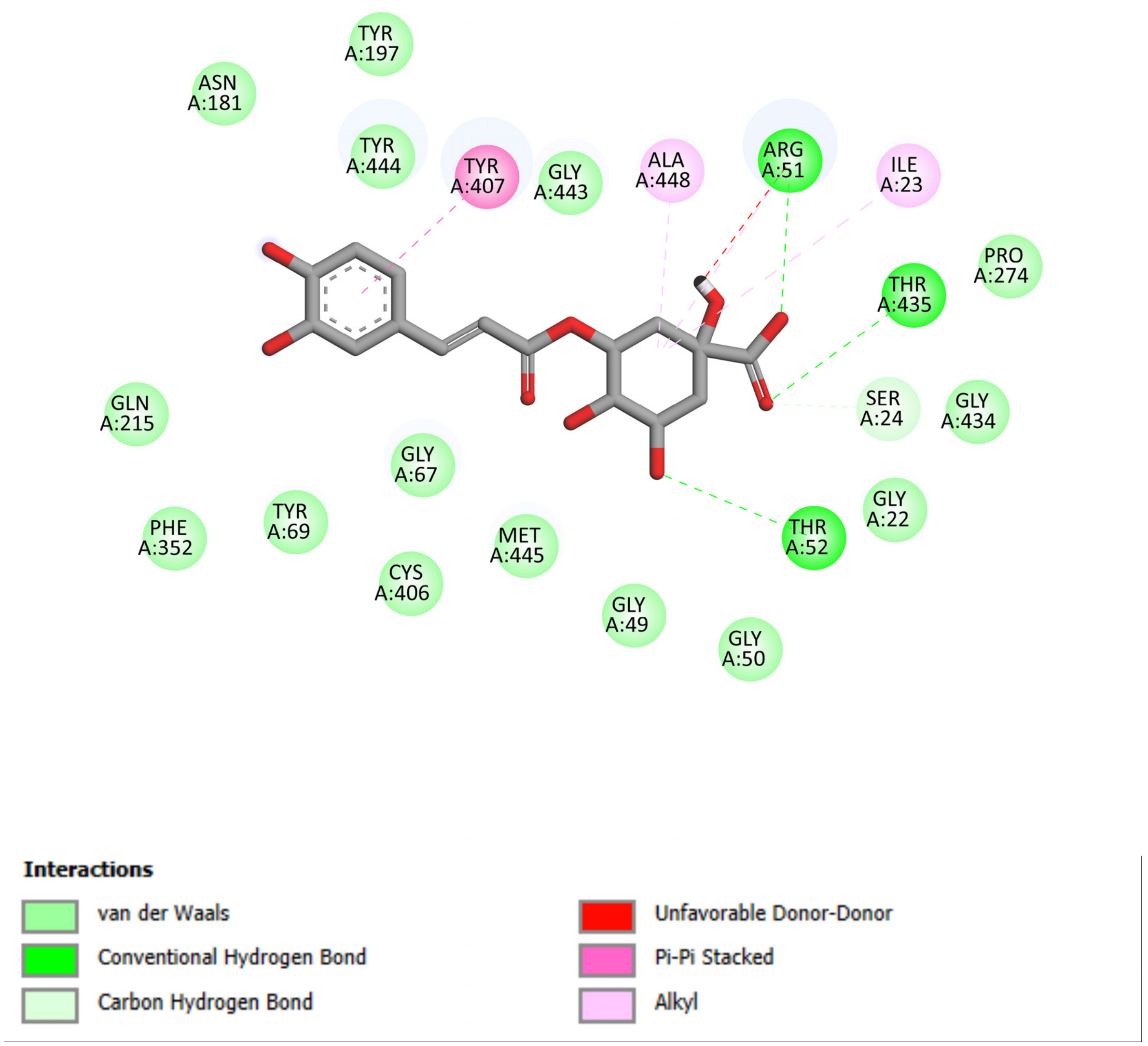

Figure S47. 2D representation of the interactions between compound **3** – Monoamine oxidase (PDB ID: 1O5W) complex. Hydrogen atoms have been omitted in some cases for clarity.

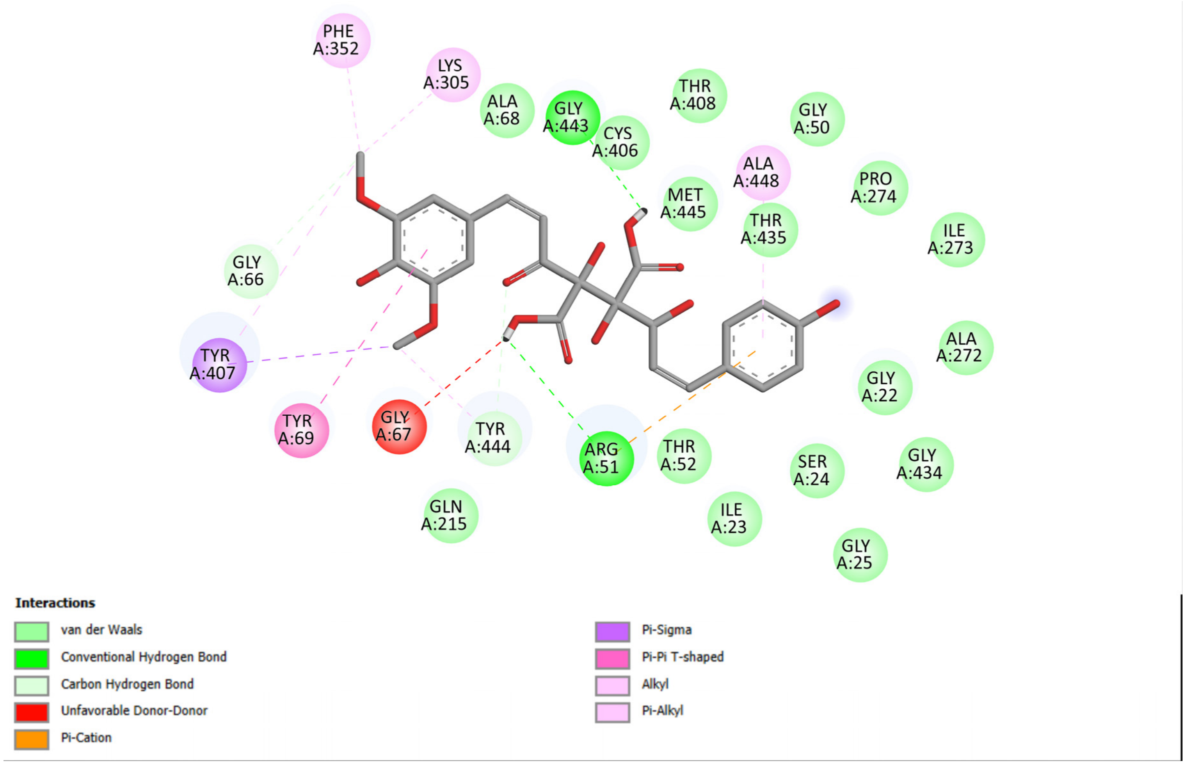

Figure S48. 2D representation of the interactions between compound **4** – Monoamine oxidase (PDB ID: 1O5W) complex. Hydrogen atoms have been omitted in some cases for clarity.

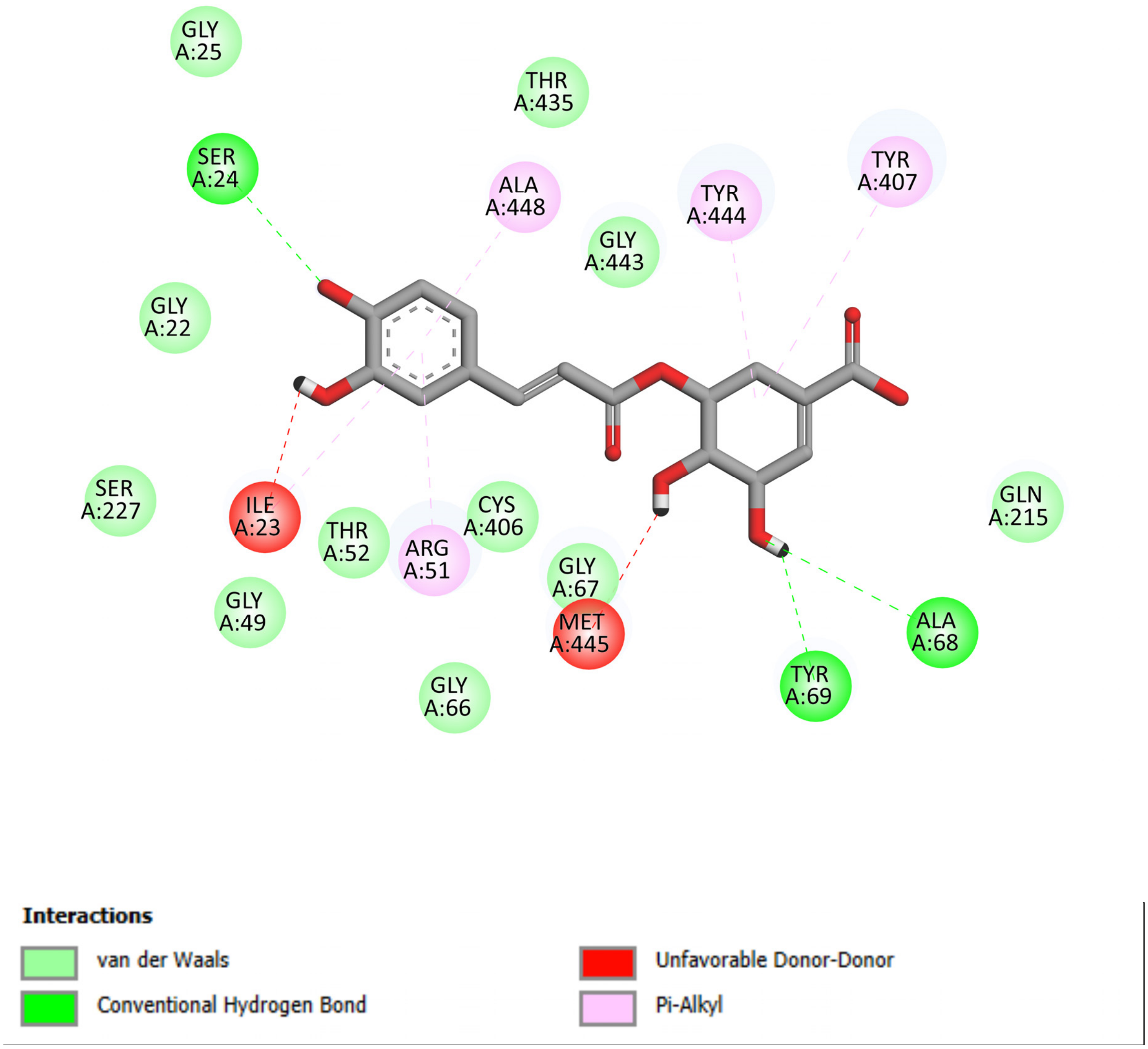

Figure S49. 2D representation of the interactions between compound **5** – Monoamine oxidase (PDB ID: 1O5W) complex. Hydrogen atoms have been omitted in some cases for clarity.

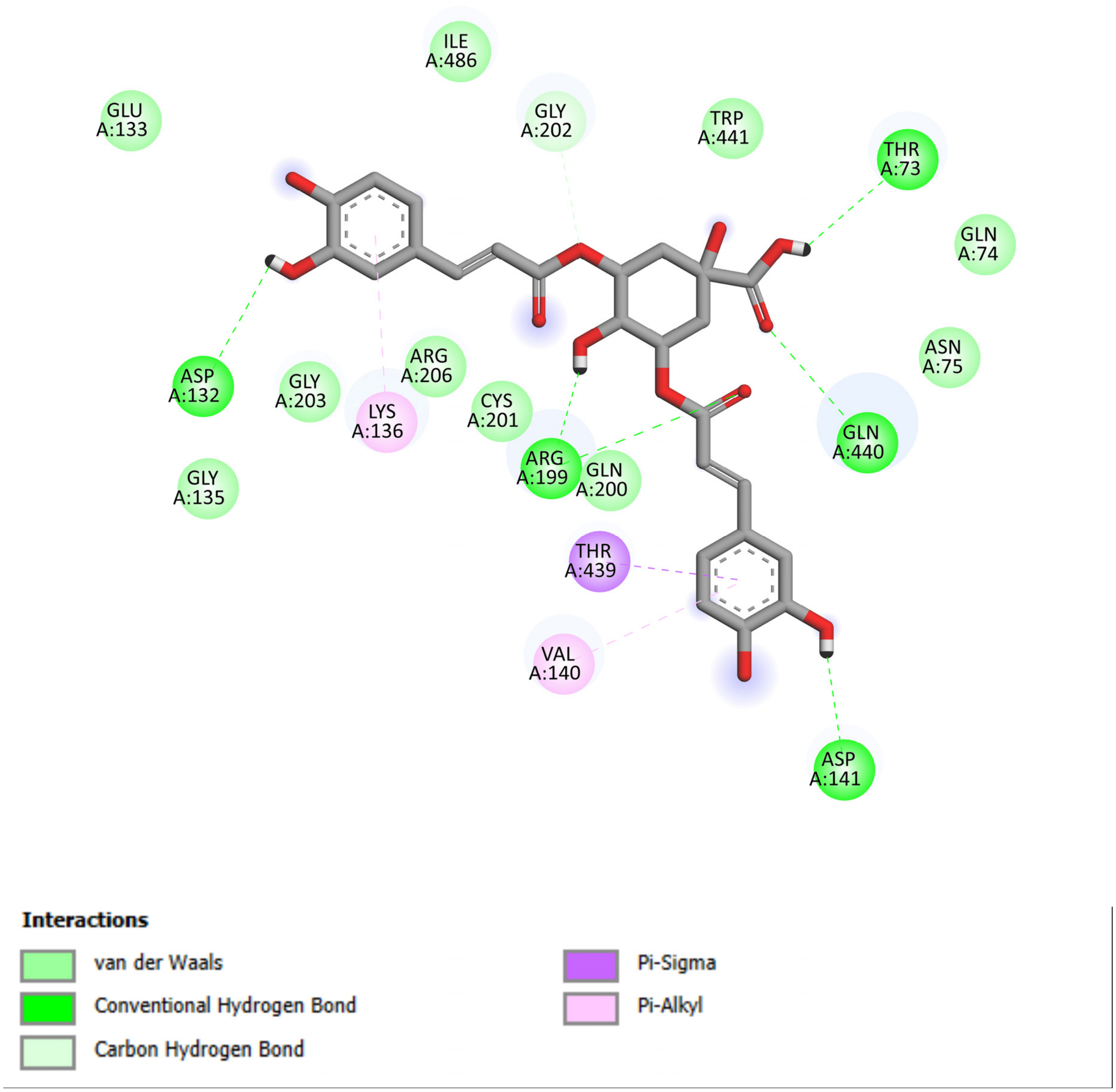

Figure S50. 2D representation of the interactions between compound **6** – Monoamine oxidase complex. Hydrogen atoms have been omitted in some cases for clarity.

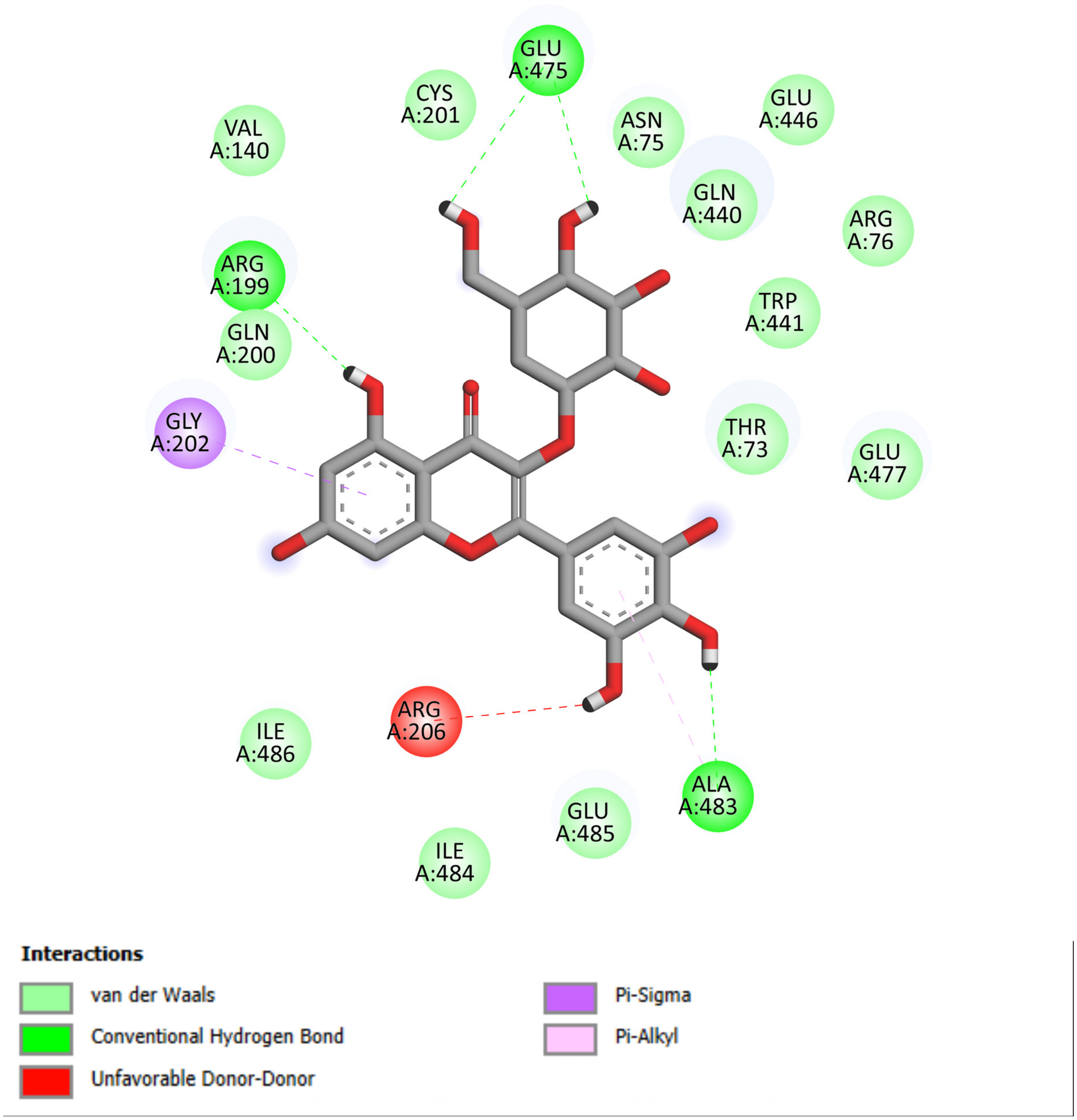

Figure S51. 2D representation of the interactions between compound **7** – Monoamine oxidase (PDB ID: 1O5W) complex. Hydrogen atoms have been omitted in some cases for clarity.

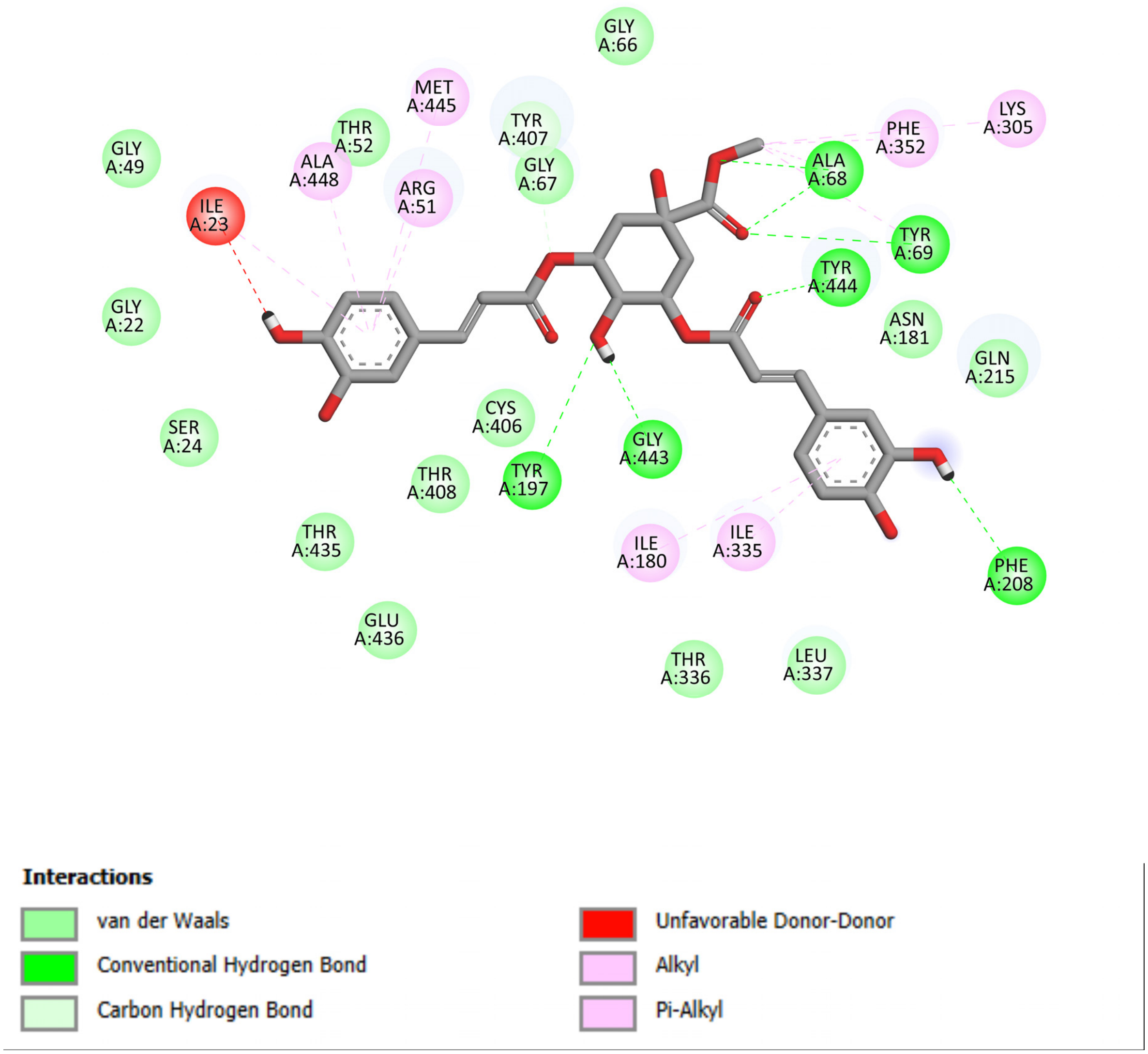

Figure S52. 2D representation of the interactions between compound **9** – Monoamine oxidase (PDB ID: 1O5W) complex. Hydrogen atoms have been omitted in some cases for clarity.

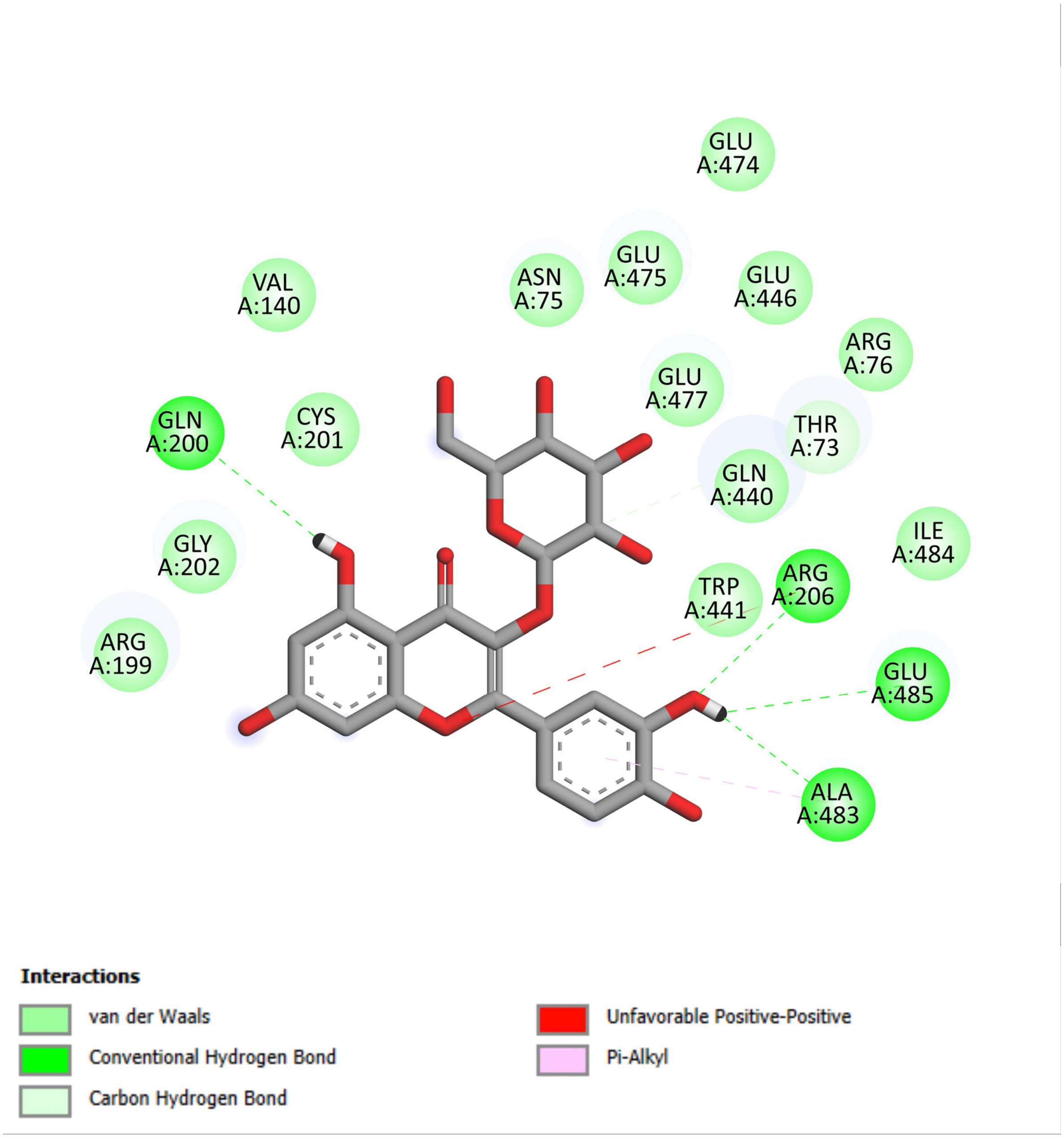

Figure S53. 2D representation of the interactions between compound **10** – Monoamine oxidase (PDB ID: 1O5W) complex. Hydrogen atoms have been omitted in some cases for clarity.

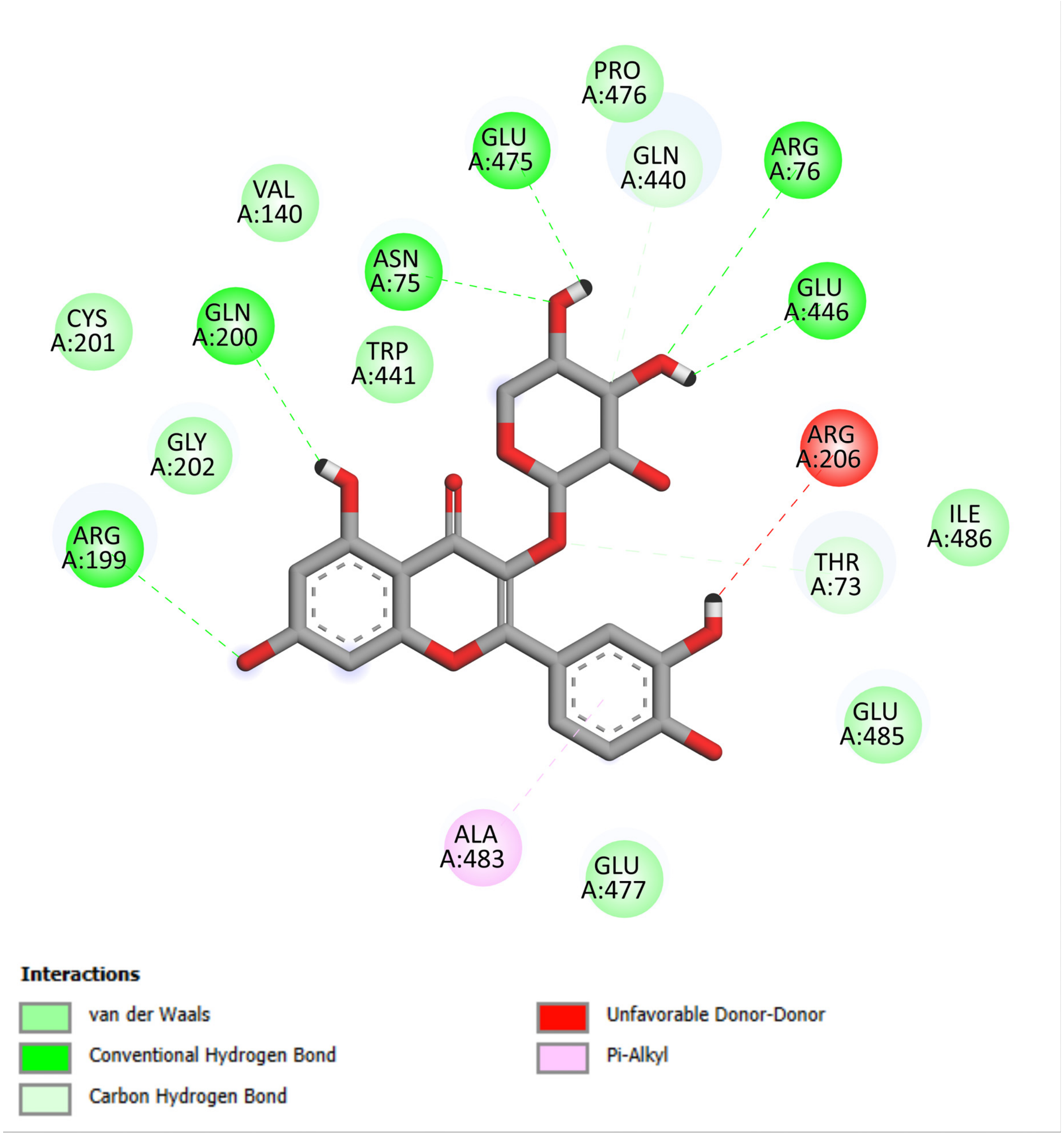

Figure S54. 2D representation of the interactions between compound **11** – Monoamine oxidase (PDB ID: 1O5W) complex. Hydrogen atoms have been omitted in some cases for clarity.

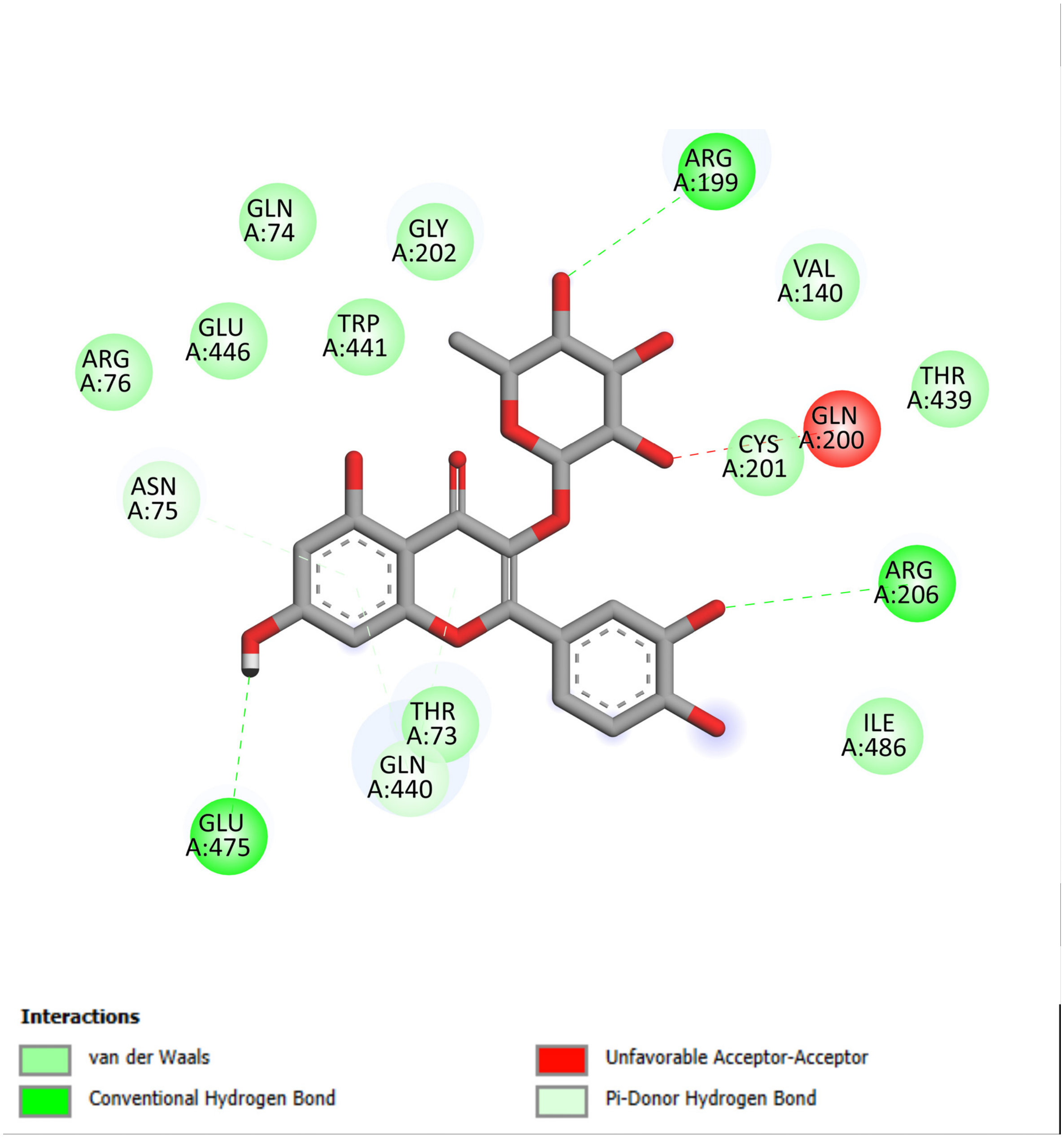

Figure S55. 2D representation of the interactions between compound **1** – serotonin receptor 5HT-2 (PDB ID: 6A94) complex. Hydrogen atoms have been omitted in some cases for clarity.

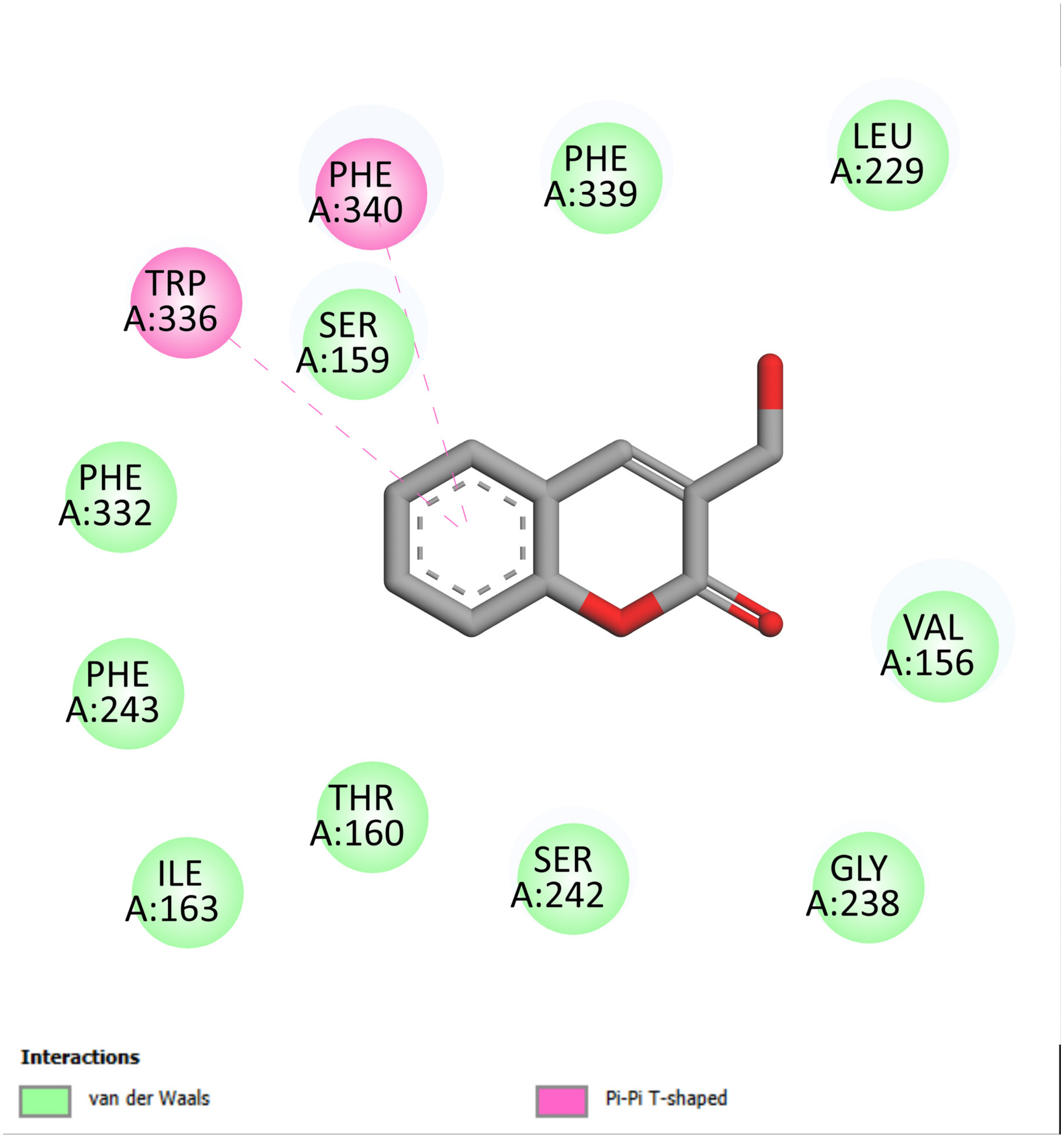

Figure S56. 2D representation of the interactions between compound **2** – serotonin receptor 5HT-2 (PDB ID: 6A94) complex. Hydrogen atoms have been omitted in some cases for clarity.

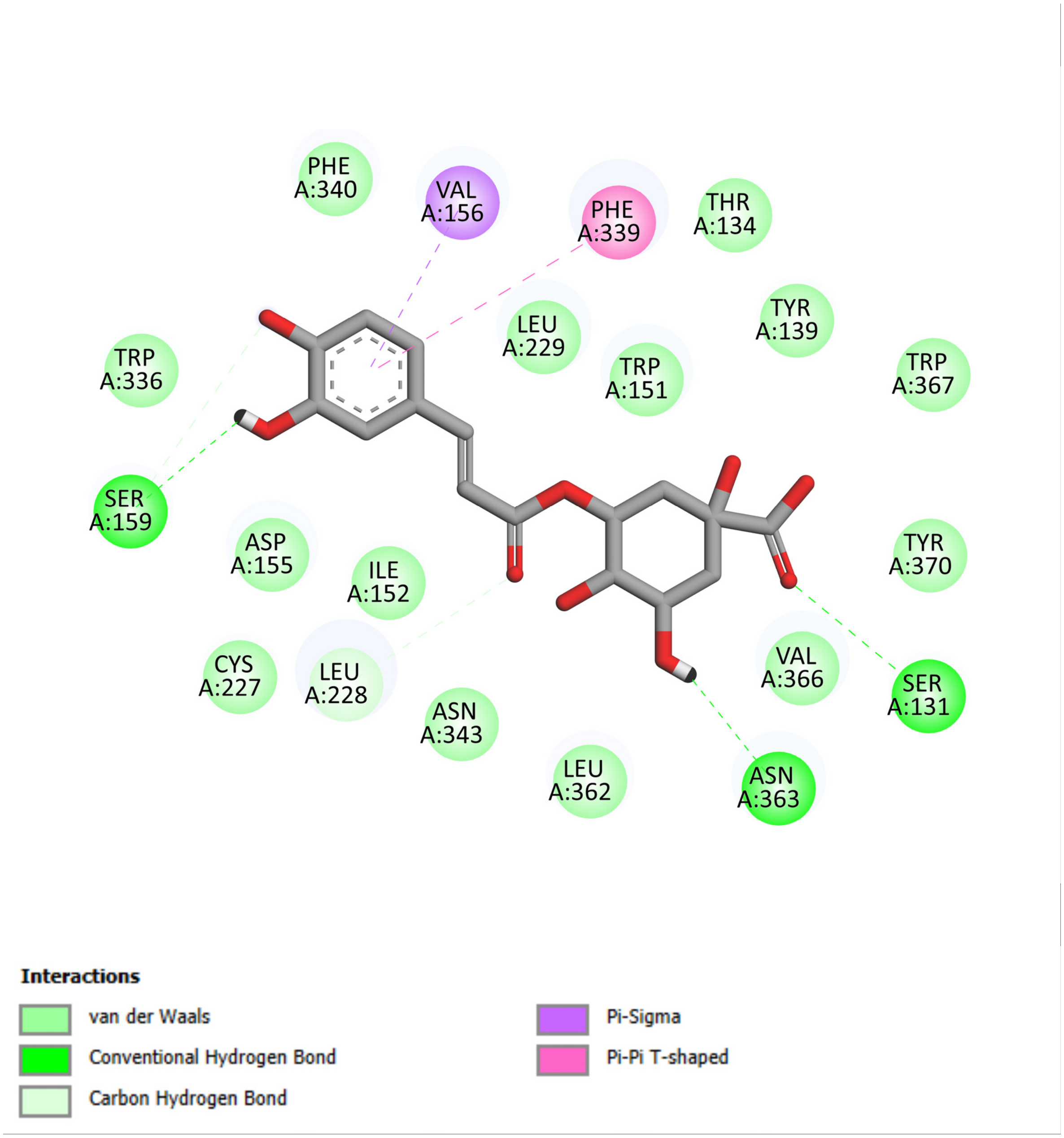

Figure S57. 2D representation of the interactions between compound **3** – serotonin receptor 5HT-2 (PDB ID: 6A94) complex. Hydrogen atoms have been omitted in some cases for clarity.

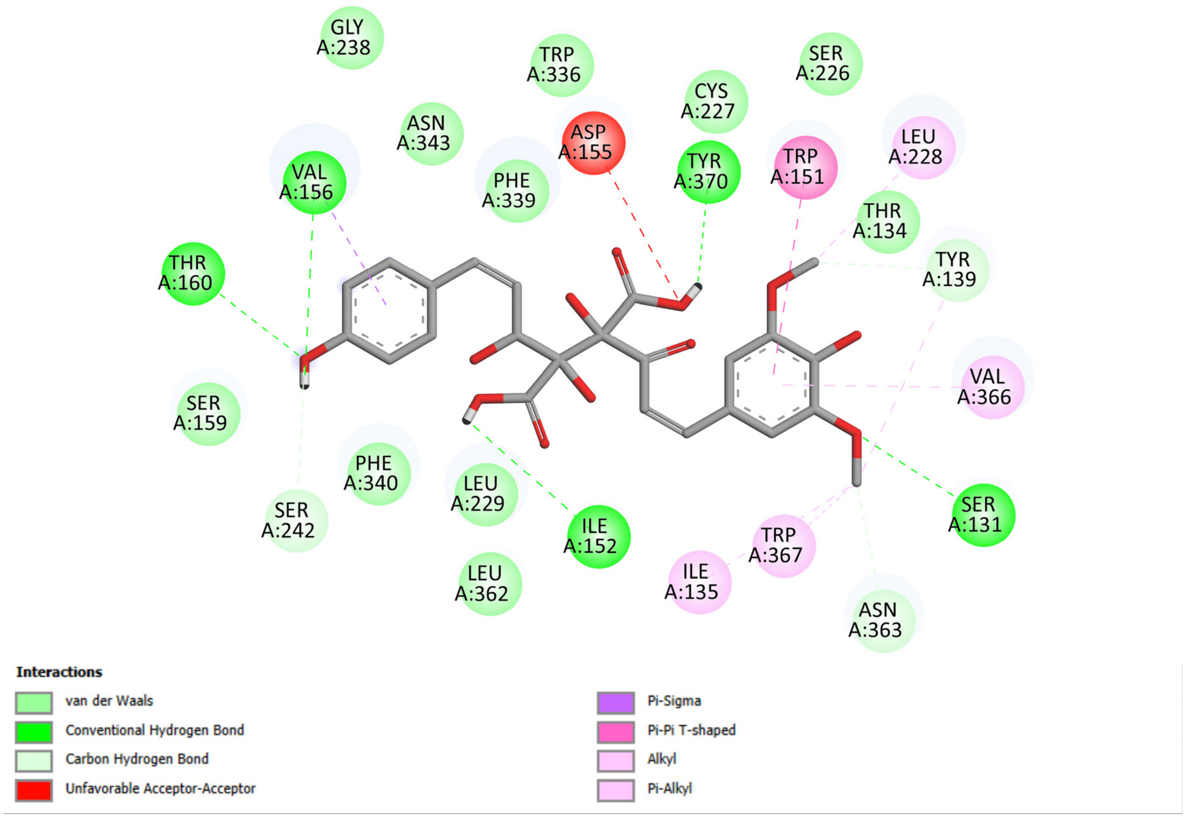

Figure S58. 2D representation of the interactions between compound **4** – serotonin receptor 5HT-2 (PDB ID: 6A94) complex. Hydrogen atoms have been omitted in some cases for clarity.

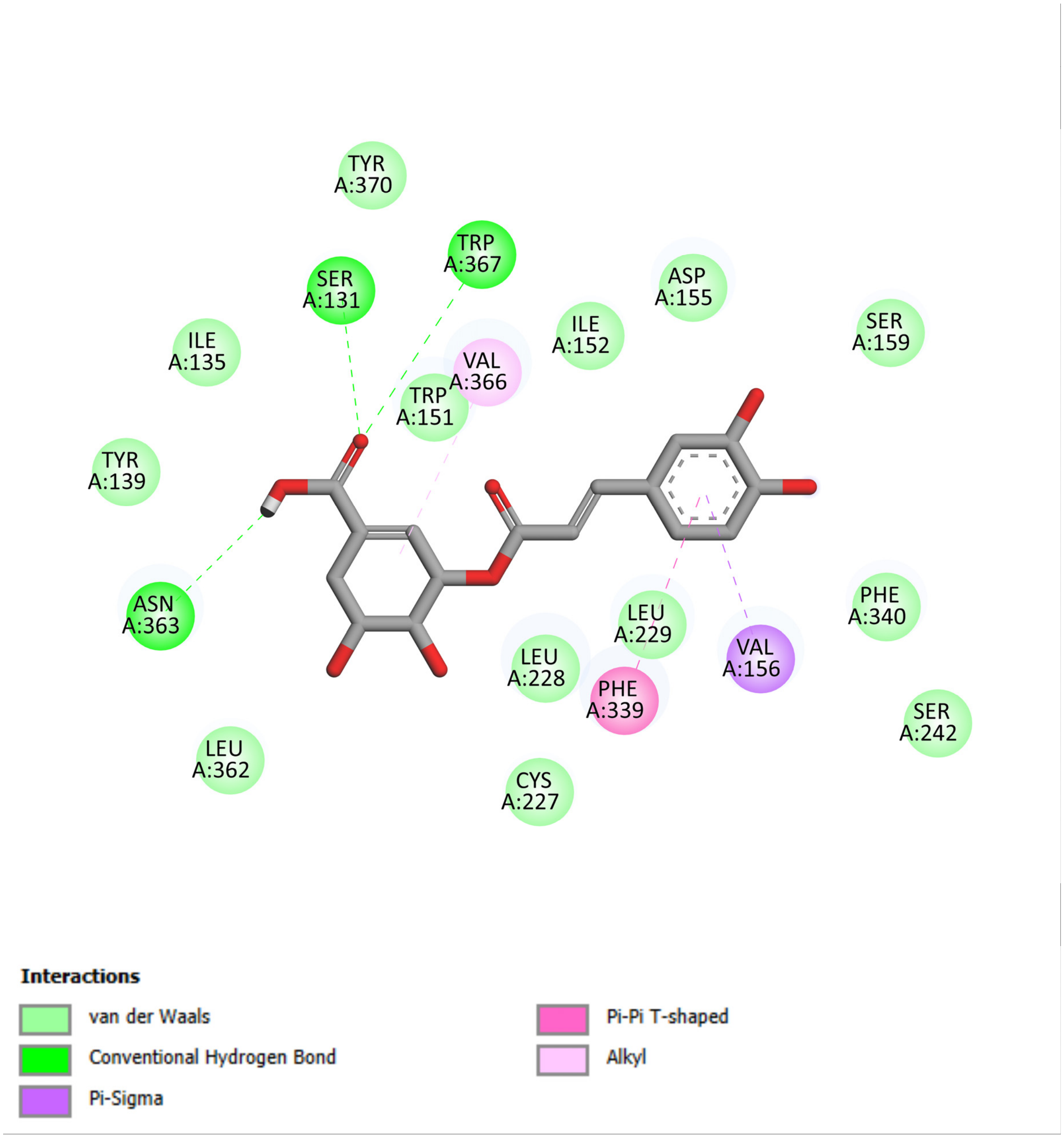

Figure S59. 2D representation of the interactions between compound **5** – serotonin receptor 5HT-2 (PDB ID: 6A94) complex. Hydrogen atoms have been omitted in some cases for clarity.

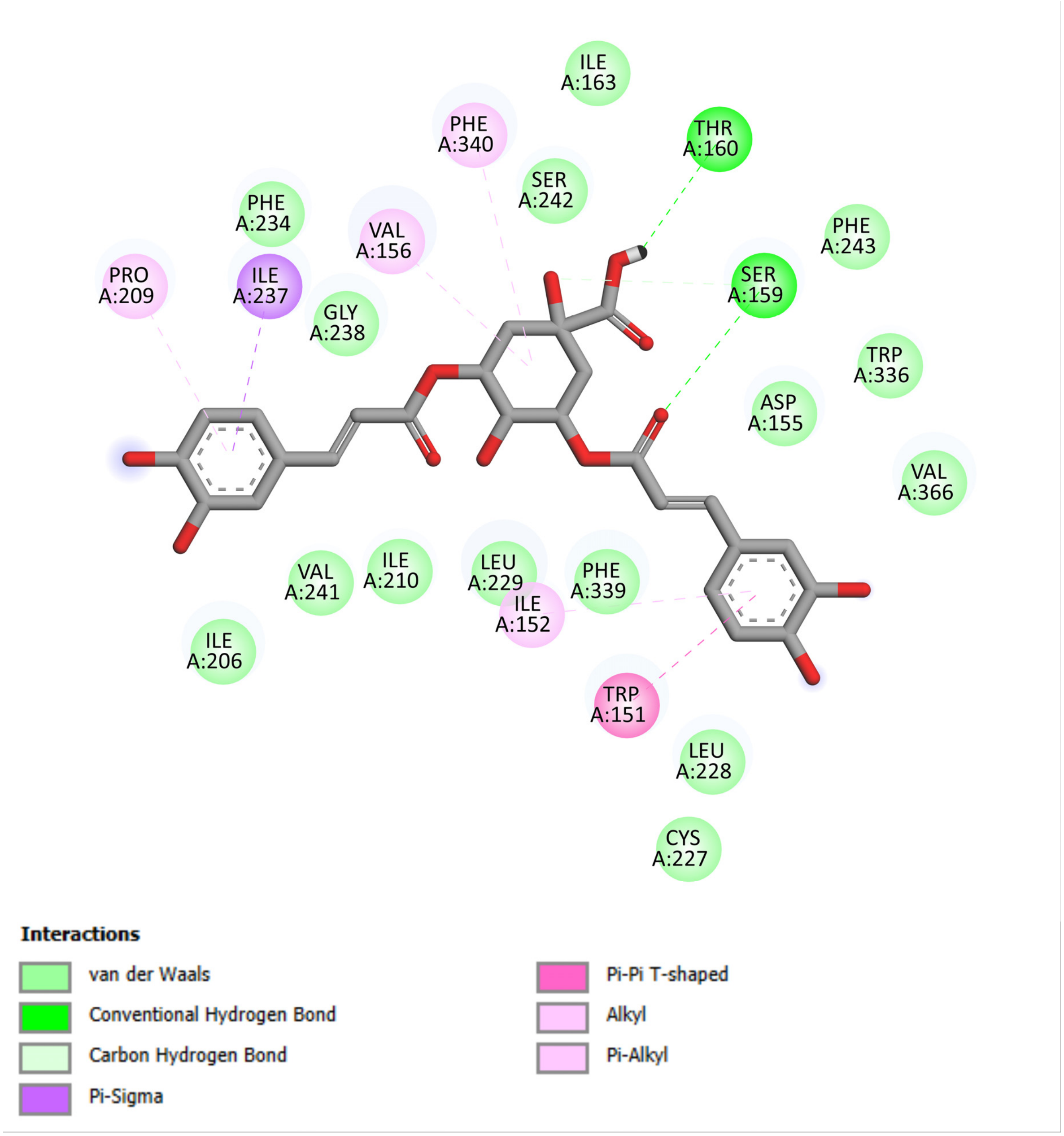

Figure S60. 2D representation of the interactions between compound **6** – serotonin receptor 5HT-2 (PDB ID: 6A94) complex. Hydrogen atoms have been omitted in some cases for clarity.

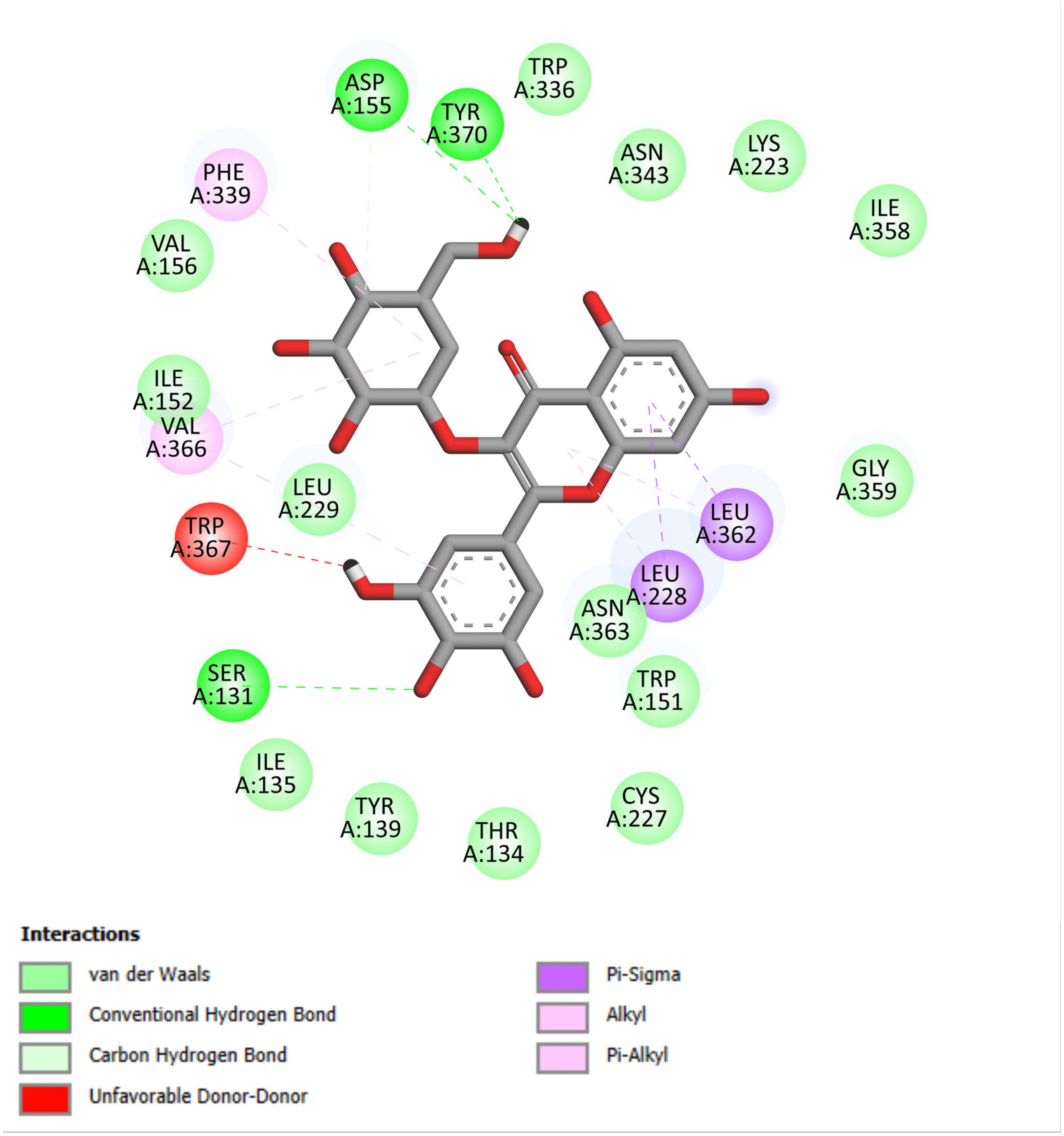

Figure S61. 2D representation of the interactions between compound **7** – serotonin receptor 5HT-2 (PDB ID: 6A94) complex. Hydrogen atoms have been omitted in some cases for clarity.

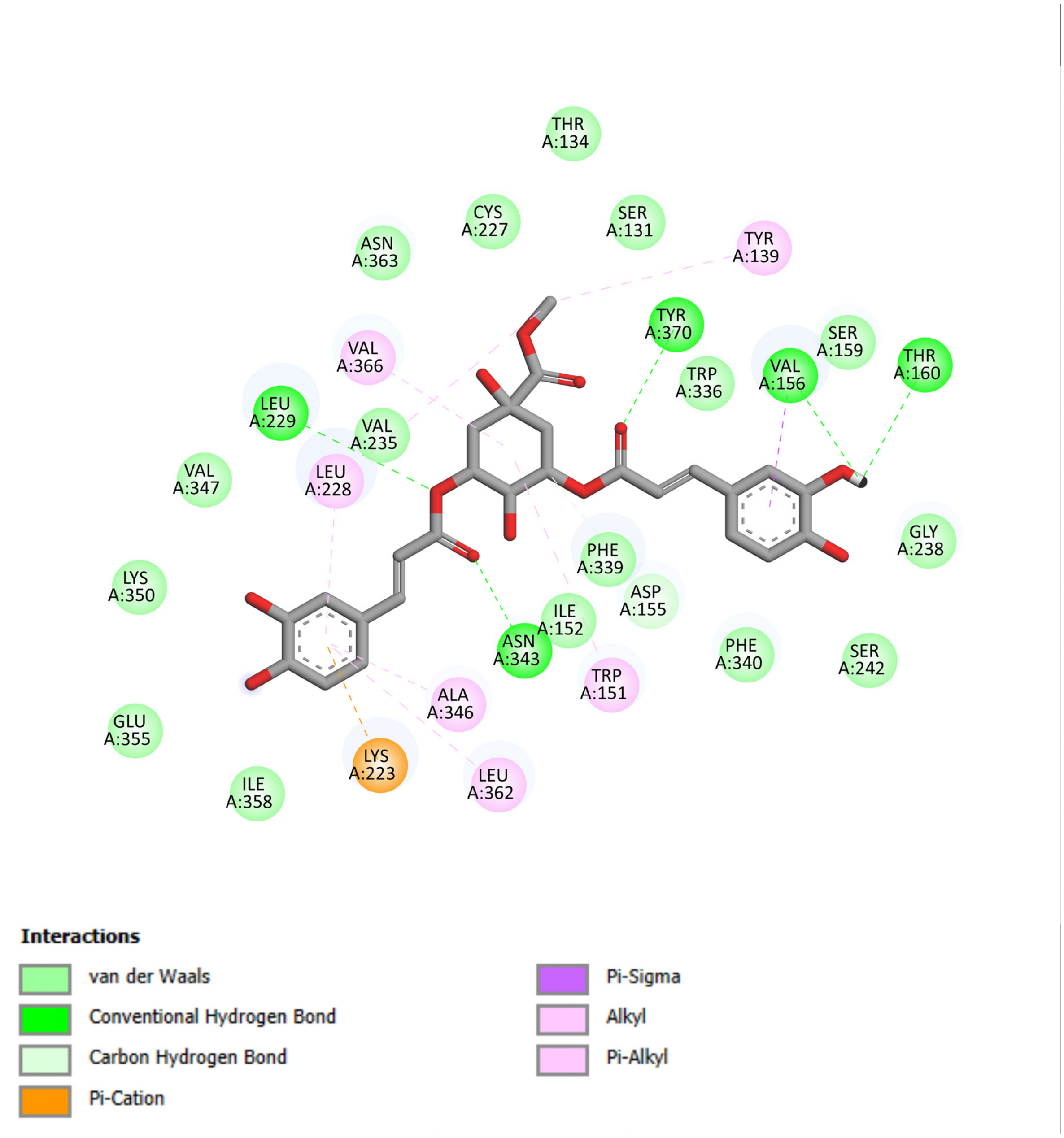

Figure S62. 2D representation of the interactions between compound **8** – serotonin receptor 5HT-2 (PDB ID: 6A94) complex. Hydrogen atoms have been omitted in some cases for clarity.

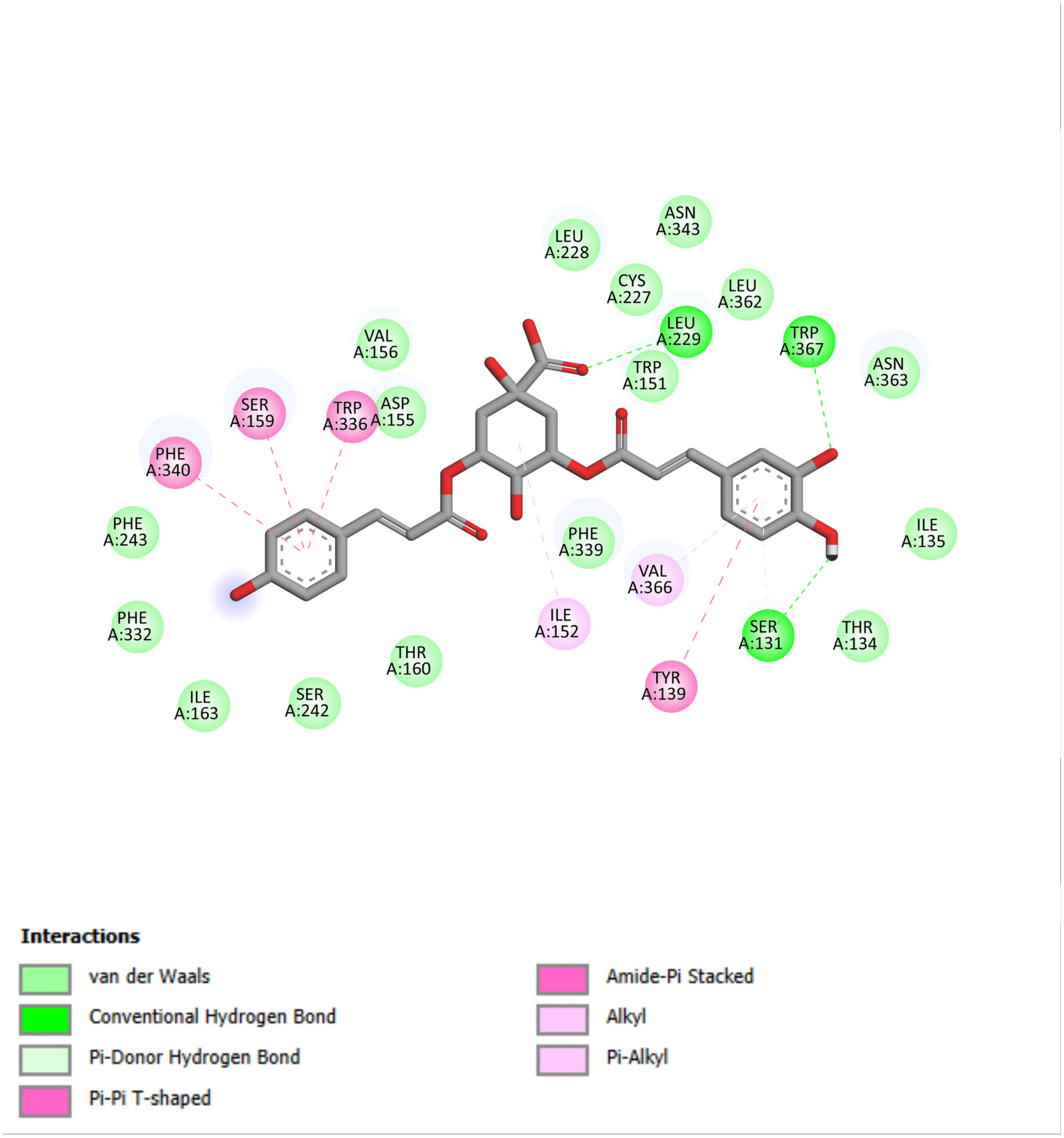

Figure S63. 2D representation of the interactions between compound **9** – serotonin receptor 5HT-2 (PDB ID: 6A94) complex. Hydrogen atoms have been omitted in some cases for clarity.

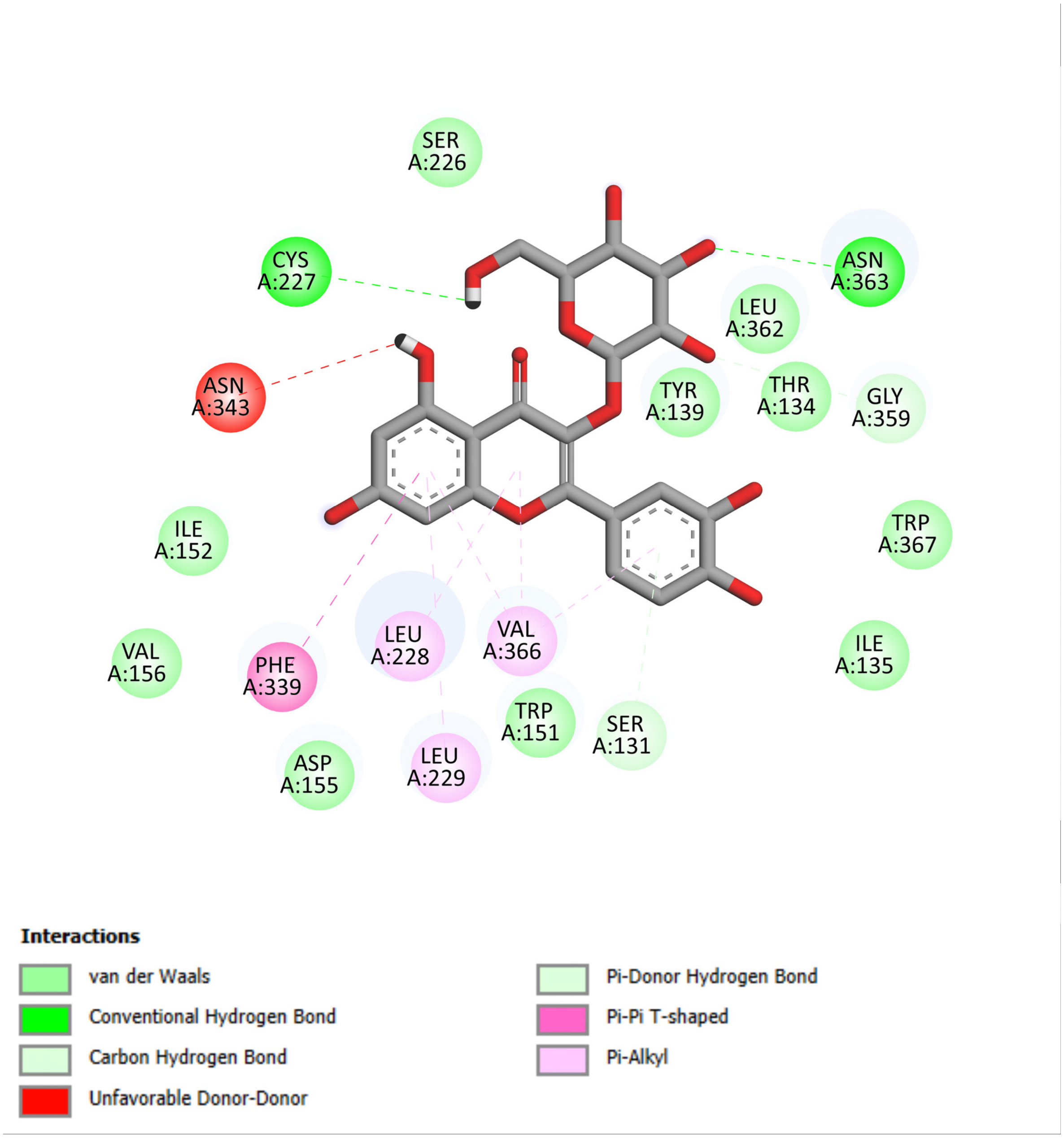

Figure S64. 2D representation of the interactions between compound **10** – serotonin receptor 5HT-2 (PDB ID: 6A94) complex. Hydrogen atoms have been omitted in some cases for clarity.

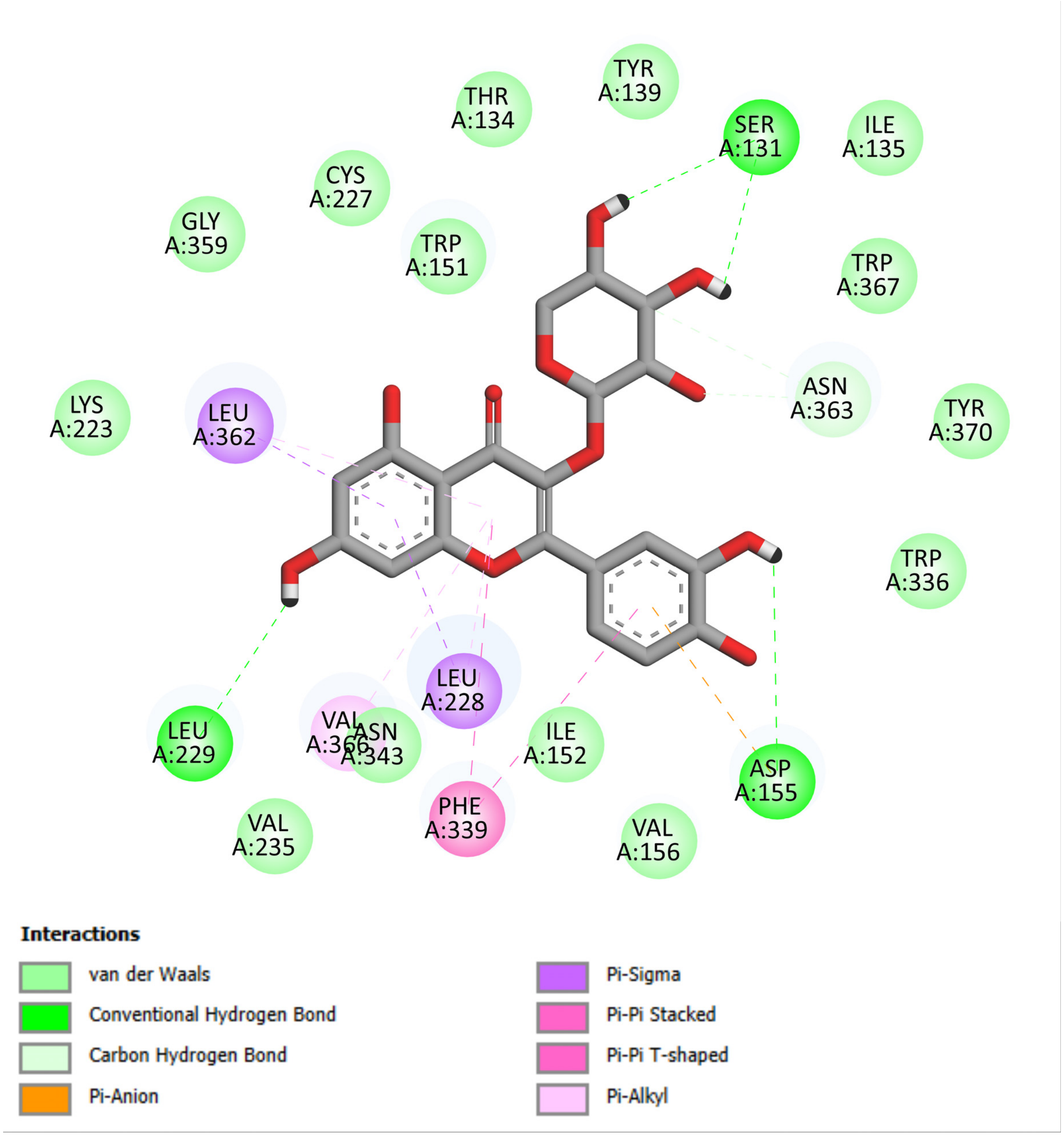

Figure S65. 2D representation of the interactions between compound **11** – serotonin receptor 5HT-2 (PDB ID: 6A94) complex. Hydrogen atoms have been omitted in some cases for clarity.

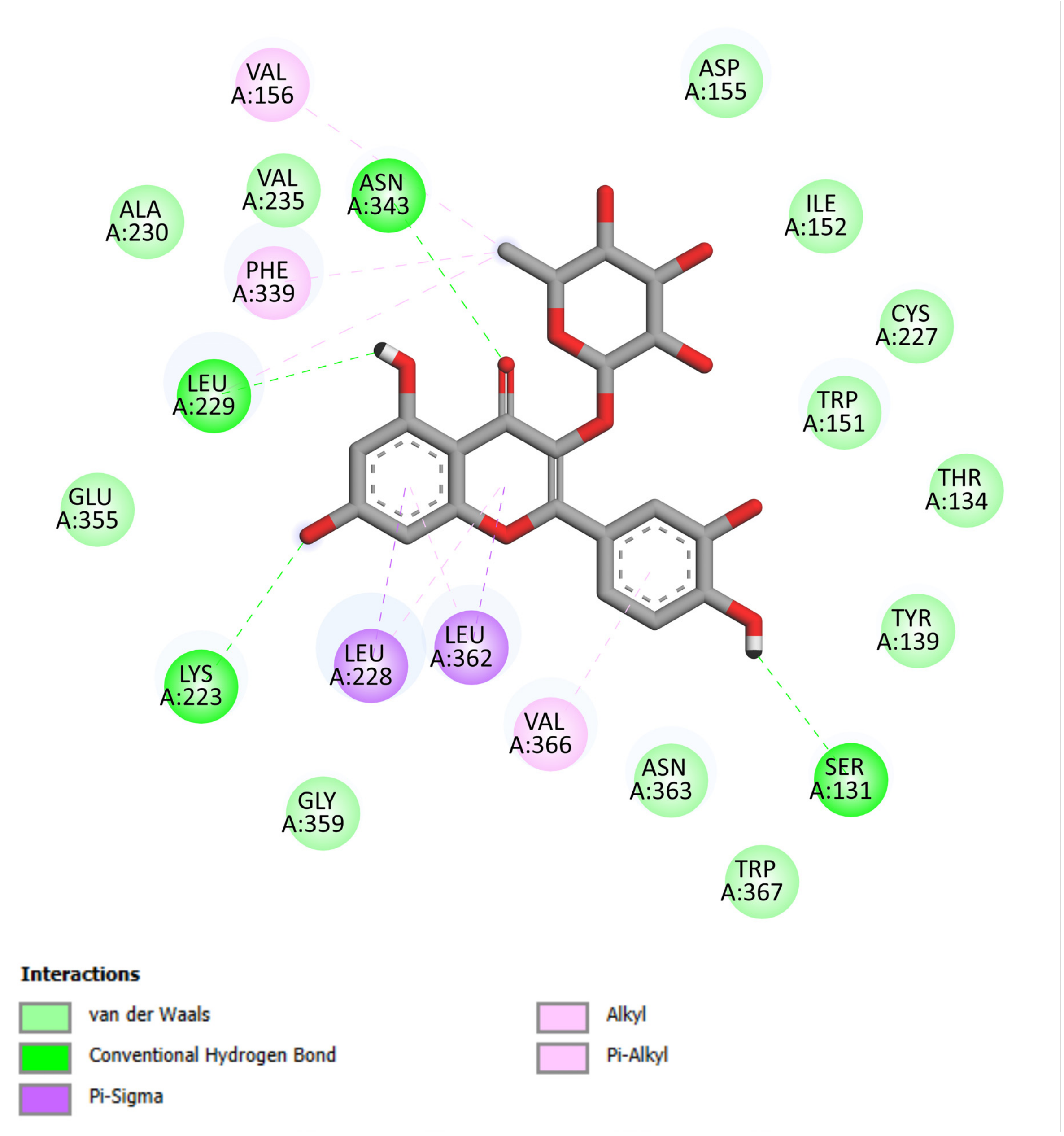

Supplement: Supplementary file 1 [file plants-13-01643-s001.zip › plants-3015746-supplementary.pdf]
